# Strength and Conditioning Society (SCS) 8th Annual Meeting, Oslo, Norway, 2025

**DOI:** 10.3390/sports14050199

**Published:** 2026-05-12

**Authors:** Pedro E. Alcaraz, Anthony J. Blazevich, Tomás T. Freitas, Elena Marín-Cascales, Truls Raastad

**Affiliations:** 1 UCAM Research Center for High Performance Sport, UCAM Universidad Católica de Murcia, 30107 Murcia, Spain; palcaraz@ucam.edu (P.E.A.); tfreitas@ucam.edu (T.T.F.); 2 Facultad de Deporte, UCAM Universidad Católica de Murcia, 30107 Murcia, Spain; 3 Strength & Conditioning Society, 30008 Murcia, Spain; a.blazevich@ecu.edu.au; 4 Centre for Exercise and Sports Science Research, School of Medical and Health Sciences, Edith Cowan University, Joondalup 6027, Australia; 5 NAR-Nucleus of High Performance in Sport, São Paulo 04753-060, Brazil; 6 Department of Physical Performance, Norwegian School of Sport Science, 0806 Oslo, Norway; trulsr@nih.no

**Keywords:** congress, performance, exercise, health, training, injury

## Abstract

On behalf of the Strength and Conditioning Society (SCS), we are pleased to present the abstracts submitted for the SCS 8th Annual Meeting. The event was held at the Norwegian School of Sport Sciences in Oslo, Norway, on 8–10 October 2025, and comprised several invited sessions held by international and national speakers on a variety of topics related to biochemistry and exercise physiology, strength and conditioning practices and their application to health, injury prevention, and sports performance. These included strength training in high-performance sports, sport science and training–competition load management in elite environments, biochemistry and exercise physiology and prescription, nutrition and biomechanics, among others. The conference also included practical workshops held by renowned academics and practitioners on eccentric training, change of direction ability, and strength and power training in professional team sports, combat sports, and ergospirometry and exercise prescription in specific populations. Finally, the event disseminated up-to-date strength and conditioning research by providing practitioners and researchers with the opportunity to present their most recent findings. All abstracts presented at the SCS 8th Annual Meeting can be found in this Conference Report.

## 1. Introduction

The Strength and Conditioning Society (SCS) is pleased to introduce the abstracts presented at the SCS 8th Annual Meeting. In accordance with the SCS’s vision and mission of disseminating high-quality evidence of the performance and health benefits of strength and conditioning practices worldwide, the 2025 Conference took place in Oslo, Norway, on 8–10 October. The SCS 8th Annual Meeting, held at the Norwegian School of Sport Sciences headquarters, brought together more than 200 attendees with different areas of expertise (e.g., sports science, sports physiotherapy, sports nutrition, exercise physiology and biochemistry, and sports medicine, among others), which provided ample opportunities to exchange and discuss the latest evidence on strength and conditioning practices from multiple perspectives. In a stimulating social and professional environment, practitioners and academics from different countries had the opportunity to attend several thought-provoking sessions provided by international and national speakers on a variety of applied topics related to (i) strength training in high-performance sports, children and the elderly; (ii) sport science and load management in elite environments; and (iii) injury prevention and rehabilitation process optimization in different injuries. As in previous years, the conference also offered multiple practical workshops by renowned academics and practitioners on strength and conditioning and nutrition in Ultimate Fighting Championship (UFC), cancer and strength training, strength and power training in team sports, or recovery strategies in high-performance sports. The event also fostered the dissemination of up-to-date strength and conditioning research by providing practitioners and researchers with the opportunity to present and discuss their latest findings, which can be found in the abstracts that compose this Conference Report. Finally, the SCS recognized professional and academic excellence in the field of strength and conditioning and presented the “Female Strength and Conditioning Coach of the Year Award”, the “Male Strength and Conditioning Coach of the Year Award”, the “Emerging Strength and Conditioning Coach of the Year Award” and the “Strength and Conditioning Coach Career Achievement Award” to outstanding coaches, and the “Young Investigator Award” (YIA) and the “Applied Science Award” (ASA) to remarkable researchers. It should be noted that the selection process for our award winners was entirely blinded, with a committee for both professional and academic awards. All the abstracts were reviewed by the Scientific Committee. For example, for each academic award (YIA and ASA), we selected 18 semifinalists, who presented their oral presentations under the supervision of a panel of three experts from the Scientific Committee. Among the 18 semifinalists, three finalists were chosen in each area, and they presented their work again on the final day of the event. The YIA finalists were the authors of the abstracts titled “Transitioning to 1500 m at LA2028: Impact of Duration on Energetic Contribution and Performance in Maximal Ergometer Rowing”, “Agreeing on One Intensity Scale: The Norwegian Endurance Sport Experience”, and “Comparison of Neural Determinants of Force Steadiness Improvements Following 8 weeks of Resistance Training in Young and Older Adults.” The ASA finalists were the authors of the abstracts titled “Twenty-four weeks of high load resistance exercise does not increase bone mineral density in wheelchair users: results of the BoneWheel study”; “Predictors of Response: Unraveling the Efficacy of Diet and Exercise Interventions in Type 2 Diabetes Mellitus”; and “Safety of Heavy Resistance Exercise During Pregnancy and Postpartum: A Controlled, Prospective Cohort Study.” The aforementioned committee selected the winners of the awards, which went to Peter Higgins from Ireland for the YIA and to Therese Fostervold Mathisen from Norway for the ASA.

## 2. Conference Abstracts

### 2.1. Microvascular Muscle Properties in Elite Power Lifters Versus 2 Endurance Athletes: More Similar than Different

Jonas S. Mathiesen ^1^, Ditte M. Berg-Sørensen ^1^, Jakob L. Nielsen ^1^ and Per Aagaard ^1,^*

^1^  Department of Sports Science and Clinical Biomechanics, Research Unit for Muscle Physiology and Biomechanics; University of Southern Denmark; Odense, Denmark
*  Correspondence: paagaard@health.sdu.dk

**Abstract**

Skeletal muscle capillarization is a key determinant of oxygen delivery and metabolic support during exercise. For optimal muscle function and to support athletic performance, sufficient muscle tissue perfusion is required. The effectiveness of oxygen, nutrient, and metabolite exchange with muscle fibers is fundamentally limited by the total surface area of the surrounding microvascular structures. Consequently, the structural organization of the capillary network is increasingly recognized as critically important for skeletal muscle function in both sports and health. Although muscle capillarization has mainly been studied in endurance athletes, it is also highly relevant for power athletes. The present study aimed to investigate skeletal muscle fiber cross-sectional area (mCSA) and capillarization in elite triathletes compared to elite power athletes and non-trained controls. It was hypothesized that triathletes would demonstrate a more proliferated capillary supply relative to fiber size, whereas power athletes would show larger muscle fiber areas. Muscle biopsies (vastus lateralis) obtained from elite triathletes (TRI, n = 7), elite power athletes (POW, n = 7) and age-matched recreationally active controls (CON, n = 7) were immunochemically stained and analyzed by a semi-automated pipeline. Samples were analyzed for mCSA, fiber type distribution, capillary number/density, and capillary contacts (CC). POW demonstrated greater mean mCSA and type II mCSA than CON (*p* < 0.01). TRI were characterized by a higher proportion of type I fibers (62 ± 16%) compared to POW (40 ± 15%) and CON (41 ± 13%). Denser capillarization was observed in TRI (CC: 8.9 ± 2.2) and POW (CC: 10.4 ± 4.0) compared to CON (CC: 4.9 ± 1.4), both when normalized to mCSA and fiber perimeter (*p* < 0.05). As the main finding, POW and TRI showed no differences in capillary supply in their vastus lateralis musculature. Elite triathletes demonstrated a higher proportion of type I fibers and a more proliferated capillary supply compared to recreationally active controls. Surprisingly, power athletes also showed elevated capillarization, especially in type II fibers, compared to controls. In summary, both POW and TRI demonstrated denser capillary-to-fiber matching compared to controls, suggesting both training modalities to be effective in evoking microvascular muscle adaptations. These findings challenge the traditional view that resistance training primarily induces hypertrophy with only minor effects on angiogenesis.

**References**

1. Hellsten, Y.; Gliemann, L. Peripheral limitations for performance: Muscle capillarization. Scand. J. Med. Sci. Sports 2024, 34(1), e14442.
2. Betz, M.W.; Aussieker, T.; Kruger, C.Q.; Gorissen, S.H.M.; Van Loon, L.J.C.; Snijders, T. Muscle fiber capillarization is associated with various indices of skeletal muscle mass in healthy, older men. Exp. Gerontol. 2021, 143, 111161.
3. Van Der Zwaard, S.; Brocherie, F.; Jaspers, R.T. Under the hood: Skeletal muscle determinants of endurance performance. Front. Sports Act. Living 2021, 3, 719434.
4. McIntosh, M.C.; Anglin, D.A.; Robinson, A.T.; Beck, D.T.; Roberts, M.D. Making the case for resistance training in improving vascular function and skeletal muscle capillarization. Front. Physiol. 2024, 15, 1338507.

**Disclaimer/Publisher’s Note:** The statements, opinions and data contained in all publications are solely those of the individual author(s) and contributor(s) and not of MDPI and/or the editor(s). MDPI and/or the editor(s) disclaim responsibility for any injury to people or property resulting from any ideas, methods, instructions or products referred to in the content.

### 2.2. Physical Performance and Home Field Advantage in Soccer

Laura Alberti Zandavalli *, Rafael Grazioli, Giovanni Sala Ramirez and Fábio Yuzo Nakamura y Eduardo Lusa Cadore ^1^

Universidade Federal do Rio Grande do Sul; laurazandavalli@outlook.com; gbsrmz@gmail.com; edcadore@yahoo.com.br, Universidade São Francisco; rafael_grazioli@hotmail.com Universidade da Maia; fnakamura@umaia.pt

*  Correspondence: laurazandavalli@outlook.com

**Abstract**

Modern soccer players are regularly required to travel both nationally and internationally, covering short and long distances to compete in matches. Consequently, athletes can play more than 70 matches in a single season, sometimes competing in three matches per week during congested periods, with insufficient recovery time. Such travel and the disruptions caused by frequent travel can reduce performance levels in away matches, particularly in metrics related to external load, and increase the risk of injury.

From this perspective, the aim of the present study is to describe and compare the external match load between home and away games, during both congested and non-congested weeks. The study included 40 professional soccer players competing in national-level events. GPS (Global Positioning System) metrics were recorded over 53 matches played from January to June across two seasons. Athletes had an average age of 27 ± 4 years, body mass of 79.05 ± 6.6 kg, and height of 178 ± 5.72 cm. Matches were categorized as congested (two matches per week) or non-congested (one match per week) and further classified as home or away games. The metrics analyzed included total distance, high-intensity distance (19.8–25 km/h), and sprint distance (>25 km/h), all normalized by minutes played. The Shapiro-Wilk test assessed normality, and paired *t*-tests compared time points; the Wilcoxon test was used for non-parametric data. In congested weeks, high-intensity distance significantly decreased in away matches (home: 4.35 ± 1.81 m·min^−1^; away: 3.97 ± 1.76 m·min^−1^; *p* = 0.012), but no significant differences were found in total distance (home: 97.47 ± 16.74 m·min^−1^; away: 96.38 ± 17.24 m·min^−1^; *p* = 0.528) or sprint distance (home: 1.30 ± 0.91 m·min^−1^; away: 1.22 ± 0.90 m·min^−1^). During non-congested weeks, all metrics were significantly reduced for away matches:

- Total distance: home 103.47 ± 11.50 m·min^−1^; away 99.48 ± 12.20 m·min^−1^; *p* = 0.004
- High-intensity distance: home 4.63 ± 1.75 m·min^−1^; away 3.97 ± 1.59 m·min^−1^; *p* = 0.004
- Sprint distance: home 1.61 ± 0.99 m·min^−1^; away 1.22 ± 0.83 m·min^−1^; *p* = 0.001

In summary, soccer players cover greater high-intensity distances at home, particularly during congested periods, as widely reported in the literature. In non-congested weeks, players also perform better in all GPS metrics at home. These findings highlight the influence of travel fatigue and other contextual factors on physical performance during away matches.

**Keywords:** travel fatigue; performance; workload; soccer

**Funding:** This research was funded by The National Council for Scientific and Technological Development (CNPq, Brazil) and the Coordination for the Improvement of Higher Education Personnel (CAPES, Brazil).

**References**

1. Janse van Rensburg, D.C.; et al. Managing Travel Fatigue and Jet Lag in Athletes: A Review and Consensus Statement. Sports Med 2021.
2. Mohr, M.; et al. Muscle damage, inflammatory, immune and performance responses to three football games in 1 week in competitive male players. Eur J Appl Physiol 2016.
3. Fowler, P.; Duffield, R.; Vaile, J. Effects of domestic air travel on technical and tactical performance and recovery in soccer. Int J Sports Physiol Perform 2014.
4. Lago, C. The influence of match location, quality of opposition, and match status on possession strategies in professional association football. J Sports Sci 2009.

**Disclaimer/Publisher’s Note:** The statements, opinions and data contained in all publications are solely those of the individual author(s) and contributor(s) and not of MDPI and/or the editor(s). MDPI and/or the editor(s) disclaim responsibility for any injury to people or property resulting from any ideas, methods, instructions or products referred to in the content.

### 2.3. The Effect of Aerobic and Resistance Training on Glycemic Control in Type 2 Diabetes: A Systematic Review and Meta-Analysis

Ramzi Ahmad Al-Horani ^1,^* and Bayan Wael Abdullah ^1^

^1^  Department of Sport Sciences, Yarmouk University, Jordan
*  Correspondence: raalhorani@yu.edu.jo

**Abstract:**

This study compared the effects of aerobic and resistance training on glycemic control factors in patients with type 2 diabetes. A systematic search of PubMed and CINAHL (May 2024) identified studies on aerobic and resistance training effects on glycemic control in adults with type 2 diabetes. Eligible studies included randomized, supervised interventions reporting outcomes such as HbA1c, fasting glucose, insulin, HOMA-IR, or adiposity measures. Study quality and risk of bias were assessed using the Cochrane Risk of Bias tool. Effect sizes were estimated as mean differences (pre-post) with 95% confidence intervals. A total of 20 studies were included, with 828 participants in the aerobic training group and 839 in the resistance training group. The overall pooled difference in HbA1c between resistance and aerobic training was 0.04% [−0.13, 0.2], with no statistical significance (*p* = 0.68). Sensitivity analysis excluding high-risk bias studies did not alter results. In subgroups, aerobic training significantly reduced HbA1c compared to resistance training in studies lasting >24 weeks (0.29% [0.05, 0.53], *p* = 0.02) and in participants with diabetes duration <7 years (0.27% [0.09, 0.44], *p* = 0.003). The pooled fasting glucose and insulin resistance index estimates were non-significant and highly variable. Insulin levels showed a significant reduction favoring aerobic training (8.42 [0.98, 15.58] pmol/L, *p* = 0.03) but with high variability. No significant differences were found for fat percentage, fat mass, LDL, HDL, or total cholesterol. Aerobic and resistance training effectively improves glycemic control in type 2 diabetes, particularly HbA1c. However, aerobic training shows greater benefits in long-term programs and in patients with a diabetes duration of <7 years. Given their complementary effects, both training types should be encouraged based on patient preference and integrated into general recommendations.

**Keywords:** HOMA-IR; Fasting glucose; Glycated hemoglobin; Insulin sensitivity; Endurance training; Strength training

**Disclaimer/Publisher’s Note:** The statements, opinions and data contained in all publications are solely those of the individual author(s) and contributor(s) and not of MDPI and/or the editor(s). MDPI and/or the editor(s) disclaim responsibility for any injury to people or property resulting from any ideas, methods, instructions or products referred to in the content.

### 2.4. Comparison Between the Effect of Aerobic and Resistance Exercise with Different Intensities on Post Exercise Blood Pressure Responses in Normotensive Subjects

Ramzi Ahmad Al-Horani ^1,^* and Thaer Ali Malkawi ^1^

^1^  Department of Sport Sciences, Yarmouk University, Jordan
*  Correspondence: raalhorani@yu.edu.jo

**Abstract**

The variation in resting blood pressure among individuals engaging in aerobic and/or resistance exercise training might be due to the acute drop in blood pressure after exercise. Therefore, this study aimed to compare the acute effect of resistance and aerobic exercise with different intensities on post-exercise blood pressure responses. Seven normotensive and physically active males participated in this study (age: 25.3 ± 6.2; Body mass index (BMI): 23.7 ± 2.6). Baseline blood pressure, heart rate (HR), heart rate reserve (HRR), and maximum strength (1 RM) of 6 resistance exercises were measured. Accordingly, participants performed two 40-min cycling sessions at 60% of HRR (C60) and 75% of HRR (C75), and two sessions of resistance exercise (6 exercises) at 60%1 RM (RE60) and 75% 1 RM (RE75) in random order. After each exercise session, participants sat for 60 min, during which blood pressure, HR, and the rate of perceived exertion (RPE) were measured at 0, 15-, 30-, 45-, and 60 min post-exercise. The anxiety state Y1 was obtained at 0-, 30-, and 60-min post-exercise. Systolic blood pressure decreased significantly at 15–60 min post C75 and 60 min post RE75. Diastolic blood pressure decreased at 0-, 15-, and 30-min post-RE60 and at 30 min post-RE75 only, and did not decrease after C60 and C75. Mean arterial pressure was decreased at 0 and 15 min post-RE60 and 15 post-RE75 without changes after C60 and C75. The decrease in mean arterial pressure was lower in RE60 and RE75 compared to C60 and C75. There were no changes in the state of anxiety Y1. We conclude that higher-intensity aerobic exercise has the greatest effect on lowering systolic blood pressure. In contrast, low-intensity resistance exercise is more effective in reducing diastolic blood pressure and mean arterial pressure.

**Keywords:** Systolic blood pressure; diastolic blood pressure; mean arterial pressure; aerobic training; resistance exercise

**Disclaimer/Publisher’s Note:** The statements, opinions and data contained in all publications are solely those of the individual author(s) and contributor(s) and not of MDPI and/or the editor(s). MDPI and/or the editor(s) disclaim responsibility for any injury to people or property resulting from any ideas, methods, instructions or products referred to in the content.

### 2.5. Effects of High-Intensity Interval Training and Moderate-Intensity Continuous Training on Plasma Exosomal miR-15b-5p and Glycated Haemoglobin in Individuals with Type 2 Diabetes: A Randomised Controlled Trial

Laura Ávila Cabeza de Vaca ^1,^*, Manuel Costilla ^1^, María Rebollo Ramos ^2^, Alberto Marín Galindo ^1^, Adrián Montes de Oca García ^1^, Juan Corral Pérez ^1^, Andrea González Mariscal ^1^, María Calderón Domínguez ^3^, Jesús Gustavo Ponce González ^1^ and Cristina Casals ^1^

^1^  ExPhy Research Group, Department of Physical Education, Instituto de Investigación e Innovación Biomédica de Cádiz (INiBICA), Universidad de Cádiz, Cádiz, Spain.; laura.avila@uca.es; manueljesus.costilla@uca.es; alberto.marin@uca.es; adrian.montesdeoca@uca.es; juan.corral@uca.es; andrea.gonzalez@uca.es; jesusgustavo.ponce@uca.es; cristina.casals@uca.es
^2^  ExPhy Research Group, Department of Nursing and Physiotherapy, Instituto de Investigación e Innovación Biomédica de Cádiz (INiBICA), University of Cadiz, Spain.; maria.rebollo@uca.es
^3^  Biomedicine, Biotechnology, and Public Health Department, Instituto de Investigación e Innovación Biomédica de Cádiz (INiBICA), University of Cadiz, Spain.; maria.calderon@uca.es
*  Correspondence: laura.avila@uca.es

**Abstract**

Exosomal miRNAs play a key role in metabolic regulation and are promising biomarkers of glycaemic control in metabolic disorders such as type 2 diabetes (T2D) (1). Among them, miR-15b-5p is known to target key components of insulin signalling, including IRS1 (2); however, its regulation in plasma exosomes and responsiveness to exercise remain underexplored. This study aimed to examine the effects of high-intensity interval training (HIIT) and moderate-intensity continuous training (MICT) on plasma exosomal miR-15b-5p expression and glycated haemoglobin (HbA1c), as well as the association between their respective changes (delta), in individuals with prediabetes and T2D. Sixty-two participants (32 females, 56.2 ± 6.7 years; BMI > 25 kg/m^2^) with prediabetes or T2D (HbA1c > 5.7%) were randomised into three groups: HIIT (n = 21), cycling 10 × 1’ intervals at 90% peak power, MICT (n = 21), cycling 50’ at 10% above the first ventilatory threshold, and control group (CG; n = 20). Both interventions lasted 12 weeks (3 sessions/week). Fasting blood plasma samples were collected before and after the intervention. The HbA1c was measured via High-Performance Liquid Chromatography. Plasma exosomal miR-15b-5p expression was analysed using quantitative PCR and the 2^(-ΔCq) method. A mixed factorial ANOVA (3 × 2) was used to assess the effects of group and time on plasma exosomal miR-15b-5p expression and HbA1c, followed by Bonferroni post hoc tests. Pearson’s correlation was used to analyse the relationship between delta plasma exosomal miR-15b-5p expression and delta HbA1c. A significant group × time effect was found for plasma exosomal miR-15b-5p expression (F_2,59_ = 4.528; η_p_^2^ = 0.133; *p* = 0.015), with HIIT and MICT reducing its expression compared to CG (*p* = 0.004). A significant group × time interaction was found for HbA1c (F_2,59_ = 3.230; η_p_^2^ = 0.100; *p* = 0.047), with HIIT showed a significant decrease in HbA1c (*p* = 0.008). Delta of plasma exosomal miR-15b-5p expression correlated positively with delta of HbA1c (r = 0.400; *p* = 0.001). Both HIIT and MICT led to a significant reduction in plasma exosomal miR-15b-5p expression after 12-week training, while only HIIT significantly reduced HbA1c levels. The decrease in plasma exosomal miR-15b-5p was positively correlated with improvements in glycaemic control, underscoring its potential as a biomarker responsive to exercise interventions in adults with prediabetes or T2D. These results suggest that exercise interventions can modulate plasma exosomal miR-15b-5p expression in adults with T2D. Given that miR-15b-5p is known to target IRS1, its downregulation with training may partly explain the exercise-induced enhancement of insulin sensitivity, particularly in HIIT. These findings reinforce the clinical relevance of HIIT as a time-efficient intervention for metabolic health in individuals with prediabetes and T2D.

**Keywords:** Extracellular vesicles; Physical training; Metabolic syndrome; Genetic; Obesity; Glucose

**Funding:** This research was funded by the Spanish Ministry of Science and Innovation, grant numbers PID2020-120034RA-I00 (APETEX project) and PID2019-110063RA-I00/AEI/10.13039/501100011033 (EDUGUTION project).

**References**

1. Castaño, C.; Novials, A.; Párrizas, M. Exosomes and diabetes. *Diabetes Metab. Res. Rev.* **2019**, *35*, e3107.
2. McGeary, S.E.; Lin, K.S.; Shi, C.Y.; Pham, T.; Bisaria, N.; Kelley, G.M.; Bartel, D.P. The biochemical basis of microRNA targeting efficacy. *Science* **2019**, *366*, eaav1741. https://doi.org/10.1126/science.aav1741.

**Disclaimer/Publisher’s Note:** The statements, opinions and data contained in all publications are solely those of the individual author(s) and contributor(s) and not of MDPI and/or the editor(s). MDPI and/or the editor(s) disclaim responsibility for any injury to people or property resulting from any ideas, methods, instructions or products referred to in the content.

### 2.6. Effects of Multicomponent Training on Neuromuscular Performance and Dynamic Balance of Acutely Hospitalized Older Individuals: Preliminary Results of a Randomized Clinical Trial

Marcelo Bandeira-Guimarães ^1^, Eduarda Blanco-Rambo ^1^, Nadyne Rubin ^1^, Antenor Barbosa Calandrini ^1^, Raísa Ferreira de Abreu ^1^, Emilio Hideyuki Moriguchi ^1^, Mikel López Saez de Asteasu ^2^, Mikel Izquierdo ^2^, Caroline Pietta-Dias ^1^ and Eduardo Lusa Cadore ^1,^*

^1^  Universidade Federal do Rio Grande do Sul, Brazil. bgbandeira@gmail.com, eduardarambo@gmail.com, nadynerubin@yahoo.com.br, antenorcalandrini@gmail.com, raisabreu@hotmail.com, emoriguchi@hcpa.edu.br, carolpieta@yahoo.com.br, edcadore@yahoo.com.br
^2^  Universidad Pública de Navarra, mikel.lopez.saezdeasteasu@gmail.com, mikel.izquierdo@gmail.com
*  Correspondence: edcadore@yahoo.com.br; Tel.: +55 (51) 99119-3651

**Abstract**

Short-term hospitalization can negatively impact neuromuscular performance and increase risk of falls in older adults. Although in-hospital multicomponent training has shown benefits for muscle function and functional capacity, there is limited evidence on the use of exercise protocols that require minimal equipment, which would be interesting considering the reality of public hospitals settings in development countries. This study aimed to compare the effects of a multicomponent training program based on the ViviFrail model with usual care on neuromuscular performance and dynamic balance in acutely hospitalized older adults. A total of 39 patients (mean age 82.5 ± 4.4 years), medically approved for participation by the geriatrics team at the Hospital de Clínicas de Porto Alegre, were randomly assigned to either a training group (TG) or a control group (CG). The TG followed a daily bedside exercise routine that included sit-to-stand movements, knee extensions with ankle weights, seated rows using elastic bands, handgrip exercises, balance tasks, and walking, with progressive increases in volume and intensity. Both groups continued to receive standard hospital care. Neuromuscular performance was evaluated through lower-limb maximal power using the 3 times sit-to-stand test (POT) and handgrip strength (HGS). Dynamic balance was assessed using the Timed Up and Go (TUG) test. All analyses were conducted using an intention-to-treat approach. Data were analyzed using Generalized Estimating Equations (GEE), considering group and time as factors. Significance was set at *p* < 0.05, and results are presented as absolute delta values. A significant time vs. group interaction was observed for POT (*p* = 0.002), with the TG showing a mean increase of Δ = +76.26 W (*p* = 0.031), while the CG showed a decrease of Δ = −78.88 W (*p* = 0.026). No significant changes were observed in HGS (ΔTG = +0.41 kgf; ΔCG = −0.73 kgf). For dynamic balance, a significant time × group interaction was also found in TUG performance (*p* = 0.008), with the TG improving (Δ = −11.67 s; *p* = 0.079) and the CG declining (Δ = +11.16 s; *p* = 0.042). In conclusion, a low-cost, bedside multicomponent training program delivered during acute hospitalization was effective in improving lower-limbs power and dynamic balance in older adults. These findings support the feasibility and potential impact of implementing simplified exercise interventions in public hospital settings to counteract the physical decline associated with hospitalization.

**Keywords:** Frailty; Functional capacity; Muscle strength; Muscle power; Oldest old

**Funding:** This research was funded by National Council for Scientific and Technological Development (CNPq) and by Coordination for the Improvement of Higher Education Personnel (CAPES).

**References**

1. Izquierdo, M. Prescripción de ejercicio físico. El programa Vivifrail como modelo. *Nutr Hosp* **2019**, *36*, 50–56.
2. Martínez-Velilla, N; Casas-Herrero, A; Zambom-Ferraresi, F. Effect of Exercise Intervention on Functional Decline in Very Elderly Patients During Acute Hospitalization: A Randomized Clinical Trial. *JAMA* **2019**, *179*, 28–36.
3. Cadore, E.L; Izquierdo, M; Martínez-Velilla, N. Identifying Clinically Meaningful Muscle Power Enhancements and Their Functional Correlates in Hospitalized Older Patients. *J Gerontol A Biol Sci Med Sci* **2024**, *79*, glae240.
4. Izquierdo, M; de Souto Barreto, P; Arai, H. Global consensus on optimal exercise recommendations for enhancing healthy longevity in older adults (ICFSR). *J Nutr Health Aging* **2025**, *29*, 100401.

**Disclaimer/Publisher’s Note:** The statements, opinions and data contained in all publications are solely those of the individual author(s) and contributor(s) and not of MDPI and/or the editor(s). MDPI and/or the editor(s) disclaim responsibility for any injury to people or property resulting from any ideas, methods, instructions or products referred to in the content.

### 2.7. Delayed Supercompensation in Strength and Power Peaks at Least 3.5 Weeks After Both Failure and Non-Failure High-Frequency Low-Load Blood Flow Restricted Training

Thomas Bjørnsen ^1,2,^*, Kolbjørn Lindberg ^3^, Fredrik Tonstad Vårvik ^3^, Shlomi Gerbi ^3^, Njål V. Sandnes ^3^, Henrik Mangseth ^3^, Tone Sørli ^3^, Gøran Paulsen ^4^, Mathias Wernbom ^5^, Sveinung Berntsen Stølevik ^3^ and Truls Raastad ^4^

^1^  Department of Education and Sport Science, University of Stavanger, Stavanger, Norway
^2^  Norwegian Olympic Federation, Oslo, Norway
^3^  Department of Sport Science and Physical Education, University of Agder, Kristiansand, Norway
^4^  Department of Physical Performance, Norwegian School of Sport Sciences, Oslo, Norway
^5^  Department of Health and Rehabilitation, University of Gothenburg, Gothenburg, Sweden
*  Correspondence: thomas.bjornsen@uis.no

**Abstract**

High-frequency low-load blood flow restricted resistance exercise (BFR-RE) has been observed to induce rapid gains in strength and hypertrophy with loads as low as 20% of 1 repetition maximum (1 RM) (1,2). However, both failure and non-failure (30-15-15-15 repetitions) BFR-RE protocols induced prolonged decrements in force in two recent studies (3,4) and thus seemed to be too strenuous. Therefore, the aim of the present study was to compare the effect of a failure protocol with gradual familiarization vs. a less strenuous non-failure protocol during high-frequency BFR-RE, on changes in muscle size, maximal strength and power. Nineteen untrained men and women (23.5 ± 6.1 yrs, 10 women) performed 4 sets of BFR-RE to failure with one leg («Failure») and not to failure with the other leg («Non-failure»; 20-10-10-10 repetitions) using knee-extensions at 20% of 1 RM. Fourteen sessions were distributed over two five-day blocks, separated by a ten-day rest period («Rest Week»). The failure leg was familiarized to BFR-RE in the first two sessions. Muscle size changes of *m.rectus femoris* (RF) and *m.vastus lateralis* (VL) was measured by ultrasonography. Knee extension strength was assessed by 1 RM, maximal isometric contraction (MVC) and maximal power («Power») at 30% of 1 RM. Measurements were conducted at baseline, in the Rest Week, and 3, 10, 17 and 24 days post BFR-RE (“Post3–24”). Only Failure induced ~5% decrements in MVC at Rest Week and Post3. Post-training, MVC (4–8%), 1 RM (~15%) and Power (18–20%) gradually increased after both protocols and peaked at Post24 (all *p* < 0.05). Throughout the intervention, Non-failure increased MVC by 3% (*p* = 0.001) and Power by 4% (*p* = 0.002) more than the Failure leg. In contrast, the Failure protocol induced greater increases in RF and VL size over the time course of the study compared to Non-failure (5–9% vs. 3–8%, respectively, all *p* < 0.01). In conclusion, the Non-failure protocol led to larger gains in MVC and Power, while no differences were observed in 1 RM. Notably, the increases in MVC, Power and 1 RM were delayed and peaked later than the previous studies by Nielsen et al. (2) and our group (3) (12 vs. 20 vs. 24 days of detraining, respectively). Together with the prolonged MVC decreases in Failure leg, the findings indicate that the Failure protocol were too stressful and hampered strength gains compared to the Non-failure protocol. The Failure protocol induced larger muscle size gains than Non-failure BFR-RE. However, the Failure protocol induced greater muscle swelling during the BFR-RE blocks, which in turn could have influenced the post measurements.

**Keywords:** Kaatsu; occlusion; ischemic exercise; muscle hypertrophy; overreaching; overtraining; hypoxia; fatigue

**Funding:** This research received no external funding.

**References**

1. Nielsen JL, Aagaard P, Bech RD, Nygaard T, Hvid LG, Wernbom M, Suetta C, Frandsen U. Proliferation of myogenic stem cells in human skeletal muscle in response to low-load resistance training with blood flow restriction. J Physiol. **2012** *Sep 1;590(17):4351–61.*
2. Nielsen JL, Frandsen U, Prokhorova T, Bech RD, Nygaard T, Suetta C, Aagaard P. Delayed Effect of Blood Flow-restricted Resistance Training on Rapid Force Capacity. *Med Sci Sports Exerc.* **2017** *Jun;49(6):1157–1167*.
3. Bjørnsen T, Wernbom M, Løvstad A, Paulsen G, D’Souza RF, Cameron-Smith D, Flesche A, Hisdal J, Berntsen S, Raastad T. Delayed myonuclear addition, myofiber hypertrophy, and increases in strength with high-frequency low-load blood flow restricted training to volitional failure. *J Appl Physiol (1985).* **2019** *Mar 1;126(3):578–592.*
4. Bjørnsen T, Wernbom M, Paulsen G, Berntsen S, Brankovic R, Stålesen H, Sundnes J, Raastad T. Frequent blood flow restricted training not to failure and to failure induces similar gains in myonuclei and muscle mass. *Scand J Med Sci Sports.* **2021** *Jul;31(7):1420–1439.*

**Disclaimer/Publisher’s Note:** The statements, opinions and data contained in all publications are solely those of the individual author(s) and contributor(s) and not of MDPI and/or the editor(s). MDPI and/or the editor(s) disclaim responsibility for any injury to people or property resulting from any ideas, methods, instructions or products referred to in the content.

### 2.8. Effectiveness of Combined Strength Training and Dance Classes vs. Combined Strength and Endurance Training on Neuromuscular Parameters of Older Adults: A Randomized Clinical Trial

Eduarda Blanco-Rambo ^1^, Nadyne Rubin ^1^, Marcelo Bandeira-Guimarães ^1^, Caroline Rosa Muraro ^1^, Débora Marques ^1^, Greyse Dornelles Mello ^1^, Andressa Fergutz ^1^, Antenor Calandrini ^1^, Caroline Pietta-Dias ^1^ and Eduardo Lusa Cadore ^1,^*

^1^  Universidade Federal do Rio Grande do Sul, Brazil. eduardarambo@gmail.com; nadynerubin@yahoo.com.br; bgbandeira@gmail.com; caroline.rmuraro@gmail.com; dedy.marques@hotmail.com; greysedornelles@gmail.com; andressaufrgs1@gmail.com; antenorcalandrini@gmail.com; carolpieta@yahoo.com.br; edcadore@yahoo.com.br
*  Correspondence: edcadore@yahoo.com.br, +55 (51) 99119 3651

**Abstract**

Sarcopenia affects a large portion of older adults, compromising intrinsic capacity and quality of life. Strength and power training are key strategies to slow or even reverse this condition. However, isolated strength training programs may not always appeal to this population. In this context, combining different training modalities may offer a more engaging and effective approach. This study aimed to compare the effects of combined strength and endurance training (SEG) and combined strength training with dance classes (SDG) on muscle strength, power output, and vastus lateralis muscle thickness in older individuals. Maximal strength was assessed through one-repetition maximum test (1 RM) on knee extension exercise. Maximal power output (Pmax) was evaluated at 30% and 70% of 1 RM using a linear displacement sensor. Vastus lateralis muscle thickness (VLMT) was assessed through ultrasonography. Training was conducted twice weekly for 12 weeks with progressive intensity and volume. Both interventions included strength training followed by walking (SEG) or dance classes (SDG), with equivalent session durations. Generalized Estimating Equations were used to assess the main effects for time, group, and time × group interaction. Pairwise comparisons were performed using the Minimum Significant Difference post-hoc test. Significance was set at *p* < 0.05. Analyses followed an intention-to-treat approach. Results are presented as mean changes (△%) and effect sizes (ES). Forty-four (81% women) participants (mean age = 69.1 years) were randomized into two groups (SEG n = 22; SDG n = 22). After intervention, there was a significant time effect for 1 RM, Pmax at 30% and 70%, as well as VLMT (*p* values < 0.001) in both groups, with no significant time × group interactions. Significant increases were observed for SEG in the 1 RM (△% = 26.8; ES = 0.9), Pmax30% (△% = 26.6; ES = 0.7), Pmax70% (△% = 24.4; ES = 0.6) and VLMT (△% = 7.5; ES = 0.3); and, for SDG in the 1 RM (△% = 27.7; ES = 0.6), Pmax30% (△% = 43.6; ES = 0.7), Pmax70% (△% = 41.7; ES = 0.8) and VLMT (△% = 9.6; ES = 0.4). Thus, both interventions were capable to improve neuromuscular variables. These findings suggest that integrating strength training with either walking or dance presents a promising strategy to preserve muscle function and delay the effects of sarcopenia in aging populations.

**Keywords:** Muscle strength; power output; Aging; Resistance training; Endurance training; Combined training; Dance classes

**Funding:** This research was funded by National Council for Scientific and Technological Development (CNPq) and by Coordination for the Improvement of Higher Education Personnel (CAPES).

**References**

1. Cadore, EL; Pinto, RS; Pinto, SS; et al. Effects of Strength, Endurance, and Concurrent Training on Aerobic Power and Dynamic Neuromuscular Economy in Elderly Men. J Strength Cond Res. 2011, 25, 758–766.
2. Blanco-Rambo, E; Izquierdo, M; Cadore, EL. Dance as an Intervention to Improve Physical and Cognitive Functioning in Older Adults. J Nutr Health Aging. 2023, 27, 75–76.
3. Blanco-Rambo, E; Rubin, N; Bandeira-Guimarães, M; et al. Efeitos do treinamento combinado tradicional e do treinamento multicomponente composto por treinamento de força e aulas de dança na capacidade funcional e cognitiva de idosos: protocolo de estudo. Rev. Bras. Ativ. Fís. Saúde. 2024, 29, 1–1.
4. Izquierdo M, de Souto Barreto P, Arai H, et al. Global consensus on optimal exercise recommendations for enhancing healthy longevity in older adults (ICFSR). *J Nutr Health Aging*. 2025;29(1):100401.

**Disclaimer/Publisher’s Note:** The statements, opinions and data contained in all publications are solely those of the individual author(s) and contributor(s) and not of MDPI and/or the editor(s). MDPI and/or the editor(s) disclaim responsibility for any injury to people or property resulting from any ideas, methods, instructions or products referred to in the content.

### 2.9. Impact of Multicomponent Exercise on Functional and Cognitive Performance in Acutely Hospitalized Older Adults: Preliminary Results of a Randomized Clinical Trial

Eduardo Lusa Cadore ^1,^*, Marcelo Bandeira-Guimarães ^1^, Eduarda Blanco-Rambo ^1^, Nadyne Rubin ^1^, Antenor Barbosa Calandrini ^1^, Raísa Ferreira de Abreu ^1^, Emilio Hideyuki Moriguchi ^1^, Mikel L. Saez de Asteasu ^2^, Mikel Izquierdo ^2^, Caroline Pietta-Dias ^1^ and Martim Bottaro ^3^

^1^  Universidade Federal do Rio Grande do Sul, Brazil. edcadore@yahoo.com.br; bgbandeira@gmail.com; eduardarambo@gmail.com; nadynerubin@yahoo.com.br; antenorcalandrini@gmail.com; raisabreu@hotmail.com; emoriguchi@hcpa.edu.br; carolpieta@yahoo.com.br.
^2^  Universidad Pública de Navarra, Spain. mikel.lopez.saezdeasteasu@gmail.com; mikel.izquierdo@gmail.com
^3^  Universidade de Brasília, martim@unb.br
*  Correspondence: edcadore@yahoo.com.br; Tel.: +5551 99119-3651

**Abstract**

Resistance exercise during acute hospitalization can mitigate the functional decline associated with prolonged bed rest in older adults. However, evidence on the effects of multicomponent exercise in the cognitive and physical functioning of hospitalized older adults in the context of public hospitals in developing countries remains limited. This study investigated the effects of Vivifrail-based multicomponent training versus usual care on physical and cognitive functioning in acutely hospitalized older adults. Eligible patients, identified by the geriatrics team at the Porto Alegre Clinical Hospital (Brazil) were invited to participate and randomly assigned to either a training group (TG) or a control group (CG). The TG followed a daily bedside exercise protocol consisting of strength/power and balance training, along with walking exercises. Exercise complexity was individualized based on a prior functional assessment, with daily progression in volume and intensity. Both groups continued to receive standard hospital care. Functional capacity was assessed using the Short Physical Performance Battery (SPPB) and the 4-Meter Gait Speed Test (4 mV), while cognitive function was evaluated using the Mini-Mental State Examination (MMSE). Data were analyzed using Generalized Estimating Equations (GEE), with group and time as factors. An intention-to-treat analysis approach was adopted. Statistical significance was set at *p* < 0.05, and results are presented as absolute delta values. Analyses were performed using SPSS v23. A total of 39 older adults participated (mean age 82.5 ± 4.4 years). No significant baseline differences were observed between groups. The average length of hospital stay was 5.2 days for the TG and 6.9 days for the CG (*p* = 0.079). A significant group × time interaction was found for the SPPB (*p* = 0.004), with the TG demonstrating a significant mean improvement of Δ = +1.22 points (*p* = 0.029), while the CG showed a significant decline of Δ = −1.73 points (*p* = 0.047). Similarly, a significant interaction was observed for 4 mV (*p* = 0.017), with the TG showing a non-significant improvement by Δ = +0.011 m/s (*p* = 0.181), whereas the CG experienced a significant decline of Δ = −0.20 m/s (*p* = 0.046). Regarding cognitive performance, a significant group × time interaction was also identified for the MMSE (*p* = 0.049), with the TG showing non-significant improvement (Δ = +1.22 points; *p* = 0.111) and the CG showing a slight non-significant decline (Δ = −0.94 points; *p* = 0.232). Multicomponent training based on the Vivifrail model significantly improved functional performance and appears to increase cognitive capacity in acutely hospitalized older adults. These findings support the implementation of structured, low-cost exercise interventions in hospital settings to help counteract hospitalization-associated declines.

**Keywords:** Frailty; Functional capacity; Cognitive function; Oldest old

**Funding:** This research was funded by National Council for Scientific and Technological Development (CNPq) and by Coordination for the Improvement of Higher Education Personnel (CAPES).

**References**

1. Izquierdo, M. Prescripción de ejercicio físico. El programa Vivifrail como modelo. *Nutr Hosp* **2019**, *36*, 50–56.
2. Martínez-Velilla, N; Casas-Herrero, A; Zambom-Ferraresi, F. Effect of Exercise Intervention on Functional Decline in Very Elderly Patients During Acute Hospitalization: A Randomized Clinical Trial. *JAMA* **2019**, *179*, 28–36.
3. Cadore, E.L; Izquierdo, M; Martínez-Velilla, N. Identifying Clinically Meaningful Muscle Power Enhancements and Their Functional Correlates in Hospitalized Older Patients. *J Gerontol A Biol Sci Med Sci* **2024**, *79*, glae240.
4. Izquierdo, M; de Souto Barreto, P; Arai, H. Global consensus on optimal exercise recommendations for enhancing healthy longevity in older adults (ICFSR). *J Nutr Health Aging* **2025**, *29*, 100401.

**Disclaimer/Publisher’s Note:** The statements, opinions and data contained in all publications are solely those of the individual author(s) and contributor(s) and not of MDPI and/or the editor(s). MDPI and/or the editor(s) disclaim responsibility for any injury to people or property resulting from any ideas, methods, instructions or products referred to in the content.

### 2.10. Predictors of Response: Unraveling the Efficacy of Diet and Exercise Interventions in Type 2 Diabetes Mellitus

Juan Corral-Pérez ^1,^*, Adrián Montes-de-Oca-García ^1^, Alberto Marín-Galindo ^1^, José D. Santotoribio ^1,2^, María Rebollo-Ramos ^3^, Manuel Costilla ^1^, Laura Ávila-Cabeza-de-Vaca ^1^, Andrea González-Mariscal ^1^, Jesus G. Ponce Gonzalez ^1^ and Cristina Casals ^1^

^1^  ExPhy Research Group, Department of Physical Education, Instituto de Investigación e Innovación Biomédica de Cádiz (INiBICA), Universidad de Cádiz, Cádiz, Spain., juan.corral@uca.es; adrian.montesdeoca@uca.es; alberto.marin@uca.es; manueljesus.costilla@uca.es; laura.avila@uca.es; andrea.gonzalez@uca.es; jesusgustavo.ponce@uca.es; cristina.casals@uca.es
^2^  Department of Laboratory Medicine, Puerto Real University Hospital, Cadiz, Spain. josed.santotoribio.sspa@juntadeandalucia.es
^3^  ExPhy Research Group, Department of Nursing and Physiotherapy, Instituto de Investigación e Innovación Biomédica de Cádiz (INiBICA), University of Cadiz, Spain.; maria.rebollo@uca.es
*  Correspondence: juan.corral@uca.es

**Abstract**

Lifestyle interventions, particularly dietary modifications and exercise, are primary recommendations for managing type 2 diabetes mellitus (T2DM) due to their positive impact on glycemic control (1,2); however, understanding why some individuals respond to these interventions (responders) while others do not (non-responders) remain an active research area. This study aimmined to identify baseline predictors of glycemic response, defined as a reduction in glycated haemoglobin (HbA1c), in adults with prediabetes or T2DM undergoing various lifestyle interventions. In this randomized trial, 109 participants with prediabetes or T2DM (54 women, mean age 56.5 ± 6.6 years) underwent a 12-week intervention. They were randomly assigned to high-intensity interval training (HIIT, n = 22), moderate-intensity continuous training (MICT, n = 21), a 300 kcal/day energy deficit diet (DIET, n = 21), HIIT combined with diet (HIIT + DIET, n = 25), MICT combined with diet (MICT + DIET, n = 20). Exercise involved HIIT (1-min intervals at 90% peak power, 3×/week) or MICT (50 min at 10% above ventilatory threshold, 3×/week). Diet groups received an individualized weekly menu revised biweekly to ensure adherence and weight loss. Baseline measurements included body composition (absolute body fat, fat-free mass, and percentages) via bioelectrical impedance, glycemic control (basal glucose/insulin/HbA1c), and cardiorespiratory fitness (maximal fat oxidation [MFO], intensity at MFO [FatMax], and maximal oxygen uptake [VO_2_max]) via indirect calorimetry. The participant was a responder if their change in HbA1c was two times higher than the inactive control group’s (n = 21) typical error of measurement. Logistic regressions with 10,000 samples bootstrapping identified predictors. A total of 57 participants were classified as responders: HIIT (n = 11), MICT (n = 7), DIET (n = 14), HIIT + DIET (n = 11), and MICT + DIET (n = 14). Higher baseline body fat percentage predicted non-response in the DIET group (OR: 0.865, 95% CI: 0.742–0.988, *p* = 0.015). Higher baseline absolute fat-free mass was associated with a lower likelihood of response in the HIIT group (OR: 0.925, 95% CI: 0.850–0.999, *p* = 0.023), but appeared positively associated with response in the DIET group (OR: 1.131, 95% CI: 1.001–1.281, *p* = 0.021). In the DIET group, baseline VO_2_max (OR: 1.310, 95% CI: 1.001–1.766, *p* = 0.034) and FatMax (OR: 1.146, 95% CI: 1.001–1.372, *p* = 0.046) were also significant predictors of HbA1c improvements. Higher baseline fat-free mass and cardiorespiratory fitness increased the likelihood of dietary response, while higher baseline body fat may hinder it. Notably, greater baseline fat-free mass was negatively associated with the glycemic response to HIIT, suggesting that individuals with more lean mass may benefit less from this modality.

**Keywords:** Insulin Resistance; Metabolic Syndrome; Disease Management; Nutritional Counseling; Biomarkers

**Funding:** Grants:1)PID2019-110063RA-I00/AEI/10.13039/501100011033.2)PID2020-120034RA-I00/AEI/10.13039/501100011033.3)EXP_74977.

**References**

1. Kirwan, J.P.; Heintz, E.C.; Rebello, C.J.; Axelrod, C.L. Exercise in the prevention and treatment of type 2 diabetes. Compr. Physiol. 2023, 13, 4559–4585.
2. Pozzilli, P.;Falluca, F. Diet and diabetes: a cornerstone for therapy. Diabetes Metab Res Rev. 2014, 30, Suppl 1:1–3.

**Disclaimer/Publisher’s Note:** The statements, opinions and data contained in all publications are solely those of the individual author(s) and contributor(s) and not of MDPI and/or the editor(s). MDPI and/or the editor(s) disclaim responsibility for any injury to people or property resulting from any ideas, methods, instructions or products referred to in the content.

### 2.11. Effects of Two Aerobic Exercise Modalities and Diet on Skeletal Muscle Mitochondrial Content and Respiration in Individuals with Type 2 Diabetes

Manuel Costilla ^1,^*, Juan Corral-Pérez ^1^, Laura Ávila-Cabeza-de-Vaca ^1^, Andrea González-Mariscal ^1^, Alberto Marín-Galindo ^1^, Adrián Montes-de-Oca-García ^1^, José D. Santotoribio ^1,2^, Steen Larsen ^3^, Jesus G. Ponce-Gonzalez ^1^ and Cristina Casals ^1^

^1^  ExPhy Research Group, Department of Physical Education, Instituto de Investigación e Innovación Biomédica de Cádiz (INiBICA), Universidad de Cádiz, Cádiz, Spain; manueljesus.costilla@uca.es; juan.corral@uca.es; laura.avila@uca.es; andrea.gonzalez@uca.es; alberto.marin@uca.es; adrian.montesdeoca@uca.es; josed.santotoribio.sspa@juntadeandalucia.es; jesusgustavo.ponce@uca.es; cristina.casals@uca.es
^2^  Department of Laboratory Medicine, Puerto Real University Hospital, Cádiz, Spain; josed.santotoribio.sspa@juntadeandalucia.es
^3^  Department of Biomedical Sciences, Faculty of Health and Medical Sciences, University of Copenhagen, Copenhagen, Denmark; stelar@sund.ku.dk
*  Correspondence: manueljesus.costilla@uca.es

**Abstract**

Although the link between insulin resistance and mitochondrial content or respiration remains unclear (1), the impact of different interventions on these mitochondrial features in individuals with T2D is not fully researched. Dietary and exercise-induced weight loss improves insulin sensitivity; however, mitochondrial content enhancements appear to be driven mainly by exercise, while diet alone has no effect or may even reduce it (2). The comparative effects of training modality (moderate-intensity continuous (MICT) vs. high-intensity interval (HIIT) training) on mitochondrial respiration in T2D have been only examined in mice (3), and their combined impact with diet remains unresearched. Hence, this preliminary analysis aimed to assess the effect of MICT, HIIT, and diet on skeletal muscle mitochondrial content and respiration in T2D patients. This 12-week randomized controlled trial was conducted in 103 (51 women) T2D patients (56.6 ± 6.4 years, 6.8 ± 1.1% Hb1Ac, 33.1 ± 5.6 kg/m^2^) allocated in a MICT, HIIT, Diet, MICT plus Diet (MICT + Diet), HIIT plus Diet (HIIT + Diet) or a Control (CG) group. The MICT and HIIT consisted of three sessions per week on a cyclo-ergometer of 50 min at 10% above the ventilatory threshold and 10 sets of 1-min intervals at 90% with 1-min rest at 20% of the peak power output, respectively. The diet intervention consisted of an individualized weekly menu revised every two weeks with a calorie deficit of approximately 300 kcal. Before and after the intervention a biopsy from the *vastus lateralis* was obtained. The citrate synthase (CS) activity was measured spectrophotometrically, and the mitochondrial respiration was assessed in permeabilized fibers through high-resolution respirometry. A substrate titration protocol was used to assess the oxidative phosphorylation of the electron transport chain complex I (CI-OXPHOS) and complex I + II (CI + II-OXPHOS). The oxygen flux was normalized to CS activity to assess the intrinsic CI-OXPHOS (CI-INTRINSIC) and CI + II-OXPHOS (CI + II-INTRINSIC). A 2 (time)-by-3 (exercise)-by-2 (diet) mixed factorial ANOVA with Bonferroni post-hoc comparisons was performed. A significant time-by-exercise interaction were found for CS activity (F(2, 100) = 33.4, *p* < 0.001, ${\eta_{p}}^{2}$ = 0.408), CI-OXPHOS (F(2, 100) = 4.5, *p* = 0.014, ${\eta_{p}}^{2}$ = 0.085), and CI + II-OXPHOS (F(2, 100) = 7.3, *p* = 0.001, ${\eta_{p}}^{2}$ = 0.132) with significant increases (*p* < 0.05) in MICT, HIIT, MICT + Diet, and HIIT + Diet in comparison with the CG. No effects were found in CI-INTRINSIC and CI + II-INTRINSIC. Neither diet, time-by-diet, nor time-by-diet-by-exercise effect was observed in any outcome. In conclusion, both MICT and HIIT increased mitochondrial content but did not affect intrinsic mitochondrial respiration whereas diet had no effect either in mitochondrial content or respiration in T2D patients after a 12-week intervention.

**Keywords:** glucose metabolism; obesity; Mediterranean diet; metabolic syndrome; overweight; oxygen consumption; mitochondrial function

**Funding:** This research was funded by the Spanish Ministry of Science and Innovation (10.13039/501100011033; PID2019-110063RA-I00, PID2020-120034RA-I00) and INiBICA (PP11-007-2023).

**References**

1. Boushel, R.; Gnaiger, E.; Schjerling, P.; Skovbro M.; Kraunsøe, R.; Dela, F. Patients with type 2 diabetes have normal mitochondrial function in skeletal muscle. *Diabetologia*. 2007, 50, 790–796.
2. Toledo, D.; Menshikova, E.; Azuma, K.; Radiková, Z.; Kelley, C.; Ritov, V.; Kelley, D.; Mitochondrial Capacity in Skeletal Muscle Is Not Stimulated by Weight Loss Despite Increases in Insulin Action and Decreases in Intramyocellular Lipid Content. *Diabetes*. 2008, 57, 987–994.
3. Chavanelle, V.; Boisseau, N.; Otero, Y.; Combaret, L.; Dardevet, D.; Montaurier, C.; Delcros, G.; Peltier, S.; Sirvent, P. Effects of high-intensity interval training and moderate-intensity continuous training on glycaemic control skeletal muscle mitochondrial function in db/db mice. *Sci Rep*. 2017, 7, 204.

**Disclaimer/Publisher’s Note:** The statements, opinions and data contained in all publications are solely those of the individual author(s) and contributor(s) and not of MDPI and/or the editor(s). MDPI and/or the editor(s) disclaim responsibility for any injury to people or property resulting from any ideas, methods, instructions or products referred to in the content.

### 2.12. The Correlation Between Net Impulse and Phases of Linear Sprint Performance in University American Football Players

**Jack Davies ^1^, Max Cooper ^1^, Jonathan Hughes ^2^ and Craig Barden ^1,3^**

^1^  South Gloucestershire and Stroud College; jack.daves@sgscol.ac.uk, max.cooper@sgscol.ac.uk
^2^  Cardiff Metropolitan University; jdhughes@cardiffmet.ac.uk
^3^  University of Gloucestershire; cbarden@glos.ac.uk
*  Correspondence: jack.davies@sgscol.ac.uk

**Abstract**

Impulse has been proposed as a reliable performance measure when assessing static force generating capacity, during a given time frame, and in recent literature has been investigated to its degree of relationship with sprint performance. This study investigated the correlation between early epochs of net impulse from isometric actions and selected phases of linear sprint performance. A within subject design was employed to assess the correlation between linear sprint performance and epochs of net impulse in 29 university American Football athletes (mean ± SD: age = 20.10 ± 1.53 years; height = 181.69 ± 5.63 cm; weight = 95.92 ± 22.81 kg). Net impulse was measured over epochs of 0–100, 0–150, and 0–200 ms (Newtons per second) via an isometric mid-thigh pull protocol while linear speed was assessed using linear sprint testing (10 and 36.58 m). Pearson’s r correlation coefficient was used to evaluate correlations between assessed variables and effect size. The analysis demonstrated trivial to small correlations (r = −0.06 to 0.18) between early epochs of net impulse and linear sprint performance. The 10-metre linear sprint performance demonstrated small correlations at 0–150 ms (r = 0.18) while the remaining variables revealed trivial correlations. The effect size indicated trivial to small correlations between the assessed variables. Although no significant correlation was found between isometric mid-thigh pull metrics and linear sprint performance these findings provide meaningful insight into the complexity of sprinting mechanics, in the assessed population. If net impulse does not strongly correlate with sprint performance, it may indicate the need to reassess the emphasis placed on isometric strength assessments in sprint profiling. Instead, practitioners might consider integrating dynamic force-time metrics, reactive strength measures, and sport-specific sprint assessments to better inform training interventions. 

**Keywords:** Force-time Characteristics; Acceleration; Team Sport; Physical Profiling

**Funding:** This research received no external funding.

**References**

1. Brady, C. J., Harrison, A., Flanagan, E. P., Haff, G., & Comyns, T. M. (2020). The relationship between isometric strength and sprint acceleration in The relationship between isometric strength and sprint acceleration in sprinters sprinters. *Journal of Sports Physiology and Performance*, *1*, 38–45.
2. Comfort, P., Dos’Santos, T., Beckham, G. K., Stone, M. H., Guppy, S. N., & Haff, G. G. (2019). Standardization and methodological considerations for the isometric midthigh pull. *Strength and Conditioning Journal*, *41*(2), 57–79.
3. McCormick, B., Talpey, S., James, L., & MacMahon, C. (2022). The influence of instruction on isometric mid-thigh pull force-time variables. *International Journal of Strength and Conditioning*, *2*(1).
4. Thomas, C., Comfort, P., Chiang, C.-Y., & Jones, P. A. (2015). Relationship between isometric mid-thigh pull variables and sprint and change of direction performance in collegiate athletes. In *Journal of Trainology* (Vol. 4).

**Disclaimer/Publisher’s Note:** The statements, opinions and data contained in all publications are solely those of the individual author(s) and contributor(s) and not of MDPI and/or the editor(s). MDPI and/or the editor(s) disclaim responsibility for any injury to people or property resulting from any ideas, methods, instructions or products referred to in the content.

### 2.13. Methodology and Training Resources Used by Strength & Conditioning Coaches in Semi-Professional Football Teams in Spain

José C. Díaz del Campo ^1,^*, Pedro E. Alcaraz ^1,2^, Julio Calleja-González ^3^ and Tomás T. Freitas ^2^

^1^  UCAM Spanish Sports University, Catholic University of Murcia, Murcia 30107, Spain; jcdiaz@ucam.edu
^2^  UCAM Research Center for High Performance Sport, Catholic University of Murcia, Murcia 30107, Spain; palcaraz@ucam.edu (P.E.A.); tfreitas@ucam.edu (T.T.F.)
^3^  Department of Physical Education and Sport, Faculty of Education and Sport, University of the Basque Country (EHU), 01007 Vitoria-Gasteiz, Spain; julio.calleja.gonzalez@gmail.com (J.C.-G.)
*  Correspondence: jcdiaz@ucam.edu

**Abstract**

It is becoming more common for semi-professional football teams in Spain to have a qualified strength & conditioning coach on their coaching staff to plan the conditional development of the players in these teams. In their aim to improve the planning and training processes in these teams, they rely on the existing bibliography related to professional or elite football teams. The aim of this study is to identify and analyze the methods and means used by physical trainers in semi-professional football, the human and material resources available for the development of the training process and the academic profile of those responsible for physical preparation in these teams.

In order to obtain this information, ninety-seven physical trainers or coaches of 2nd RFEF and 3rd RFEF teams that are not subsidiaries or teams affiliated to a professional club (LaLiga EA Sports, LaLiga Hypermotion and 1st RFEF), carried out an online survey of 50 questions, divided into 8 sections: (a) personal information, (b) material resources, space and time available, (c) strength and power training, (d) speed and plyometric training, (e) aerobic capacity training, (f) mobility training, (g) assessment and control, (h) training programming. Frequency analysis has been used to evaluate and count responses.

All respondents had a university education in sports science. Only 21% have strength training-related certification and 84% have any football-related certification. 39% train four times a week, with 7% of teams not having a full field for training on any day of the week. Eccentric (90%), concentric (84%) and variable (82%) are the most common resistance methods in strength training, with squat and core stability exercises (94%) being the most used exercises. Plyometrics (87%) is the most common method for speed training. For endurance development, the most repeated strategies are the played tasks (92%) and medium-sided games (83%). The most used methods for flexibility development are dynamic (74%) and ballistic (68%).

97% of the respondents claim to use some kind of strategy for monitoring the condition of the players. The individualization of training, the improvement of human, technological and material resources are issues that concern physical trainers nowadays. With this information we obtain a clear reflection of how non-professional football is trained in Spain. By analyzing the results, the physical trainers of these teams can use the information from this study to learn about current practices and get ideas for the future design of new training programs.

**Keywords:** Survey; amateur; performance; team sports

**Funding**: This research received no external funding.

**References**

1. Loturco, I.; Freitas, T.T.; Alcaraz, P.E.; Kobal, R.; Hartmann Nunes, R.F.; Weldon, A.; Pereira, L.A. Practices of Strength and Conditioning Coaches in Brazilian Elite Soccer. Biol. Sport 2022, 39, 779–791, doi:10.5114/biolsport.2022.108703.
2. Weldon, A.; Duncan, M.J.; Turner, A.; Sampaio, J.; Noon, M.; Wong, D.P.; Lai, V.W. Contemporary Practices of Strength and Conditioning Coaches in Professional Soccer. Biol. Sport 2021, 38, 377–390, doi:10.5114/BIOLSPORT.2021.99328.
3. Weldon, A.; Duncan, M.J.; Turner, A.; LaPlaca, D.; Sampaio, J.; Christie, C.J. Practices of Strength and Conditioning Coaches: A Snapshot From Different Sports, Countries, and Expertise Levels. J. Strength Cond. Res. 2020, doi:10.1519/JSC.0000000000003773.
4. Weldon, A.; Duncan, M.J.; Turner, A.; Lockie, R.G.; Loturco, I. Practices of Strength and Conditioning Coaches in Professional Sports: A Systematic Review. Biol. Sport 2022, 39, 715–726, doi:10.5114/BIOLSPORT.2022.107480.

**Disclaimer/Publisher’s Note:** The statements, opinions and data contained in all publications are solely those of the individual author(s) and contributor(s) and not of MDPI and/or the editor(s). MDPI and/or the editor(s) disclaim responsibility for any injury to people or property resulting from any ideas, methods, instructions or products referred to in the content.

### 2.14. Physical Performance Variables in Semi-Professional Football: A Systematic Review

José C. Díaz del Campo ^1,^*, Pedro E. Alcaraz ^1,2^, Julio Calleja-González ^3^ and Tomás T. Freitas ^2^

^1^  UCAM Spanish Sports University, Catholic University of Murcia, Murcia 30107, Spain; jcdiaz@ucam.edu
^2^  UCAM Research Center for High Performance Sport, Catholic University of Murcia, Murcia 30107, Spain; palcaraz@ucam.edu (P.E.A.); tfreitas@ucam.edu (T.T.F.)
^3^  Department of Physical Education and Sport, Faculty of Education and Sport, University of the Basque Country (EHU), 01007 Vitoria-Gasteiz, Spain; julio.calleja.gonzalez@gmail.com (J.C.-G.)
*  Correspondence: jcdiaz@ucam.edu

**Abstract**

The control of different variables that affect performance in football is key for strength & conditioning coaches to be able to program training and reduce injury risk factors. Even so, these strategies and variables are more often analyzed in elite football. The aim of this systematic review is to identify the performance variables that are referenced in the literature for semi-professional football teams. Three electronic databases (PubMed, Scopus and Web of Science) were consulted for a search up to 12 April 2025. A total of 98 articles were obtained. After eliminating duplicates (45) and adding by other sources (10), several were excluded for the following reasons: analysis of other sports (10); analysis of data in young people (8); professional players (3); women (2); simulated match protocols (2); referees (1); staff surveys (1); study of weather conditions (1); and analysis of monitoring devices (1). Finally, a total of 34 articles were included in this systematic review.

The results indicate that the variables were obtained in training (19), in match (8) and in match and training (7). The variables considered to analyze the external load are related to the total distance covered (21), the distance covered in different speed zones (20), accelerations and decelerations (27) and sprinting (15). As for the internal load variables, heart rate (13) and perceived exertion (12) are the most common. Different variables of mental workload and wellness have also been identified (9).

The studies analyzed show that small-sided games (SSG) are the most widely used and effective tool for evaluating the physical load in semi-professional football, allowing intensity to be modulated according to format and rules. It is evident that variables such as playing position, match context and recovery have a significant influence on performance. Furthermore, technology and predictive models emerge as key tools for individualized planning, performance optimization and injury prevention in semi-professional football.

**Keywords**: Internal load; external load; players; male; indicators

**Funding**: This research received no external funding.

**References**

1. Asian-Clemente, J.A.; Rabano-Muñoz, A.; Núñez, F.J.; Suarez-Arrones, L. External and Internal Load during Small-Sided Games in Soccer: Use or Not Floaters. J. Sports Med. Phys. Fitness 2022, 62, 301–307, doi:10.23736/S0022-4707.21.12103-6.
2. David, C.; Julen, C. The Relationship Between Intensity Indicators in Small-Sided Soccer Games. J. Hum. Kinet. 2015, 46, 119–128, doi:10.1515/hukin-2015-0040.
3. Hernández, D.; Sánchez, M.; Martin, V.; Benéitez-Andrés, E.; Sánchez-Sánchez, J. Variables Contextuales y Carga Externa Semanal En Un Equipo de Fútbol Semiprofesional. Apunt. Educ. Física y Deport. 2021, 61–67, doi:10.5672/apunts.2014-0983.es.(2021/4).146.07.
4. Swallow, W.E.; Skidmore, N.; Page, R.M.; Malone, J.J. An Examination of In-Season External Training Load in Semi-Professional Soccer Players: Considerations of One and Two Match Weekly Microcycles. Int. J. Sports Sci. Coach. 2020, 16, 192–199, doi:10.1177/1747954120951762.

**Disclaimer/Publisher’s Note:** The statements, opinions and data contained in all publications are solely those of the individual author(s) and contributor(s) and not of MDPI and/or the editor(s). MDPI and/or the editor(s) disclaim responsibility for any injury to people or property resulting from any ideas, methods, instructions or products referred to in the content.

### 2.15. Resistance Exercise Strategies for Enhancing Functional Capacity in Patellofemoral Pain: A Systematic Review

Pedro Diez-Solorzano ^1,2^, Miguel Heres ^2,3^, Marcos Quintana-Cepedal ^1,2^, Miguel del Valle ^2,4^, Irene Crespo ^1^ and Hugo Olmedillas ^1,2,^*

^1^  Department of Functional Biology, University of Oviedo, Oviedo, Spain; diezsolorzano.p@gmail.com (P.D.-S.); marcosquintana99@gmail.com (M.Q.-C.); crespoirene@uniovi.es (I.C.); olmedillashugo@uniovi.es (H.O.)
^2^  Asturian Research Group in Performance, Readaptation, Training and Health (AstuRES), University of Oviedo, Oviedo, Spain; miva@uniovi.es (M.V.)
^3^  Faculty of Health Sciences, Universidad Europea del Atlántico, Santander, Spain; miguel.heres@uneatlantico.es (M.H.)
^4^  Department of Cellular Morphology and Biology, University of Oviedo, Oviedo, Spain
*  Correspondence: olmedillashugo@uniovi.es

**Abstract**

Patellofemoral pain syndrome (PFPS) is a common musculoskeletal condition characterized by diffuse pain around or behind the patella, exacerbated by activities involving axial loading and knee movement, such as walking, running, or stair climbing [1]. This condition can substantially limit physical and athletic activity, negatively affecting patients’ quality of life [2]. Although resistance exercise is widely recognized as the most effective therapeutic approach [3], conclusive evidence regarding the optimal selection of specific exercise modalities remains lacking. This limitation hinders the development of standardized, evidence-based clinical protocols. To address this gap, we conducted a systematic review (following the PRISMA guidelines) to evaluate the effectiveness of various resistance exercise interventions on functional capacity in individuals with PFPS. This study was registered in PROSPERO (CRD42024616514). Seven databases were consulted (PubMed, Web of Science, CINAHL, SPORTDiscus, PEDro, Embase, and Scopus) from inception to April 2025. Eligible studies were randomized controlled trials (RCTs) comparing resistance exercise interventions with each other or with control, using the Anterior Knee Pain Scale (AKPS) as the primary outcome measure. Risk of bias was assessed using the Cochrane Risk of Bias Tool. Out of 1455 identified articles, 20 met the inclusion criteria. Three studies showed concerns in the randomization process, six in protocol deviations, one in missing outcome data, six in outcome measurement, and all studies exhibited high risk of bias in the selection of reported results. According to the Grading of Recommendations, Assessment, Development, and Evaluation (GRADE) framework, the overall quality of evidence ranged from low to moderate. Included studies, published between 2000 and 2024, involved 1230 participants aged from 14 to 43 years, all diagnosed with PFPS for at least four weeks. Ten studies included only women, nine included both sexes, and one included only men. Regarding activity level, 11 studies did not report it, while four focused on sedentary individuals and five on active populations. Interventions lasted from 4 to 12 weeks, with frequencies varying from once daily to five times a week. Interventions included resistance training alone (8 studies) or combined with proprioceptive training (2), core strengthening (3), foot exercises (2), with one study comparing these two combinations, or therapeutic education (4). From this systematic review we can conclude that, while there is a well-established consensus supporting the efficacy of resistance exercise-based interventions for improving functional capacity in individuals with PFPS, further high-quality research is needed to determine the most effective exercise modalities.

**Keywords**: resistance exercise; patellofemoral pain; functional capacity; anterior knee pain scale

**Funding**: This study was supported by the University of Oviedo (PAPI-24-GR-AstuRES).

Sports 2023, 11, x FOR PEER REVIEW 2 of 2

**References**

1. Crossley KM, Stefanik JJ, Selfe J, Collins NJ, Davis IS, Powers CM, et al. 2016 Patellofemoral pain consensus statement from the 4th International Patellofemoral Pain Research Retreat, Manchester. Part 1: Terminology, definitions, clinical examination, natural history, patellofemoral osteoarthritis and patient-reported outcome measures. Br J Sports Med. 2016 Jul;50(14):839–843.
2. Rathleff MS, Rathleff CR, Olesen JL, Rasmussen S, Roos EM. Is knee pain during adolescence a self-limiting condition? Prognosis of patellofemoral pain and other types of knee pain. Am J Sports Med. 2016 May;44(5):1165–1171.
3. Willy RW, Hoglund LT, Barton CJ, Bolgla LA, Scalzitti DA, Logerstedt DS, et al. Patellofemoral pain. J Orthop Sports Phys Ther. 2019 Sep;49(9):CPG1–CPG95.

**Disclaimer/Publisher’s Note:** The statements, opinions and data contained in all publications are solely those of the individual author(s) and contributor(s) and not of MDPI and/or the editor(s). MDPI and/or the editor(s) disclaim responsibility for any injury to people or property resulting from any ideas, methods, instructions or products referred to in the content.

### 2.16. Comparative Effectiveness of Resistance Exercise Protocols for Patellofemoral Pain: A Systematic Review

Pedro Diez-Solorzano, Toni Bailen-Garcia, Miguel Ángel Rodriguez, Beatriz Sanchez-Martinez and Maria Medina-Sanchez y Hugo Olmedillas *

Department of Functional Biology, University of Oviedo, Oviedo, Spain

Asturian Research Group in Performance, Readaptation, Training and Health (AstuRES), University of Oviedo, Oviedo, Spain

Department of Educational Sciences, University of Oviedo, Spain

Department of Surgery, University of Oviedo, Spain

*  Correspondence: olmedillashugo@uniovi.es

**Abstract**

Patellofemoral pain syndrome (PFPS) is a prevalent musculoskeletal disorder characterized by diffuse pain around the patella, aggravated by activities involving axial loading and knee movement such as walking, running, or stair climbing. This condition significantly impairs physical and athletic performance, diminishing patients’ quality of life. Although resistance exercise is widely acknowledged as the most effective therapeutic strategy, there is insufficient evidence to determine the most appropriate exercise modalities. This gap limits the development of standardized, evidence-based clinical guidelines.

To address this issue, we conducted a systematic review following PRISMA guidelines to assess the effectiveness of various resistance exercise interventions on perceived pain in individuals with PFPS. This study was registered in PROSPERO (CRD42024616514). Seven databases (PubMed, Web of Science, CINAHL, SPORTDiscus, PEDro, Embase, and Scopus) were consulted from inception to April 2025. Eligible studies were randomized controlled trials comparing resistance exercise interventions with each other or with control groups, using the Visual Analogue Scale (VAS), Numeric Rating Scale (NRS), or Verbal Rating Scale (VRS) as primary outcomes. Risk of bias was assessed using the Cochrane Risk of Bias Tool. From 1455 identified records, 34 studies met the inclusion criteria. Seven studies showed concerns in the randomization process, nine in deviations from intended interventions, one in missing outcome data, and thirteen in outcome measurement. All studies exhibited high risk of bias in selective outcome reporting. According to the GRADE framework, the overall quality of evidence ranged from low to moderate. The included studies (published between 2000 and 2025) involved 1847 participants aged 13.4 ± 9.7 to 43.9 ± 9.8 years, all diagnosed with PFPS for at least four weeks. Sixteen studies included only women, sixteen included both sexes, and two included only men. Regarding activity level, eighteen studies did not report it; six focused on sedentary individuals, and ten on active populations. Intervention duration ranged from 3 to 12 weeks, with frequencies varying from once daily to five times per week. Interventions included resistance training alone or combined with proprioception, core strengthening, foot exercises, therapeutic education, and gait retraining. From this systematic review, we conclude that although strong consensus supports the efficacy of resistance exercise–based interventions for improving perceived pain in people with PFPS, further high-quality research is required to determine the most effective exercise modalities.

**Keywords:** Resistance exercise; patellofemoral pain syndrome; pain; visual analogue scale; numeric rating scale; verbal rating scale

**Funding:** This study was supported by the University of Oviedo (PAPI-24-GR-AstuRES).

**References**

1. Crossley K. M., Stefanik J. J., Selfe J., Collins N. J., Davis I. S., Powers C. M., et al. 2016 Patellofemoral pain consensus statement from the 4th International Patellofemoral Pain Research Retreat, Manchester. Part 1: Terminology, definitions, clinical examination, natural history, patellofemoral osteoarthritis and patient-reported outcome measures. British Journal of Sports Medicine, 2016; 50(14): 839–843.
2. Rathleff M. S., Rathleff C. R., Olesen J. L., Rasmussen S., Roos E. M. Is knee pain during adolescence a self-limiting condition? Prognosis of patellofemoral pain and other types of knee pain. American Journal of Sports Medicine, 2016; 44(5): 1165–1171.
3. Willy R. W., Hoglund L. T., Barton C. J., Bolgla L. A., Scalzitti D. A., Logerstedt D. S., et al. Patellofemoral pain. Journal of Orthopaedic & Sports Physical Therapy, 2019; 49(9): CPG1–CPG95.

### 2.17. Safety of Heavy Resistance Exercise During Pregnancy and Postpartum: A Controlled, Prospective Cohort Study

Therese Fostervold Mathisen ^1,^*, Aliaksandr Hubin ^2^, Margo Mountjoy ^3^ and Jorunn Sundgot-Borgen ^4^

^1^  Faculty of Health, Welfare and Organization, Østfold University College, Fredrikstad, Norway
^2^  Norwegian University of Life Sciences, Ås, Norway
^3^  Department of Family Medicine, McMaster University, Hamilton, ON, Canada
^4^  Department of Sport Medicine, Norwegian School of Sport Sciences, Oslo, Norway
*  Correspondence: therese.f.mathisen@hiof.no; Tel.: +4795752818

**Abstract**

Physical activity is recommended for women during pregnancy, as it offers numerous mental, psychosocial, and physical health benefits for the pregnant woman, and positive effects for the fetus [1,2]. A common thread in national guidelines is the suggestion of engaging in aerobic and strengthening exercises at a low to moderate intensity, and strengthening the pelvic floor muscles, to realize these listed health benefits [1]. Notably, there is a scarcity of guidelines regarding engaging in heavy resistance exercise during pregnancy and the postpartum period. Consequently, many female power athletes find themselves lacking adapted guidelines for their exercise routines and instead encounter warnings and discouragement from both professionals and non-professionals [3]. Hence, the aim of this study is to address the knowledge gap on the influence of heavy free-weight resistance exercise on the health of the pregnant athlete and infant. Design: This is a prospective, controlled cohort study with two groups of pregnant women; those engaging in mainly heavy free-weight resistance exercise (REX, n = 33) and those regularly physically active (REF, n = 20). Data originates from eight questionnaires answered prospectively throughout pregnancy and 12 months postpartum. Variables cover exercise routines, pregnancy symptoms and Complications, fetal growth, delivery, and mother and infant’s health. Results: Bayesian logistic regression with random and fixed effects identified group membership as a significant predictor of proteinuria, with REX having higher exposure (posterior inclusion probability [PIP] = 1.00; β1 = 197.07, 95% Q2 CrI [163.05, 231.08]). Further, Bayesian logistic regression with random and fixed effects confirmed a higher risk of urinary incontinence in the REF group during pregnancy (PIP = 0.98; β1 = −1.17, 95% CrI [−1.89, −0.46]). Gas incontinence was also found more common in the REF group during pregnancy (PIP = 1.00; β1 = −7.62, 95% CrI [−8.68, −6.57]) and postpartum (PIP = 0.91; β1 = −49.39, 95% CrI [−53.57, −45.43]). There were no significant differences in the postpartum period for fecal and urinary incontinence, injuries, infant health outcomes (all PIP < 0.01), or need for assisted delivery (PIP = 0.33). Conclusion: Female athletes experienced in heavy resistance exercise, who continue training during pregnancy and postpartum, do not face increased pregnancy complications. These findings provide evidence supporting the safety of such exercise. Yet we highlight the need for a confirmatory study under full clinical supervision.

**Keywords:** female athlete; maternal health; postnatal; resistance training; incontinence; delivery; proteinuria

**Funding:** This research received no external funding.

**References**

1. Hayman, M., et al., Public health guidelines for physical activity during pregnancy from around the world: a scoping review. British Journal of Sports Medicine, 2023. **57**(14): p. 940–947.
2. Perales, M., et al., Benefits of aerobic or resistance training during pregnancy on maternal health and perinatal outcomes: A systematic review. Early Hum Dev, 2016. **94**: p. 43–8.
3. Weaving, C., Prenatal Paranoia: An Analysis of the Bumpy Landscape for the Pregnant Athlete. Sport, Ethics and Philosophy, 2020. **14**(2): p. 176–191.

**Disclaimer/Publisher’s Note:** The statements, opinions and data contained in all publications are solely those of the individual author(s) and contributor(s) and not of MDPI and/or the editor(s). MDPI and/or the editor(s) disclaim responsibility for any injury to people or property resulting from any ideas, methods, instructions or products referred to in the content.

### 2.18. Age-Related Divergence Between Linear and Hybrid Force–Velocity Models in Leg Press Exercise

**David Garcia-Albin ^1,2,3,6^, Mikel Garcia-Aguirre ^1,2,3^, Hector Gutierrez Reguero ^1,2,3^, Ivan Baltasar-Fernandez ^1,2,3,4^, Ignacio Ara ^1,2,3^, Francisco J. García García ^2,5,^, Luis M. Alegre ^1,2,3^ and Julian Alcazar ^1,2,3^**

^1^  GENUD Toledo Research Group, Faculty of Sport Sciences, University of Castilla-La Mancha, Toledo, Spain
^2^  Centro de Investigación Biomédica en Red Fragilidad y Envejecimiento Saludable (CIBERFES), Instituto de Salud Carlos III, Madrid, Spain
^3^  Grupo Mixto de Fragilidad y Envejecimiento Exitoso UCLM-SESCAM (TEC2022-007), Instituto de Investigación Sanitaria de Castilla-La Mancha (IDISCAM), Junta de Comunidades de Castilla-La Mancha (JCCM), Toledo, Spain
^4^  Faculty of Health Sciences, University of Castilla-La Mancha, Talavera de la Reina, Spain
^5^  Geriatrics Department, Hospital Universitario de Toledo, Toledo, Spain
^6^  Fundación del Hospital Nacional de Parapléjicos para la Investigación y la Integración
*  Correspondence: Julian.alcazar@uclm.es

**Abstract**

The force-velocity (F-V) profile is a critical determinant of neuromuscular performance. Although this relationship was originally described as hyperbolic by Hill et al. (1938), the linear model has been predominantly used in recent years due to its simplicity and practical applicability. However, this approach may potentially overlook significant mechanisms that underlie the hyperbolic nature of the F–v relationship, such as those described by Alcazar et al. (2019;2022) (muscle fiber type, muscle architecture, or tendon compliance), which could help to differentiate phenotypes associated with aging or lifestyle. Consequently, this study aimed to examine how the divergence between linear and hybrid F–V models—quantified as the difference in area under the curve (ΔAUC), difference in maximal velocity (Δv_0_), and divergence point (k)—varies among different phenotypes.

A total of 254 participants were divided into four groups: 28 young adults (25.6 ± 2.4 years; 15 men, 13 women), 166 middle-aged adults (54.6 ± 1.4 years; 71 men, 95 women), 31 robust older adults (71.6 ± 5.8 years; 13 men, 18 women), and 29 master athletes (69.0 ± 3.8 years; 24 men, 5 women). Each participant performed a F–v profile using a leg-press machine. Each F-V profile included at least three trials performed above 45% of the individual’s F_0_ and one trial below this threshold. Both the linear and hybrid models were applied, and ΔAUC, Δv_0_, and the divergence point k were calculated. A linear mixed-effects model was used to assess differences between groups, with group and sex included as a fixed effect.

A significant group effect was found for ΔAUC (F = 9.91; *p* < 0.001). Post hoc tests showed greater ΔAUC in young adults compared to middle-aged adults (mean difference [95%CI] = 48.54 [27.76, 69.33]; *p* < 0.001), robust older adults (68.48 [42.16, 94.80]; *p* < 0.001), and master athletes (59.39 [28.47, 90.31]; *p* < 0.001). Middle-aged adults also had higher ΔAUC than robust older adults (19.94 [0.48, 39.39]; *p* = 0.045), however, no significant differences were found between middle-aged adults and master athletes. No group differences were observed for Δv_0_ (F = 0.94; *p* = 0.422) or k (F = 2.10; *p* = 0.101).

These findings suggest that the divergence between linear and hybrid F–V models may vary with age and training status, with younger individuals exhibiting greater ΔAUC. Notably, lifelong exercise, as observed in master athletes, may help attenuate these age-related differences. The F–v profile may provide valuable insights into underlying physiological characteristics and their evolution across the lifespan; however, a more accurate assessment of high-velocity regions is warranted to enhance model sensitivity.

**Keywords:** Force-velocity profile; neuromuscular performance; ageing

**Funding:** This work was supported by the Biomedical Research Networking Centre on Frailty and Healthy Ageing (CIBERFES) and FEDER funds from the European Union (Grant No. CB16/10/00477, CB16/10/00456). It was further funded by grants from the Government of Castilla-La Mancha (Grant No. PI2010/020), the Ministry of Health of Castilla-La Mancha (Institute of Health Sciences) (Grant No. 03031-00), the Spanish Ministry of Economy “Ministerio de Economía y Competitividad” (Instituto de Salud Carlos III) (Grant No. PI10/01532, PI031558. DGA was supported by IDISCAM, Programa INVESTIGO, Ref: 2024_INVESTIGO_05. Finally, the co-authors would like to thank the Strength and Conditioning Society (SCS) for improving knowledge of exercise for older adults and for its ongoing dissemination and promotion of research in this area.

**References**

1. Alcazar et al. On the Shape of the Force-Velocity Relationship in Skeletal Muscles: The Linear, the Hyperbolic, and the Double-Hyperbolic. Front. Physiol., (**2019**), *10*.
2. Alcazar et al. A novel equation that incorporates the linear and hyperbolic nature of the force-velocity relationship in lower and upper limb exercises. *European Journal of Applied Physiology **(2022)** 122:2305–2313*.
3. Hill AV. The heat of shortening and the dynamic constants of muscle. Proceedings of the Royal Society B: Biological Sciences. (**1938)**;126(843):136–195.

**Disclaimer/Publisher’s Note:** The statements, opinions and data contained in all publications are solely those of the individual author(s) and contributor(s) and not of MDPI and/or the editor(s). MDPI and/or the editor(s) disclaim responsibility for any injury to people or property resulting from any ideas, methods, instructions or products referred to in the content.

### 2.19. Daily Fluctuations in Brain-Derived Neurotrophic Factor and Cognitive Performance: Effects of Acute Physical Activity

**Jacopo Givralli ^1^, Davide Charrier ^1^, Giuseppe Cerullo ^1^, Alessandro Sampieri ^1^, Gioi Spinello ^1^, Valeria Stircu ^1^, Marta Canato ^1^, Tatiana Moro ^1^ and Antonio Paoli ^1^**

^1^  Department of Biomedical Sciences, University of Padua

**Abstract**

Cognitive functions are fundamental to everyday life, underpinning the mental processes required to perform any activity. Recent studies have shown that improved cognitive performance is associated with elevated levels of brain-derived neurotrophic factor (BDNF), which can be increased through an acute bout of physical activity. However, daily variations of BDNF serum and plasma remain poorly characterised and are not fully understood, and their correlation with daily variations in cognitive performance has not been previously explored. This study examines the correlation between daily BDNF fluctuations and cognitive performance, with the aim of evaluating exercise as a targeted intervention to boost cognition during the workday. Furthermore, it assesses whether a single session of exercise can elevate BDNF levels and lead to measurable cognitive improvements. Fourteen healthy, physically active adults (7 males and 7 females) participated in the study. The intervention was conducted over two non-consecutive days, spaced two weeks apart. Participants’ lifestyle habits were standardised during the 24 h preceding each intervention day. Blood samples were collected at five time points throughout the workday: 09:00 (T1), 11:00 (T2), 13:00 (T3), 15:00 (T4), and 17:00 (T5), to assess potential diurnal variations in BDNF. Cognitive performance was evaluated using the Psychomotor Vigilance Test (PVT), the Flanker task, and the Digit Span Backwards test at T1, T3, and T5. On the second intervention day, the same protocol was followed, with the addition of a 15-min single bout of high-intensity physical activity executed at a specific time during the day. Preliminary analysis showed a decline, from T1 to T5, in the ratio between memory span and total time of the Digit Span Backwards, suggesting a small decrease in the working memory. In the Flanker task, a slight decrease in mean response time from T1 to T5 suggests a progressive improvement in response speed. However, the conflict cost shows a slight increase from T1 to T5, which might reflect a small decrease in the ability to resolve cognitive interference. In the PVT, both mean and median RT reaction time showed a decreasing trend from T1 to T5 across all delay values, especially noticeable at higher delays, suggesting improved sustained attention and faster responses over time. Preliminary findings suggest distinct circadian patterns across different cognitive functions, indicating a high intra-subject variability. However, comprehensive analyses, including BDNF data and their potential correlations with cognitive outcomes and exercise intervention, are currently ongoing and will be completed soon.

**Keywords:** Brain-derived neurotrophic factor; Cognition; Circadian Rhythms; Exercise; Physical Activity; Brain

**Funding:** This research received no external funding.

**Disclaimer/Publisher’s Note:** The statements, opinions and data contained in all publications are solely those of the individual author(s) and contributor(s) and not of MDPI and/or the editor(s). MDPI and/or the editor(s) disclaim responsibility for any injury to people or property resulting from any ideas, methods, instructions or products referred to in the content.

### 2.20. Acute Hemodynamic Responses in Persons with Chronic Heart Failure During Resistance Training with Different Intensities

Terje Gjøvaag ^1,^*, Rune Wårviken ^1^, Sander Østbye ^1^, Hilde Worren ^1^, Benedicte Christensen ^1^ and Birgitta Blakstad Nilsson ^1^

^1^  Dep. Rehabilitation Science and Health Technology, Oslo Metropolitan University Oslo, Norway
*  Correspondence: terje.gjovaag@oslomet.no

Resistance training (RT) is recommended in rehabilitation of cardiovascular diseases. However, what RT intensity should be selected, is debated. For persons with chronic heart failure (CHF), European guidelines recommend medium-intensity RT with 40–60% of 1 RM and 12–15 repetitions [1]. Also, there is a widely held belief that high-intensity RT (HI-RT), lead to greater cardiovascular demands than medium-intensity RT (MI-RT) [2]. However, when healthy adults and adults with coronary artery disease [3,4] perform HI-RT and MI-RT, using the same exercise protocol as the current study, acute blood pressure increases significantly more following MI-RT. The European Society of Cardiology has questioned whether recommendations for RT in cardiac rehabilitation need reconsideration.

Consequently, the present study investigated the effect of HI-RT and MI-RT on systolic blood-pressure (SBP) and cardiovascular responses in persons with CHF. To investigate this, ten persons, mean ± sd age 63.9 ± 7.4 and left ventricular-ejection fraction (%) of 36.3 ± 6.2, performed three series (S1, S2 and S3) of seated knee-extensions (no breath-holding during exercise) with a MI-RT (49.8 ± 3.3% 1 RM, 15 repetitions) or HI-RT protocol (84.7 ± 4.3% 1 RM, 4 repetitions) in a randomized cross-over design with one week between protocols. Non-invasive, beat-by-beat SBP (mmHg) and cardiac output (CO; L min^−1^) were measured with a Finapres NOVA (FMS, The Netherlands) and PhysioFlow PF07 Enduro, respectively.

SBP resting values during HI-RT and MI-RT were similar (120 ± 10 and 118 ± 10 mmHg, respectively). Compared to Rest, S1, S2 and S3 SBP increased significantly during MI-RT (all comparisons; *p* = 0.001) and likewise during HI-RT (all comparisons; *p* < 0.05). Notably, S1, S2, and S3 SBP values were significantly lower during HI-RT compared to MI-RT, S1; 139 ± 17 vs. 162 ± 24 (*p* = 0.02), S2; 142 ± 20 vs. 168 ± 31 (*p* = 0.03) and S3; 141 ± 17 vs. 185 ± 42 mmHg (*p* = 0.002).

CO resting values during HI-RT and MI-RT were similar (5.3 ± 1.3 and 5.3 ± 1.0 L min^−1^, respectively). Compared to Rest, S1, S2 and S3 CO increased significantly during MI-RT (all comparisons; *p* < 0.01) and likewise during HI-RT (all comparisons; *p* < 0.05). However, S1, S2, and S3 CO values were similar during HI-RT and MI-RT, S1; 10.1 ± 3.4 vs. 11.9 ± 5.4 (*p* = 0.19), S2; 9.7 ± 3.3 vs. 11.5 ± 5.0 (*p* = 0.18), and S3; 9.7 ± 3.6 vs. 10.3 ± 2.3 (*p* = 0.49) L min^−1^.

In conclusion, HI-RT results in significantly lower SBP but similar CO responses compared to MI-RT. The CO response is blunted, but the SBP response in accordance with previous studies [3,4]. Hence, current international guidelines are challenged and there is need of further research to optimize resistance exercise for persons living with CHF.

**Keywords:** Resistance training, heart failure, blood-pressure, cardiac rehabilitation, guidelines

**Funding:** This research received no external funding.

**References**

1. Ambrosetti M, Abreu A, Corra U et al. Secondary prevention through comprehensive cardiovascular rehabilitation: From knowledge to implementation. 2020 update. A position paper from the Secondary Prevention and Rehabilitation Section of the European Association of Preventive Cardiology. Eur J Prev Cardiol, 2020: 460–495.
2. Hansen D, Abreu A, Doherty P, Voller H. Dynamic strength training intensity in cardiovascular rehabilitation: is it time to reconsider clinical practice? A systematic review. Eur J Prevent Cardiol 26(14): 1483–1492.
3. Gjøvaag T, Hjelmeland AK, Oygard JB, et al. Acute hemodynamic and cardiovascular responses following resistance exercise to voluntary exhaustion. Effects of different loadings and exercise durations. J Sports Med Phys Fitness 2016; 56: 616–623.
4. Gjøvaag T, Mirtaheri P, Simon K, et al. Hemodynamic responses to resistance exercise in patients with coronary artery disease. Med Sci Sports Exerc 2016; 48: 581–588.

**Disclaimer/Publisher’s Note:** The statements, opinions and data contained in all publications are solely those of the individual author(s) and contributor(s) and not of MDPI and/or the editor(s). MDPI and/or the editor(s) disclaim responsibility for any injury to people or property resulting from any ideas, methods, instructions or products referred to in the content.

### 2.21. Injury Prediction, Monitoring and Prevention Within Gymnastics: A Scoping Review

Islay Grant, Paul Swinton, Kay Cooper and Andy Hall ^1,^*

^1^  School of Pharmacy, Applied Sciences and Public Health, Robert Gordon University, Aberdeen, AB10 7QG, United Kingdom
^2^  School of Health Sciences, Robert Gordon University, Aberdeen, AB10 7QG, United Kingdom
^3^  The Scottish Centre for Evidenced-Based, Multi-professional Practice: A Joanna Briggs Institute Centre of Excellence, School of Health Sciences, Robert Gordon University, Aberdeen, AB10 7QG, United Kingdom
*  Correspondence: a.hall9@rgu.ac.uk; Tel.: +44 (0) 1224 263367

**Abstract**

Within gymnastics, frequent large loads and decelerations experienced by athletes’ underdeveloped skeletons, and the repetitive nature of movements create substantial injury risks. Research has explored applying prevention techniques to reduce injury risk and the impact of injury, including screening, monitoring, and targeted interventions. To date, no review has comprehensively mapped injury prevention techniques in gymnastics, encompassing each of these strategy types. Therefore, the aims of this scoping review were to identify and collate existing research investigating injury prevention in gymnastics and provide a detailed map of: (1) injury surveillance methods used, including screening tools to predict injuries and monitoring tools assessing current injury risk; and (2) interventions aimed at reducing injury risk. A scoping review was conducted following JBI guidance in line with an a-priori protocol. A three-step search strategy was employed to identify relevant articles, including a preliminary search, a comprehensive search, and manual screening of reference lists. Twelve electronic databases were searched from inception to August 2024. The inclusion criteria were; human participants of any gender, aged 8–65 years, involved in any gymnastics discipline across any competitive level; and studies aiming to predict, monitor or assess injury occurrence or risk. Studies conducted in any setting were eligible. Studies were excluded if they did not report primary data, they focused on non-gymnastics-related injuries, or were reviews, commentaries, or other non-original research. Two independent reviewers completed title and abstract, and full-text screening to determine inclusion. The same reviewers extracted data using a bespoke data collection tool to record information on: study design, participant demographics, injury information, and study methods. The search yielded a total of 2855 articles, with 91 included (41 prediction, 14 monitoring, and 36 intervention studies). Studies were comprised of cohort and cross-sectional designs, with prospective cohort the most common approach. The most common population was female artistic gymnasts, at a National competitive level. Main analysis types utilised for injury prediction were correlations, logistic regression, and group differences. Injury monitoring approaches were generally applied across 4–12 month periods and monitored training and competition loads. Most interventions were focussed on technique evaluation or implementing a specific intervention. Limited investigations of validity or reliability were included in study methods. Based on the map of the research created, there is a need to create standardised prevention tools, focusing on the validity and reliability and encompassing each component of injury prevention, as well as aim to create more accurate gymnastics-specific injury prediction models. 

**Keywords:** gymnastics; injury prevention; injury risk; injury monitoring; screening tools; injury prediction

**Funding:** This research was funded by the Robert Gordon University, Interdisciplinary Studentship.

**References**

1. Armstrong, R.; Relph, N. Screening Tools as a Predictor of Injury in Gymnastics: A Systematic Literature Review. *Sports Medicine—Open* **2021**, *7*, 1–18.
2. Bradshaw, E. J.; Hume, P. A. Biomechanical Approaches to Identify and Quantify Injury Mechanisms and Risk Factors in Women’s Artistic Gymnastics. *Sports Biomechanics* **2012**, *11*, 324–341.
3. Hammoudi, S. N.; Mkaouer, B.; Hammoudi, S. R.; Wali, S. M.; Nassib, S. Prediction of Gymnastics Physical Profile Through an International Program Evaluation in Women Artistic Gymnastics. *J. Strength Cond. Res.* **2020**, *34*, 577–586.
4. Thomas, R. E.; Thomas, B. C. A Systematic Review of Injuries in Gymnasts. *Phys Sportsmed* **2019**, *47,* 96–121.

**Disclaimer/Publisher’s Note:** The statements, opinions and data contained in all publications are solely those of the individual author(s) and contributor(s) and not of MDPI and/or the editor(s). MDPI and/or the editor(s) disclaim responsibility for any injury to people or property resulting from any ideas, methods, instructions or products referred to in the content.

### 2.22. Changes in Neuromuscular, Inflammatory, Wellness, and Speed-Related Status Throughout a Congested Professional Soccer Championship

Rafael Grazioli, Júlia Barreira, Jesús Ángel Cubero Martínez, Andre Luis Aroni, Fernando Maestri, Filipe Veeck, Gabriel dos Santos Oliveira, Tiago Tonon, Laura Alberti Zandavalli*, Ronei Silveira Pinto, Rodrigo Leitão, Eduardo Lusa Cadore and Irineu Loturco

Exercise Research Laboratory, School of Physical Education, Physiotherapy, and Dance, Universidade Federal do Rio Grande do Sul, Porto Alegre, RS, Brazil

Department of Physical Education, São Francisco University, Bragança Paulista, SP, Brazil

Department of Physiology and Athletic Performance, Criciúma Sport Club, Criciúma, SC, Brazil Faculdade de Educação Física, Universidade Estadual de Campinas, Campinas, Brazil Department of Health and Performance, Guarani Football Club, Campinas, SP, Brazil

Department of Human Movement Sciences, Federal University of São Paulo, São Paulo, Brazil NAR—Nucleus of High Performance in Sport, São Paulo, Brazil

UCAM Research Center for High Performance Sport, Universidad Católica de Murcia, Murcia, Spain, Facultad de Deporte, Universidad Católica de Murcia, Murcia, Spain

*  Correspondence: laurazandavalli@outlook.com

**Abstract**

The current landscape of high-performance soccer presents numerous challenges due to congested match schedules, with players often competing in 60–80 matches per season and recovering for fewer than five days between games. These conditions require complex physical capabilities, while accumulated fatigue may impair performance and increase injury risk.

This study aimed to investigate neuromuscular, inflammatory, wellness, and high-intensity match-play responses in professional soccer players throughout a congested championship period. Additionally, it examined associations between these markers and playing time, accumulated weekly training load, and 48-h pre-match load, along with comparisons between the first and second halves of the championship.

Twenty-two male professional players (28.1 ± 4.2 years) were monitored across 12 official matches within 52 days. Measurements prior to each match included countermovement jump (CMJ), C-reactive protein (CRP), and rate of perceived recovery (RPR). In-match accelerations and sprints per minute were assessed via GPS.

Data were analyzed using One-Way ANOVA, Pearson’s correlation, and independent-samples *t*-tests, with Cohen’s d for effect size.

Results revealed no significant longitudinal changes in CMJ, CRP, RPR, or high-intensity match metrics across the 12 matches, indicating physiological stability despite the dense schedule. No associations were identified between playing time and any monitored variable. However, sprint load showed a strong positive correlation with match-day sprint performance (r = 0.83) and a moderate negative correlation with RPR when measured 48 h pre-match (r = −0.38), suggesting that short-term high-intensity exposure may hinder subjective recovery. A significant decline in acceleration rate was detected in the second half of the championship (*p* < 0.001; d = 0.57; Δ% = −6.5%), potentially reflecting accumulated fatigue.

Overall, professional soccer players maintained physiological and performance stability across a highly congested schedule, likely due to effective load and recovery management. These findings support the importance of integrated monitoring to guide training decisions and mitigate fatigue.

**Keywords:** Performance; workload; soccer

**Funding:** This research received no external funding.

**References**

1. Julian R., Page R. M., Harper L. D. The Effect of Fixture Congestion on Performance During Professional Male Soccer Match-Play: A Systematic Critical Review with Meta-Analysis. Sports Medicine, 2021; 51: 255–273.
2. Silva J. R., Rumpf M. C., Hertzog M., Castagna C., Farooq A., Girard O., Hader K. Acute and Residual Soccer Match-Related Fatigue: A Systematic Review and Meta-Analysis. Sports Medicine, 2018; 48: 539–583.

### 2.23. Sex-Specific Acute Effects of High-Intensity Interval and Moderate-Intensity Continuous Training on Appetite Perceptions and Their Relationship with Microbiota Profile in Adults with Type 2 Diabetes

Andrea González-Mariscal ^1,^*, Alberto Marín-Galindo ^1^, Adrián Montes-de-Oca-García ^1^, Juan Corral-Pérez ^1^, María Rebollo-Ramos ^1^, Manuel Costilla ^1^, Laura Ávila-Cabeza-de-Vaca ^1^, Julio Plaza-Diaz ^2,3^, Cristina Casals ^1^ and Jesus G. Ponce-Gonzalez ^1^

^1^  ExPhy Research Group, Department of Physical Education, Instituto de Investigación e Innovación Biomédica de Cádiz (INiBICA), Universidad de Cádiz, Cádiz, Spain.; andrea.gonzalez@uca.es; alberto.marin@uca.es; adrian.montesdeoca@uca.es; juan.corral@uca.es; maria.rebollo@uca.es; manueljesus.costilla@uca.es; laura.avila@uca.es; cristina.casals@uca.es; jesusgustavo.ponce@uca.es
^2^  ANUT-DSM (Alimentaciò, Nutrició Desenvolupament i Salut Mental), Departament de Bioquímica i Biotecnologia, Universitat Rovira i Virgili, 43201 Reus, Spain, julioramon.plaza@urv.cat
^3^  School of Health Sciences, Universidad Internacional de La Rioja, Avenida de la Paz 137, 26006 Logroño, Spain
*  Correspondence: andrea.gonzalez@uca.es

**Abstract**

In type 2 diabetes (T2DM), high-intensity interval training (HIIT) and moderate-intensity continuous training (MICT) may affect appetite differently, though findings are mixed (1,2), and the gut microbiota has emerged as a potential modulator of appetite, possibly interacting with exercise (3). Thus, this study compared the sex-specific acute effects of HIIT and MICT on appetite perceptions and explored their associations with gut microbiota in adults with T2DM. Seventy-nine participants (40 women) were randomly assigned to either HIIT protocol (n = 43; 10 × 1-min intervals at 90% peak power) or MICT protocol (n = 36; 50 min at moderate intensity). Subjective appetite was assessed before and immediately after exercise using visual analogue scales (VAS) for hunger, satiety, fullness, desire to eat, and cravings (sweet, salty, savoury, fatty). Two appetite indices were calculated: Index 1 = [hunger + (100 − fullness) + desire to eat]/3; Index 2 = (hunger + desire to eat) − (satiety + fullness). Fecal samples were collected at baseline and analysed via 16S rRNA sequencing to characterize gut microbial diversity and composition. Post-exercise, hunger (t = −2.609, *p* = 0.011) and Index 1 (t = −2.057, *p* = 0.043) increased. Women reported higher craving for fatty foods (t = −2.104; *p* = 0.039). No differences were found between HIIT and MICT overall, but significant time*sex interactions were observed for Index 1 (F = 4.46; η^2^p = 0.056; *p* = 0.038) and Index 2 (F = 4.19; η^2^p = 0.053; *p* = 0.044), with greater appetite increases in men. Microbiota diversity (Fisher Alpha (FA), Inverse Simpson (IS)) was negatively associated with post-exercise fullness and cravings for salty and fatty foods. FA was inversely related to post-exercise salty craving (r = −0.374, *p* < 0.001) and changes in fullness, salty, and fatty desire. Species richness negatively correlated with fat desire (rho = −0.322; *p* = 0.004) and changes in fullness. The Firmicutes/Bacteroidetes (F/B) ratio correlated with hunger increase (rho = 0.245; *p* = 0.030). Sex-stratified analyses revealed stronger associations in women: FA and IS were inversely related to fullness and salty desire; the F/B ratio was positively correlated with hunger and Index 2. In men, no significant associations were observed. In HIIT, FA and IS were negatively correlated with salty desire (r = −0.521; *p* < 0.01 and r = −0.320; *p* = 0.036); in MICT, FA was inversely associated with fullness (r = −0.468; *p* = 0.004) and fat desire (r = −0.405; *p* = 0.014). Species richness consistently correlated with lower post-exercise fat desire (rho = −0.432; *p* = 0.009) and greater fullness (rho = −0.512; *p* = 0.001). Pielou’s evenness correlated positively with baseline savoury and fat food desire (rho = 0.344; *p* = 0.024). These findings suggest that acute exercise increased appetite in T2DM, particularly in men. While HIIT and MICT exerted comparable effects on appetite, gut microbiota diversity appeared to attenuate post-exercise appetite, especially among women, indicating potential sex-specific exercise–microbiota–appetite interactions.

**Keywords**: Gut microbiome diversity, visual analogue scale, cravings, appetite indices, exercise metabolism, obesity

**Funding**: This research was funded by Ministry of Science and Innovation, grant number PID2019-110063RA-I00/AEI/10.13039/501100011033 (EDUGUTION project).

**References**

1. Poon, E.; Sun, F.; Chung, A.P.; Wong, S. Post-Exercise Appetite and Ad Libitum Energy Intake in Response to High-Intensity Interval Training versus Moderate- or Vigorous-Intensity Continuous Training among Physically Inactive Middle-Aged Adults. Nutrients 2018, 10, 1408.
2. Hu, M.; Nie, J.; Lei, O.-K.; Shi, Q.; Kong, Z. Acute Effect of High-Intensity Interval Training versus Moderate-Intensity Continuous Training on Appetite Perception: A Systematic Review and Meta-Analysis. Appetite 2022, 182, 106427.
3. Van de Wouw, M.; Schellekens, H.; Dinan, T.G.; Cryan, J.F. Microbiota-Gut-Brain Axis: Modulator of Host Metabolism and Appetite. J. Nutr. 2017, 147, 727–745.

**Disclaimer/Publisher’s Note:** The statements, opinions and data contained in all publications are solely those of the individual author(s) and contributor(s) and not of MDPI and/or the editor(s). MDPI and/or the editor(s) disclaim responsibility for any injury to people or property resulting from any ideas, methods, instructions or products referred to in the content.

### 2.24. Differential Impact of Mechanical and Endocrine Stimuli on Bone Metabolism Biomarkers: 24 H Acute Responses to Resistance and Endurance Exercise Across the Menstrual Cycle

Isabel Guisado-Cuadrado ^1,^*, Madi Bell ^2^, Nuria Romero-Parra ^1,3^, Panagiota Klentrou ^2^ and Ana B. Peinado ^1^

^1^  Department of Health and Human Performance, Faculty of Physical Activity and Sport Sciences, Universidad Politécnica de Madrid, Madrid, Spain. i.guisadoc@upm.es; anabpeinado@upm.es
^2^  Department of Kinesiology, Faculty of Applied Health Sciences, Brock University, Ontario, Canada. nklentrou@brocku.ca; mb14pf@brocku.ca
^3^  Department of Physical Therapy, Occupational Therapy, Rehabilitation and Physical Medicine, Faculty of Health Sciences, Universidad Rey Juan Carlos, Madrid, Spain. nuria.romero@urjc.es
*  Correspondence: i.guisadoc@upm.es

**Abstract**

The bone biomarker response to exercise varies significantly depending on the type and mechanical loading of the activity (Dolan et al. 2022). The lack of 24-h post-exercise biomarker data limits our understanding of the specific physiological mechanisms activated by different exercise modalities. In addition, estradiol’s critical role in bone metabolism (Khosla et al., 2012), the menstrual cycle offers a unique framework to study how fluctuations in sex hormone influence bone biomarker concentrations both at rest and in response to exercise. This study examined the acute effects of endurance and resistance exercise on circulating bone biomarkers (Dickkopf-1 (DKK-1), osteoprotegerin (OPG), parathyroid hormone (PTH), osteocalcin, sclerostin, and osteopontin) at rest and 24 h post-exercise in eumenorrheic females. Eight endurance-trained females (26.2 ± 3.6 years, 60.2 ± 4.5 kg, 24.5 ± 8.5% of fat) completed an interval running protocol (8 × 3-min treadmill runs at 85% of maximal speed). Nine resistance-trained females (28.2 ± 3.6 years, 57.1 ± 9.7 kg, 23.1 ± 8.8% of fat) performed an eccentric-based resistance exercise protocol (10 sets of 10 repetitions of 4-s parallel back squats at 60% 1 RM). The early follicular phase (EFP; days 2–5) and late follicular phase (LFP; 1–2 days before a positive LH urine test) were confirmed a posterior by serum sex hormone analysis. A significant effect of exercise modality was observed on sclerostin (endurance: 342.8 ± 113.5 pg·mL^−1^ vs. resistance: 623.9 ± 218.5 pg·mL^−1^; *p* = 0.013), with higher concentrations showed by females which performed the resistance protocol. A significant main effect of time was observed for osteocalcin (*p* = 0.047), with lower concentrations 24 h post-exercise (4308.5 ± 1793.7 pg·mL^−1^) vs. pre-exercise (4581.6 ± 2178.9 pg·mL^−1^) across both the endurance and resistance groups. A significant main effect of phase was observed for DKK-1 (*p* = 0.002), with higher concentrations in the LFP (1274.7 ± 263.7 pg·mL^−1^) vs. EFP (1146.5 ± 255.5 pg·mL^−1^). These findings emphasize the importance of accounting for hormonal fluctuations in female participants, as demonstrated by the phase-specific variation in DKK-1. The distinct sclerostin responses to endurance versus resistance exercise further suggest that exercise modality plays a key role in modulating bone metabolism. Continued research is needed to elucidate how mechanical and endocrine stimuli interact to regulate bone remodeling and to better understand the physiological mechanisms underlying exercise-induced bone adaptations.

**Keywords**: Bone metabolism; Resistance exercise; Endurance exercise; Menstrual cycle; Biomarkers

**Funding**: The IronFEMME Study took place with the financial support of the Ministerio de Economía y Competitividad, Convocatoria de ayudas I + D 2016, Plan Estatal de Investigación Científica y Técnica y de Innovación 2013–2016 [Grant DEP2016-75387-P] funded by MCIN/AEI/https://doi.org/10.13039/501100011033 and by “ERDF A way of making Europe”.

**References**

1. Dolan, E.; Dumas, A.; Keane, K.M.; Bestetti, G.; Freitas, L.H.M.; Gualano, B.; Kohrt, W.M.; Kelley, G.A.; Pereira, R.M.R.; Sale, C.; Swinton, P.A. The bone biomarker response to an acute bout of exercise: A systematic review with meta-analysis. Sports Med. 2022, 52, 1645–1674.
2. Khosla, S.; Oursler, M.J.; Monroe, D.G. Estrogen and the skeleton. Trends Endocrinol. Metab. 2012, 23, 576–581.

**Disclaimer/Publisher’s Note:** The statements, opinions and data contained in all publications are solely those of the individual author(s) and contributor(s) and not of MDPI and/or the editor(s). MDPI and/or the editor(s) disclaim responsibility for any injury to people or property resulting from any ideas, methods, instructions or products referred to in the content.

### 2.25. Acute Training Changes to Hip-Adduction Strength in Football

Maziar J. Hamad ^1,^*, Pedro E. Alcaraz ^1,2,3^, Kristian Thorborg ^4^, Steve Barrett ^5,6^ and Konstantinos Spyrou ^1,2,3^

^1^  UCAM Research Center for High Performance Sport, Catholic University of Murcia (UCAM), Murcia, Spain
^2^  Faculty of Sport Sciences, Catholic University of Murcia (UCAM), Murcia, Spain
^3^  Strength and Conditioning Society (SCS), Murcia, Spain
^4^  S Department of Sports, Department of Orthopedic Surgery, Orthopedic Research Center-Copenhagen (SORC-C), Amager-Hvidovre Hospital, Copenhagen University Hospital, Copenhagen, Denmark
^5^  Playermaker, London, United Kingdom
^6^  Global Institute of Sport, Manchester, United Kingdom
*  Correspondence: mjabar@ucam.edu

**Abstract**

The organization of training within a football microcycle is important to optimally prepare a team for an upcoming match (1)**.** Common weekly periodization strategies may show notable differences in imposed demands within the week (2). Low hip adduction strength is a modifiable groin injury risk factor and hip-adduction strength testing is used as a secondary prevention tool (3,4). This study examined the changes to hip-adduction strength, within the football microcycle. The microcycle included the following training sessions ((match day (MD)-4), (MD-2) and (MD-1)). Twelve male youth football players were recruited (age: 17.11 ± 0.52 years; body mass: 72.63 ± 5.53 kg; height: 1.78 ± 0.05 m) and had their maximal isometric hip-adduction strength (ADD_iso_) measured before and after each training session. Players wore foot-mounted inertial measurement units (Playermaker^TM^) during each training session to quantify locomotor activities. A linear mixed model was used to assess ADD_iso_ and percent changes, respectively. Statistical significance was set at ≤ 0.05. Results showed a significant difference in pre to post strength, *p* < 0.05, but not between pre-training strength, (MD-4_pre_, 3.04 Nmkg^−1^; MD-2_pre_, 3.09 Nmkg^−1^; MD-1_pre_, 3.04 Nmkg^−1^, *p* > 0.05). The group-level post-training percent changes to ADD_iso_ were, MD-4, −4.00 ± 7.29%, *p* = 0.083; MD-2, −3.76 ± 5.04%, *p* = 0.043; and MD-1, 7.43 ± 4.92%, *p* < 0.001. In summary, high intensity training sessions with high-speed running and change of direction actions, can result in reductions of hip-adduction strength, while sessions with less high intensity locomotor demands while emphasising shooting actions may provide a potentiating effect on ADD_iso_. This is important considering the relationship between hip-adduction strength and groin injury and pain.

**Keywords:** Groin, Isometric contraction, injury

**Funding:** This research received no external funding.

**References**

1. Los Arcos, A, Mendez-Villanueva, A, and Martínez-Santos, R. In-season training periodization of professional soccer players. *Biol Sport* 34: 149–155, 2017. Available from: https://www.termedia.pl/Journal/-78/pdf-28892-10?filename=7_00044_Article.pdf.
2. Martín-García, A, Gómez Díaz, A, Bradley, PS, Morera, F, and Casamichana, D. Quantification of a professional football team’s external load using a microcycle structure. *J Strength Cond Res* 32: 3511–3518, 2018. Available from: https://journals.lww.com/nsca-jscr/abstract/2018/12000/quantification_of_a_professional_football_team_s.26.aspxAuthor 1, A.; Author 2, B. *Book Title*, 3rd ed.; Publisher: Publisher Location, Country, 2008; pp. 154–196.
3. Bourne, MN, Williams, M, Jackson, J, et al. Preseason Hip/Groin Strength and HAGOS Scores Are Associated With Subsequent Injury in Professional Male Soccer Players. *J Orthop Sports Phys Ther* 50: 234–242, 2020. Available from: http://dx.doi.org/10.2519/jospt.2020.9022.
4. Wollin, M, Thorborg, K, Welvaert, M, and Pizzari, T. In-season monitoring of hip and groin strength, health and function in elite youth soccer: Implementing an early detection and management strategy over two consecutive seasons. *J Sci Med Sport* 21: 988–993, 2018.

**Disclaimer/Publisher’s Note:** The statements, opinions and data contained in all publications are solely those of the individual author(s) and contributor(s) and not of MDPI and/or the editor(s). MDPI and/or the editor(s) disclaim responsibility for any injury to people or property resulting from any ideas, methods, instructions or products referred to in the content.

### 2.26. Maximal Oxygen Uptake at Age 13 Predicts Future Levels in Endurance Athletes

Jostein Hallén ^1,^* and Hege Wilson Landgraff ^1^

^1^  Department of Physical Performance, Norwegian School of Sport Sciences, Oslo, Norway
*  Correspondence: josteinh@nih.no; Tel.: +4797039433

**Abstract**

The trainability of VO2max in adolescents remains a controversial topic, despite extensive research over several decades. This study aimed to investigate whether VO2max at the age of 13 can predict VO2max at the age of 18. Thirteen boys were recruited from cross-country ski clubs at age 12 and invited for follow-up assessments at ages 13, 15, and 18. VO2max was determined by an incremental running test to exhaustion on a treadmill, conducted at the same time each year post-season, with a consistent testing protocol across all four years. Participants engaged in both organized and self-organized training, progressively focusing on endurance, including high-intensity interval training 2 to 3 times per week. Each year, participants completed a questionnaire to document their sports participation and weekly training hours, and detailed interviews were conducted at ages 15 and 18 to gain further insight into their training content over the preceding year. Measurements of body height, weight, and fat-free mass (using InBody) were also recorded. The Pearson correlation coefficient between VO2max at ages 13 and 18 was calculated. In addition, the athletes were divided into two groups based on VO2max at age 18: Group 70 (n = 7) had VO2max above 70 mL·kg^−1^·min^−1^, while Group 60 had VO2max below this threshold. Differences between groups were tested using Student’s *t*-test, with values expressed as mean ± SD. The total training volume increased from an average of 7.0 ± 1.7 h per week at age 12 to 11.9 ± 3.2 h per week at age 18, with no significant differences between the two groups. There were no significant groups differences in height, weight, or fat-free mass at any time point. A strong correlation was found between VO2max at ages 13 and 18 (r = 0.822). In Group 70, VO2max values were 71.6 ± 2.4, 70.3 ± 2.8, 70.1 ± 5.3, and 74.0 ± 2.3 mL·kg^−1^·min^−1^ at ages 12, 13, 15, and 18, respectively. In contrast, Group 60 showed lower VO2max values at all time points: 64.1 ± 5.0, 62.0 ± 5.3, 64.0 ± 2.9, and 64.6 ± 4.0 mL·kg^−1^·min^−1^ (all *p* < 0.01). This study demonstrates that VO2max at age 13 can effectively predict VO2max at age 18 in male endurance athletes and suggests that the development of VO2max during these years is moderate despite the progressive increase in training volume.

**Keywords:** VO2max; adolescents; endurance training; puberty

**Funding:** This research received no external funding.

**Disclaimer/Publisher’s Note:** The statements, opinions and data contained in all publications are solely those of the individual author(s) and contributor(s) and not of MDPI and/or the editor(s). MDPI and/or the editor(s) disclaim responsibility for any injury to people or property resulting from any ideas, methods, instructions or products referred to in the content.

### 2.27. Contextual Talent Hotspots in Youth Soccer: A Case Study of Athletic Club De Bilbao

**Lander Hernández-Simal and Julio Calleja-Gonzalez**

**Abstract**

Birthplace and location of residence significantly affect opportunities for youth soccer development. This study aimed to identify “talent hotspots” that contribute the most players to the selection phases of Athletic Club de Bilbao—a professional soccer club in Spain with a unique regional recruitment system. We examined whether contextual variables such as population density, proximity to the academy, family income, and the number of clubs influenced player selection. Logistic regression on data from 1411 male players (aged 9–35) revealed Bizkaia, the largest province in the Basque Country, as a statistically significant hotspot (*p* < 0.05). The Odds Ratio analysis showed that municipalities with a youth male population density of 150–650 per km^2^ had significantly higher odds of player selection at multiple stages. These findings support previous research emphasizing the role of geography and community size in talent identification (Rossing et al., 2018; Côté et al., 2006). Moreover, the concept of “sporting development”—the environment where players engage with sport—emerged as a crucial factor (Hancock et al., 2022). Our results suggest that scouting should prioritize medium-density areas while ensuring equitable access to development resources across all regions (Hernández-Simal et al., 2024).

**Keywords**: Talent identification, youth soccer, birthplace effect, population density, contextual variables

**Funding:** This research received no external funding.

### 2.28. The Birthplace Effect in Soccer: A Systematic Review of Contextual Influences on Athlete Development

**Lander Hernández-Simal ^1^ and Julio Calleja-Gonzalez ^2^**

^1^  Faculty of Education and Sport, University of Deusto, 48007 Bilbao, Spain
^2^  Faculty of Education and Sport, University of Basque Country (UPV/EHU), 01007 Vitoria, Spain

**Abstract**

The “birthplace effect” refers to the influence of geographic and social contexts—such as community size, population density, and proximity to sport infrastructure—on athletes’ developmental opportunities and likelihood of reaching elite performance levels. This systematic review aimed to synthesize current evidence regarding the relationship between birthplace-related factors and sport participation or achievement across youth and elite contexts. Following PRISMA guidelines, 41 studies published between 2006 and 2023 were included. The majority of studies reported that athletes are more likely to reach elite levels if born in medium-sized communities with moderate population density and access to sport facilities. Notably, birthplace effects varied by sport type, cultural context, and athlete gender. Soccer, ice hockey, and basketball were the most frequently studied sports, with results consistently indicating that urban-rural dynamics shape athlete development pathways. The review also identified a lack of studies from non-Western countries and on female athletes, revealing a research gap. Findings support the theoretical framework of the Developmental Model of Sport Participation (Côté et al., 2007) and align with ecological models of talent development (Baker et al., 2020). Overall, the birthplace effect appears to be a robust contextual factor influencing athletic progression, with implications for talent identification and policy development in sport organizations.

**Keywords:** Birthplace effect, talent development, community size, ecological context, systematic review

**Funding:** This research received no external funding.

**References**

1. Orchard JW. Men at higher risk of groin injuries in elite team sports: A systematic review. Br J Sports Med. 2015;49:798–802.
2. Whittaker JL, Small C, Maffey L, Emery CA. Risk factors for groin injury in sport: an updated systematic review. Br J Sports Med. 2015;49:803–809.
3. Quintana-Cepedal M, De La Calle O, Medina-Sánchez M, Crespo I, Del Valle M, Olmedillas H. Characterising groin pain in rink hockey: Function and five-second squeeze in Spanish players. Phys Ther Sport. 2022;58:100–105.

### 2.29. Transitioning to 1500 m at LA2028: Impact of Duration on Energetic Contribution and Performance in Maximal Ergometer Rowing

Peter R. Higgins ^1,*^ and Mathijs J. Hofmijster ^1,2,3^

^1^  Department of Human Movement Sciences, Faculty of Behavioural and Movement Sciences, Vrije Universiteit Amsterdam; peterhiggins13@gmail.com (P.R.H.) m.hofmijster@vu.nl (M.J.H.)
^2^  Institute for Brain and Behaviour Amsterdam, The Netherlands
^3^  Amsterdam Movement Sciences—Sports, The Netherlands
*  Correspondence: peterhiggins13@gmail.com; Tel.: +353 89 708 2113 (P.R.H)

**Abstract**

For the first time in Olympic history, the standard 2000 m rowing distance will be shortened to 1500 m at the Los Angeles 2028 Games. Despite widespread implications, limited empirical evidence exists to quantify how this reduced duration alters performance determinants [1]. We investigated changes in energetic contribution and physiological characteristics associated with rowing performance when transitioning from the traditional 2000 m to the proposed 1500 m race distance. Specifically, we examine the relative contribution of anaerobic metabolism across durations simulating different boat classes. Nine highly trained male rowers (age: 23 ± 2.3 years; VO_2_Max: 62.6 ± 7.7 mL·kg^−1^·min^−1^) completed a graded exercise test (7 × 4-min stages) to establish VO_2_–power output relationships and VO_2_Max. Each participant performed three maximal-effort rowing trials (4MIN, 5MIN10, and 6MIN30) in randomized order over separate days. Trial durations were selected to reflect 1500 m split times based on current 2000 m men’s world record performances across different boat classes. Anaerobic contribution to total energetic metabolism was estimated using the maximal accumulated oxygen deficit (MAOD) method [2]. Maximum power output was assessed via countermovement jump, a 7-stroke rowing test, and a Wingate anaerobic test. Repeated measures ANOVA revealed a significant main effect of trial duration on anaerobic contribution (F(2,16) = 34.1, *p* < 0.001, η^2^ = 0.81) and average power output (F(2,16) = 17.6, *p* < 0.001, η^2^ = 0.69). Anaerobic contribution rose from 17.5 ± 4.0% at 6MIN30 to 27.5 ± 5.2% at 4MIN. Power output increased from 322.3 ± 48.8 W to 360.1 ± 53.4 W. Stroke rate did not differ significantly across trials. Wingate test performance correlated strongly with rowing power output: r = 0.68, r = 0.68, r = 0.79 for 4MIN, 5MIN10 and 6MIN30, respectively (*p* < 0.05) [2]. Average 7-stoke power output was correlated with 6MIN30 only (r = 0.72, *p* < 0.05) Shorter rowing durations substantially increased anaerobic energy reliance and power demands, beyond findings reported in previous literature [3]. These findings suggest targeted training strategies and potential reconsideration of athlete selection criteria for the 1500 m formats at Los Angeles 2028, particularly for the fastest boat classes like the men’s eight, where the anaerobic component is most substantial.

**Keywords:** accumulated oxygen deficit; anaerobic capacity; performance determinants; peak power; time trial

**Funding:** This research received no external funding.

**References**

1. Astridge DJ, Peeling P, Goods PSR, et al. Rowing in Los Angeles: Performance Considerations for the Change to 1500 m at the 2028 Olympic Games. International Journal of Sports Physiology and Performance. 2022;18(1):104–107. doi:10.1123/ijspp.2022-0231.
2. Noordhof DA, de Koning JJ, Foster C. The Maximal Accumulated Oxygen Deficit Method. Sports Med. 2010;40(4):285–302. doi:10.2165/11530390-000000000-00000.
3. Gastin PB. Energy System Interaction and Relative Contribution During Maximal Exercise. Sports Med. 2001;31(10):725–741. doi:10.2165/00007256-200131100-00003.

**Disclaimer/Publisher’s Note:** The statements, opinions and data contained in all publications are solely those of the individual author(s) and contributor(s) and not of MDPI and/or the editor(s). MDPI and/or the editor(s) disclaim responsibility for any injury to people or property resulting from any ideas, methods, instructions or products referred to in the content.

### 2.30. The Effect of Interrupting Sitting with Acute Exercise on Vascular Health in Adults with Long Covid

Nick Hudson ^1,^*, Scott Hannah ^2^, Margaret Husted ^3^, Simon Fryer ^4^, Helen Ryan-Stewart ^5^, Mark Rickenbach ^6,7^, Keeron Stone ^8,9^ and James Faulkner ^1,10^

^1^  School of Sport, Health and Community, University of Winchester, Winchester, United Kingdom n.hudson.17@unimail.winchester.ac.uk
^2^  School of Sport, Health and Community, University of Winchester, Winchester, United Kingdom scott.hannah@winchester.ac.uk
^3^  School of Psychology, University of Winchester, Winchester, United Kingdom margaret.husted@winchester.ac.uk
^4^  School of Education and Science, University of Gloucestershire, Gloucester, United Kingdom sfryer@glos.ac.uk
^5^  Faculty of Education, Humanities and Health Sciences, EIT | Te Pūkenga, Hawke’s Bay, New Zealand. HRyanStewart@eit.ac.nz
^6^  Faculty of Health and Wellbeing, University of Winchester, Winchester, United Kingdom
^7^  Park and St Francis Surgery, Eastleigh, Hampshire, UK. Mark.Rickenbach@nhs.net
^8^  Centre for Cardiovascular Research, Innovation And Development (CURIAD), Cardiff School of Sport and Health Sciences, Cardiff Metropolitan University, Cardiff, Wales, UK kstone@cardiffmet.ac.uk
^9^  National Cardiovascular Research Network, Wales, UK
^10^  Primary Care Research Centre, University of Southampton, Southampton, UK. J.a.faulkner@soton.ac.uk
*  Correspondence: n.hudson.17@unimail.winchester.ac.uk

**Abstract**

Long COVID (LC) is a persistent condition following initial SARS-CoV-2 infection, with approximately 2 million people in the UK currently experiencing self-reported LC (1). People with LC become significantly less active (2), partially explained by fatigue being one of the most common symptoms. Sedentary time is known to have an adverse effect on vascular health and can increase the risk of cardiovascular disease. Sitting periods as short as one hour are known to worsen vascular health, however, interrupting these periods with short bouts of exercise has been shown to mitigate the negative effect on vascular health (3). Arterial stiffness is a measure of vascular health through changes in the structure and function of blood vessels. This study aimed to investigate whether prolonged sitting induces a vascular response in people with LC and if interrupting sitting periods has a beneficial effect. Participants were tasked with sitting still for two hours. Interruptions included sit-to-stands, calf raises, and self-paced walking to mimic activities of daily living. Measures of central and peripheral blood pressure and arterial stiffness (SphygmoCor XCEL) were recorded at baseline and following two hours of interrupted or uninterrupted sitting. Ethical approval was obtained by Health and Care Research Wales (IRAS: 309606 22/SC/0120). Forty-five participants (30 LC, 15 healthy; 52.8 ± 11.7 years, symptoms lasting >12 months) completed both experimental visits. Linear mixed-effects models demonstrated a significant increase in brachial systolic blood pressure (MD = 4.2 mmHg, *p* < 0.001) and mean arterial pressure (MD = 2.9 mmHg, *p* < 0.001) following prolonged sitting regardless of interruptions. There was no significant change in carotid-femoral Pulse Wave Velocity (cfPWV) (*p* = 0.170). This research demonstrates that sitting for 2 h instigates changes in blood pressure, which is thought to be driven by blood pooling in the lower limbs resulting in a decrease in venous return and cardiac output, causing compensations through the sympathetic nervous system including increased peripheral vascular resistance ^4^. Although interrupting sitting did not affect cfPWV, it is unclear whether the intensity of interruption or the duration of sitting time was sufficient to induce changes in arterial stiffness. Additionally, understanding the effect of chronic periods of sedentary behaviour through prolonged sitting on arterial stiffness is important to understand the clinical relevance of increased sedentary behaviour in LC.

**Keywords:** COVID-19 1; Long COVID 2; Arterial stiffness 3; Blood pressure 4

**Funding:** This research was funded by University of Winchester, grant number UoA23_22_Faulk.

**References**

1. Office for National Statistics. Prevalence of ongoing symptoms following coronavirus (COVID-19) infection in the UK. ONS. **2023**.
2. Wright, J.; Astill, S. L.; Sivan, M. The Relationship between Physical Activity and Long COVID: A Cross-Sectional Study. *International Journal of Environmental Research and Public Health* **2022**, *19* (9), 5093.
3. Paterson, C.; Fryer, S.; Zieff, G.; Stone, K.; Credeur, D. P.; Barone Gibbs, B.; Padilla, J.; Parker, J. K.; Stoner, L. The Effects of Acute Exposure to Prolonged Sitting, With and Without Interruption, on Vascular Function Among Adults: A Meta-Analysis. *Sports Med* **2020**, *50* (11), 1929–1942.
4. Adams, N.; Paterson, C.; Poles, J.; Higgins, S.; Stoner, L. The Effect of Sitting Duration on Peripheral Blood Pressure Responses to Prolonged Sitting, With and Without Interruption: A Systematic Review and Meta-Analysis. Sports Med **2023**, 54, 169–183.

**Disclaimer/Publisher’s Note:** The statements, opinions and data contained in all publications are solely those of the individual author(s) and contributor(s) and not of MDPI and/or the editor(s). MDPI and/or the editor(s) disclaim responsibility for any injury to people or property resulting from any ideas, methods, instructions or products referred to in the content.

### 2.31. Adaptations in Golf Swing Performance Following 16-Week Strength Training Intervention in Elite Golfers

Martin Juel Johansen ^1,^*, Per Aagaard ^1^, Kasper Degn Gejl ^1^ and Thue Kvorning ^1^ y Jens Bojsen-Møller ^1^

^1^  Department of Sports Science and Clinical Biomechanics, University of Southern Denmark, Odense, Denmark
*  Correspondence: Mjohansen@health.sdu.dk

**Abstract**

Driving distance and club head speed (CHS) are key determinants of elite golf performance and show positive associations with competitive success. Although previous studies suggest that strength training can improve CHS and overall golf performance, evidence among elite populations—particularly elite female golfers—remains limited.

The present study investigated the effect of a 16-week periodized heavy-resistance strength training program on mechanical muscle function and golf swing performance in elite golfers, with a particular focus on changes in maximal CHS, ball speed, and driving distance.

Forty elite Danish National Team golfers participated, of whom 30 (16 women, 14 men) completed the training intervention, while 10 served as controls and continued their habitual training. The intervention consisted of a full-body resistance training program (12 RM to 5 RM loading) performed four times weekly over 16 weeks.

Participants completed pre- and post-intervention assessments of physical performance (muscle strength, rate of force development, power) via isometric mid-thigh pull, isometric bench press, countermovement jump, and standing cable trunk rotation tests. Golf swing performance was evaluated using Trackman technology and force plates.

Preliminary findings indicate substantial improvements in muscle function and golf swing metrics following the intervention. Absolute driver CHS increased by 7.3 km/h in men and 6 km/h in women (~4% improvement). Maximal isometric mid-thigh pull peak force increased by 11% across sexes. Women improved maximal isometric bench press strength by 7%, whereas men improved by 5%. Countermovement jump peak power increased by ~6% in both groups, while standing cable trunk rotation peak power improved by ~15%.

This study addresses key gaps regarding physiological and performance adaptations to strength training in elite golfers, with particular emphasis on sex-specific responses. Findings support the development of individualized, evidence-based training strategies for elite golfers.

**Keywords:** Golf; biomechanics; muscle strength; muscle power; strength and conditioning

**Funding:** This project was supported by the Team Danmark Elite Sports Association through a grant from the Novo Nordisk Foundation (NNF22SA0078293) and by the Danish Ministry of Culture.

**References**

1. Hellström J., Nilsson J., Isberg L. Drive for dough. PGA Tour golfers’ tee shot functional accuracy, distance and hole score. Journal of Sports Sciences, 2014; 32(5): 462–469.
2. Robinson L., Murray A., Ehlert A., Wells J., Jarvis P., Turner A., Glover D., Coughlan D., Hembrough R., Bishop C. Effects of Physical Training and Associations Between Physical Performance Characteristics and Golf Performance in Female Players: A Systematic Review With Meta-Analysis. Journal of Strength and Conditioning Research, 2024; 38(2): 374–383.
3. Uthoff A., Sommerfield L. M., Pichardo A. W. Effects of Resistance Training Methods on Golf Clubhead Speed and Hitting Distance: A Systematic Review. Journal of Strength and Conditioning Research, 2021; 35(9): 2651–2660.

### 2.32. Changes in Muscular Strength, Exercise Adherence, and Self-Determinant Motivation over 24 Weeks of Resistance Exercise: The BoneWheel Study

Benjamin T Karlsen ^1,^*, Linn C Risvang ^1^, Vegard Strøm ^1,2^, Truls Raastad ^1^ and Kristin L Jonvik ^1^

^1^  Norwegian School of Sport Sciences, Norway
^2^  Sunnaas Rehabilitation Hospital, Norway
*  Correspondence: benjamintk@student.nih.no

**Abstract**

Resistance exercise is a reliable and effective type of exercise for improving physical function, reducing shoulder pain, and reducing negative health outcomes in manual wheelchair users. Methodological limitations restrict the current evidence of the effectiveness of resistance exercise and manual wheelchair users. The primary aim of this study was to investigate the effects of a 24-week bone-specific resistance exercise program on isometric and dynamic muscle strength in manual wheelchair users. The secondary aim was to investigate the association between adherence to the exercise program and change in muscular strength. And thirdly, to evaluate whether self-determination could be a cofounding factor for adherence to the exercise program. In this multi-centre randomized controlled trial, wheelchair users were randomly allocated to either upper body resistance exercise (3 sessions per week over 24 weeks) combined with nutrition optimization (EX, n = 14), or nutrition optimization only (CON, n = 19). At pre-, mid-, and post-intervention, we measured maximal strength of isometric overhead and bench press, prone row and supine pull, dynamic overhead and incline bench press. The participants filled in the behavioral regulation to exercise questionnaire (BREQ-2) to measure motivation. The resistance training program consisted of six upper-body exercises aiming to load the lumbar spine and hip. Adherence was evaluated based on the training logs filled in by the participants throughout the intervention. Forty-five WCU (SCI: 49%, cerebral palsy: 27%, other: 24%; 40% female; age: 37 ± 9 y; mass: 67 ± 19 kg) participated, of which 33 completed the study. EX improved more than CON in 5 out of 6 exercises, where ISO-PR (13.0% (8.9) vs. 4.5% (8.8), *p* = 0.023), DYN-OH (21.8% (15.0) vs. 1.7% (11.7), *p* = 0.001) and DYN-IBP (16.8% (11.8) vs. −1.5% (13.5), *p* = 0.001) were significantly greater than CON. The participants of the EX group performed 81.9% of the scheduled exercise sessions and showed high levels of autonomous motivation. Associations between adherence and improvements in strength were found for both the 1 RM test and isometric supine pull (r ≤ 0.653, *p* ≤ 0.05). No associations were found between self-determined motivation and adherence. In conclusion, 24 weeks of resistance training increased maximal upper body strength by 3.5–21.8%. The selection was scarce and had a high level of heterogeneity, leaving some of the findings inconclusive. It can be concluded that this protocol proved to be effective for increasing muscle strength, but inconclusive for maintaining exercise adherence. Furthermore, motivation was not seen as a contributing factor for exercise adherence. 

**Keywords:** strength training; spinal cord injury; paraplegia; adherence; self-determination

**Funding:** This research was funded by Foundation Dam, grant number 2022/FO387192.

**Disclaimer/Publisher’s Note:** The statements, opinions and data contained in all publications are solely those of the individual author(s) and contributor(s) and not of MDPI and/or the editor(s). MDPI and/or the editor(s) disclaim responsibility for any injury to people or property resulting from any ideas, methods, instructions or products referred to in the content.

### 2.33. The Effect of Bicarbonate on Sprint Performance in Ski Ergometer; A Comparison Between Oral Supplementations—Preliminary Results

Elise Lander ^1^, Andreas V. Kleive ^1^, Kristian Solem ^1^, Olav Vikmoen ^1^, Øyvind Skattebo ^1^, Thomas Johansen Losnegard ^1^, Ina Garthe ^2^ and Truls Raastad ^1,^*

^1^  Department of Physical Performance, Norwegian School of Sport Sciences; elisej@nih.no
^2^  Department of sports nutrition, Olympiatoppen; ina.garthe@olympiatoppen.no
*  Correspondence: trulsr@nih.no

**Abstract**

Ingesting 0.3 g bicarbonate per kg body mass prior to sport competition may improve performance, especially in competitions with high intensity and a duration of 1–12 min (Grgic et al., 2021). The effect of bicarbonate seems to delay fatigue, giving the possibility to maintain the high intensity for a longer period or with reduced recovery time. Bicarbonate supplementation in a cross-country ski-sprint competition, with 30 min separating 2–4 min of maximal performance between finals, could therefore potentially improve performance, but has a risk of gastro-intestinal (GI) problems. Currently, two main strategies are used to alleviate GI problems—dividing into 4–6 smaller doses or dissolving bicarbonate tablets into a hydrogel formula (Maurten™). The purpose was therefore to investigate any differences in the delivery effectiveness and GI-problems between the two different bicarbonate supplements. Ten elite cross-country athletes will be recruited and here we report on the first four (F = 3, M = 1, 20–22 y, double poling VO2max: 55.6 ± 5.6 mL⋅kg^−1^⋅min^−1^). Participants met in the lab 1 h post breakfast for three different test days and performed 4 maximal sprints of 2.5 min on a ThoraxTrainer ski ergometer. Sprint 2–4 was performed 1.5 h after sprint 1, with 30 min rest between sprints. Prior to sprint 1, it was served a sports drink with (BiF) or without (PLA) 0.3 g⋅kg^−1^ bicarbonate (six doses every 30 min), which was blinded, or a hydrogel with 0.3 g⋅kg^−1^ bicarbonate 1.75 h before sprint 1 (BiM). Carbohydrate intake during the day was standardized for each participant and matched for all test-days. Blood samples were collected during the test day and after each sprint. GI problems were assessed with questionnaires at the end of the day. A two-way ANOVA, with factors time and supplement, was used to assess the changes. Bicarbonate, pH and buffer capacity (cBase) increased before sprint 1 with BiF and BiM (*p* < 0.0001), and not with PLA, and decreased after sprint 1–4 with all supplements (*p* < 0.05). BiF resulted in higher bicarbonate levels (sprint 1–3, *p* < 0.01, sprint 4, *p* = 0.07), cBase (sprint 1–4, *p* < 0.05) and pH (sprint 1, *p* < 0.01) following the sprints compared to PLA, and was not different from BiM. Sprint performance, GI problems or changes in lactate and glucose levels were not different between supplements. Both bicarbonate supplementation strategies were efficient to increase bicarbonate levels and buffer capacity and reported low GI problems. The higher bicarbonate levels and buffer capacity may lead to improved performance, which remains to be elucidated with more participants included.

**Keywords:** bicarbonate; cross-country; supplement strategy; endurance performance

**Funding:** This research was funded by Fuel of Norway.

**Reference**

1. Grgic, J., Pedisic, Z., Saunders, B., Artioli, G. G., Schoenfeld, B. J., McKenna, M. J., Bishop, D. J., Kreider, R. B., Stout, J. R., Kalman, D. S., Arent, S. M., VanDusseldorp, T. A., Lopez, H. L., Ziegenfuss, T. N., Burke, L. M., Antonio, J., & Campbell, B. I. International Society of Sports Nutrition position stand: sodium bicarbonate and exercise performance. *JISSN* **2021***, 18(1)*.

**Disclaimer/Publisher’s Note:** The statements, opinions and data contained in all publications are solely those of the individual author(s) and contributor(s) and not of MDPI and/or the editor(s). MDPI and/or the editor(s) disclaim responsibility for any injury to people or property resulting from any ideas, methods, instructions or products referred to in the content.

### 2.34. Temporal Dynamics of Motor Neuron Excitability During Prolonged Static Stretching

Denis Vieira ^1,2,^*, Martim Bottaro ^3^, Marion Hitier ^2^, João Durigan ^3^ and Nicolas Babault ^2^

^1^  Programa de Pós-Graduação em Educação Física—Universidade Católica de Brasília
^2^  INSERM CAPS U1093 & Centre d’Expertise de la Performance—Université Bourgogne Europe
^3^  Faculdade de Educação Física—Universidade de Brasília
*  Correspondence: denis.vieira@p.ucb.br

**Abstract**

Static stretching is commonly included in training routines; however, the neuromuscular behavior during prolonged static stretching, particularly with different stretch amplitudes, remains poorly understood [1,2]. This study aimed to investigate the neuromuscular and muscle-tendon complex responses during prolonged static stretching performed with supramaximal and submaximal amplitudes, as well as their immediate acute effects. Thirteen young participants completed three randomized experimental sessions: (1) static stretching of the plantar flexors at a supramaximal amplitude (dorsiflexion 5° above maximal range of motion), (2) static stretching at a submaximal amplitude (dorsiflexion 5° below maximal range of motion), and (3) a control session with the ankle in a neutral position. Each session lasted 15 min. Motor neuronal excitability of the soleus muscle (H_max_/M_max_ ratio) was assessed before (PRE), every five minutes during (0, 5, 10, and 15 min), and immediately after (POST) each session. PRE and POST measures were performed with the ankle in a neutral position, whereas the assessment at every five minutes during each experimental condition were conducted with the ankle positioned according to each respective condition. A Generalized Mixed Model was applied, with participants as a random effect, time and condition as fixed effects, and the 0-min value as a covariate to control for joint position effects. Fisher’s LSD post hoc test was used when significant main effects or interactions were found. A main effect of time and a time × condition interaction was found for the H_max_/M_max_ ratio (*p* < 0.05). Motor neuron excitability was reduced at the beginning of both stretching conditions compared to PRE (*p* < 0.05). Additionally, excitability was lower at 15 min compared to the beginning of the stretch (i.e., 0 min), regardless of stretch amplitude (*p* < 0.05). However, no significant differences were found across time points in the control session (*p* > 0.05), nor between PRE and POST in any condition (*p* > 0.05). In conclusion, prolonged static stretching does not cause lasting changes in motor neuron excitability, regardless of stretch amplitude. The observed reductions during stretching appear to be transient, with excitability returning quickly to baseline once the stretch is released.

**Keywords:** Hoffman reflex; H_max_/M_max_ ratio; Neuromuscular performance; Afferent Ia; Spinal excitability

**Funding:** This research was funded by Fundação de Apoio a Pesquisa do Distrito Federal FAP/DF (grant numbers 00193-00001372/2024-82 and 00193-00001661/2024-81), and Conselho Nacional de Desenvolvimento Científico e Tecnológico—CNPq (grant number 446576/2024-7).

**References**

1. Behm, D.G.; Chaouachi, A. A Review of the Acute Effects of Static and Dynamic Stretching on Performance. *Eur J Appl Physiol* **2011**, *111*, 2633–2651.
2. Behm, D.G.; Button, D.C.; Butt, J.C. Factors Affecting Force Loss with Prolonged Stretching. *Canadian Journal of Applied Physiology* **2001**, *26*, 261–272.

**Disclaimer/Publisher’s Note:** The statements, opinions and data contained in all publications are solely those of the individual author(s) and contributor(s) and not of MDPI and/or the editor(s). MDPI and/or the editor(s) disclaim responsibility for any injury to people or property resulting from any ideas, methods, instructions or products referred to in the content.

### 2.35. A Glucosinolate-Rich Beverage Lowers Blood Lactate Concentrations During Submaximal Exercise

**Fredrik Vinge, Emma Tillqvist, Oscar Horwath, William Apró, Filip J Larsen and Michaela L Sundqvist**

**Abstract**

Glucosinolate-rich broccoli sprouts combined with intense exercise training for 7 days have been shown to reduce blood lactate concentrations during exercise, attenuate hypoglycemic events, improve physical performance, and reduce markers of oxidative stress. This study aimed to investigate the acute, dose-dependent effects of glucosinolate-rich red kale sprouts (GRS) on blood lactate and blood glucose following the ingestion of three different doses.

Fifteen healthy participants consumed 37.5 g or 75 g of GRS or an isocaloric placebo blended into a beverage on three separate occasions. The participants cycled on an ergometer at three submaximal work rates before and three hours after ingestion. Measurements of oxygen uptake, substrate-level oxidation, blood lactate, blood glucose, and ratings of perceived exertion were taken before and after each cycling interval.

The intake of glucosinolate-rich sprouts acutely decreased blood lactate levels during submaximal cycling and increased blood glucose levels at rest. The largest reduction in blood lactate was observed at the 37.5 g dose compared to placebo, where the concentration was 0.4 ± 0.2 mM lower at work rate close to threshold (*p* = 0.003). For the 75 g dose, the reduction in blood lactate was 0.25 ± 0.1 mM (*p* = 0.02). No significant effects were seen in the lowest work rate. The mean resting glucose level was 3.9 ± 0.1 mM following placebo, compared to 4.3 ± 0.1 mM after intake of either 37.5 g or 75 g dose (*p* < 0.01, respectively).

These findings suggest that glucosinolate-rich red kale sprouts have a lactate-lowering effect during submaximal efforts, which may have important implications for supplementation for improving endurance performance.

**Competing Interest Statement:** Authors MLS and FJL hold a patent related to the use of isothiocyanates for enhancing adaptations to exercise training. In addition, they are co-founders and shareholders in a commercial operational entity, which aims to market and sell products designed to augment athletic performance. No other authors have any conflicts of interest to declare.

This article has been published as a preprint:

https://www.biorxiv.org/content/10.1101/2025.04.15.648889v1.

### 2.36. Short-Term Exercise Effects on Cardiovascular Health and Muscle Function in Patients with Severe Mental Illness: A Randomized Controlled Trial

Mikel L. Sáez de Asteasu ^1,2,^*, Arantxa Ancín-Osés ^1^, Rubén Auré-Sánchez ^1^, Paula Etayo-Urtasun ^1^, Imanol Reparaz-Escudero ^1^, Ana Urteaga Villanueva ^3^, Manuel J. Cuesta ^3^, Juan I. Arrarás ^3^, Amalia Zarzuela ^3^ and Mikel Izquierdo ^1,2^

^1^  Navarrabiomed, Hospital Universitario de Navarra (HUN)-Universidad Pública de Navarra (UPNA), IdiSNA, Pamplona (Spain). mikel.lopez@unavarra.es; arantxa.ancin@opendeusto.es; rauresan@gmail.com; paulaetayo12@gmail.com; ireparaz@cita-alzheimer.org; mikel.izquierdo@gmail.com
^2^  CIBER of Frailty and Healthy Aging (CIBERFES), Instituto de Salud Carlos III, Madrid (Spain)
^3^  Mental Health Rehabilitation Unit, Navarre Health Service, Pamplona (Spain), aurtevillanueva@gmail.com; mj.cuesta.zorita@navarra.es; raras.urdaniz@navarra.es; lia.zarzuela.ituarte@navarra.es
*  Correspondence: mikel.lopez@unavarra.es; Tel.: (+34) 848423199

**Abstract**

People with mental illness have 10–20 years reduced life expectancy. The leading causes of premature death are lifestyle-related illnesses, in particular cardiovascular disease (CVD). Physical inactivity seems to be one of the main factors in CVD development. Objective: To investigate the effects of a physical exercise intervention on cardiovascular health and muscle function in adults with severe mental illness. Methods: Patients admitted to the Mental Health Rehabilitation Center (Navarre) were randomly divided into the intervention group (IG) or the control group (CG). The CG received standard medical care, with cognitive-behavioral therapy. The IG participated in a 6-week, 2 sessions/week multicomponent physical exercise program. The percentage of body fat and body mass index (BMI), muscle function with maximal strength (1 RM), and power for leg-press and knee extension exercises were evaluated. Results: 41 patients were included in the study, 21 in IG and 20 in CG. Participants were predominantly male (63.41%) with a mean age (SD) of 42.5 (11) years. The diagnosis of the study population were psychotic disorders (76.1%), major depressive and bipolar disorders. Significant improvements were observed in muscle power output during leg press exercises (∆ 101.3 W vs. 0.90 W; *p* = 0.004) and knee extension exercises (∆ 106.41 W vs. −6.47 W; *p* < 0.001) after the intervention in the IG. Additionally, IG demonstrated greater improvements in muscle strength, increasing weight lifted during the 1 RM leg press (∆ 47.78 kg vs. 1.29 kg; *p* < 0.002) and knee extension exercises (∆ 16.17 kg vs. −1.88 kg; *p* < 0.001). These improvements were not observed in CG. Furthermore, the IG experienced a significant reduction in body fat compared to the CG (∆ −0.66% vs. 0.78%; *p* < 0.004). No significant BMI differences were found between the groups.

Conclusion: Short-term multicomponent exercise program (6 weeks, 2 sessions/week) appears to be effective in improving muscle function and body composition in adults with severe mental illness.

**Keywords**: severe mental illness, cardiovascular health, muscle function, physical exercise

**Funding**: This research was funded by GOBIERNO DE NAVARRA.

**References**

1. Brobakken, M. F.; Nygård, M.; Wang, E. Physical Health Impairment and Exercise as Medicine in Severe Mental Disorders: A Narrative Review. Sports Med.—Open 2022, 8 (1), 115.
2. Vancampfort, D.; Firth, J.; Schuch, F. B.; Rosenbaum, S.; Mugisha, J.; Hallgren, M.; Probst, M.; Ward, P. B.; Gaughran, F.; De Hert, M.; Carvalho, A. F.; Stubbs, B. Sedentary Behavior and Physical Activity Levels in People with Schizophrenia, Bipolar Disorder and Major Depressive Disorder: A Global Systematic Review and Meta-Analysis. World Psychiatry 2017, 16 (3), 308–315.
3. Stubbs, B.; Vancampfort, D.; Hallgren, M.; Firth, J.; Veronese, N.; Solmi, M.; Brand, S.; Cordes, J.; Malchow, B.; Gerber, M.; Schmitt, A.; Correll, C. U.; De Hert, M.; Gaughran, F.; Schneider, F.; Kinnafick, F.; Falkai, P.; Möller, H.-J.; Kahl, K. G. EPA Guidance on Physical Activity as a Treatment for Severe Mental Illness: A Meta-Review of the Evidence and Position Statement from the European Psychiatric Association (EPA), Supported by the International Organization of Physical Therapists in Mental Health (IOPTMH). Eur. Psychiatry 2018, 54, 124–144.
4. Vancampfort, D.; Firth, J.; Stubbs, B.; Schuch, F.; Rosenbaum, S.; Hallgren, M.; Deenik, J.; Ward, P. B.; Mugisha, J.; Van Damme, T.; Werneck, A. O. The Efficacy, Mechanisms and Implementation of Physical Activity as an Adjunctive Treatment in Mental Disorders: A Meta-Review of Outcomes, Neurobiology and Key Determinants. World Psychiatry 2025, 24 (2), 227–239.

**Disclaimer/Publisher’s Note:** The statements, opinions and data contained in all publications are solely those of the individual author(s) and contributor(s) and not of MDPI and/or the editor(s). MDPI and/or the editor(s) disclaim responsibility for any injury to people or property resulting from any ideas, methods, instructions or products referred to in the content.

### 2.37. Self-Perceived Physical Fitness in Spanish Children and Adolescents: The PASOS Study

Elena Marín-Cascales ^1,2,3,^*, Tomás T. Freitas ^1,2,3,4^, Konstantinos Spyrou ^1,2,3^, Santiago F. Gómez ^5,6,7,8^, Helmut Schröder ^6,7^, Pedro E. Alcaraz ^1,2,3^ and Consorcio PASOS

^1^  UCAM Research Center for High Performance Sport, UCAM Universidad Católica de Murcia, 30107 Murcia, Spain; emarin@ucam.edu; tfreitas@ucam.edu; kspyrou@ucam.edu; palcaraz@ucam.edu
^2^  Facultad de Deporte, UCAM Universidad Católica de Murcia, 30107 Murcia, Spain
^3^  Strength & Conditioning Society, 30008 Murcia, Spain
^4^  NAR-Nucleus of High Performance in Sport, São Paulo 04753-060, Brazil
^5^  Gasol Foundation Europe, 08830 Sant Boi de Llobregat, Barcelona, Spain
^6^  CIBER de Epidemiología y Salud Pública (CIBERESP), Instituto de Salud Carlos III, 28049 Madrid, Spain
^7^  Cardiovascular Risk and Nutrition Research Group (CARIN), Hospital del Mar Research Institute, Barcelona, Spain
^8^  Nursing and Physiotherapy Department, University of Lleida, 25008 Lleida, Spain
*  Correspondence: emarin@ucam.edu

**Abstract**

Physical fitness (PF) is a crucial measure of the functional status of several systems of the human body (e.g., musculoskeletal, cardiorespiratory, endocrine–metabolic, etc.) that is directly related to the capacity to perform different types of physical activity (PA) without excessive fatigue (1). Hence, PF can be considered an important health marker, particularly in childhood and adolescence, life stages in which a multitude of physiological and psychological changes occur and that greatly influences health status, and behaviors and lifestyle during adulthood (1, 2). This study aimed to analyze the perception of Spanish children and adolescents of their PF (i.e., general PF, cardiovascular fitness, strength, speed and flexibility), and to examine the relationship between PA levels and self-perceived PF in this population. A nationwide representative sample of 6804 participants aged 8–16 years (children: n = 3063; adolescents: n = 3741) included in the 2019 and 2022 editions of the PASOS (Physical Activity, Sedentarism, and Obesity in Spanish Youth) study was recruited. Self-reported PA levels and self-perceived PF were assessed through validated questionnaires (the PAU-7S and the International Fitness Scale, respectively). A frequency multinomial analysis was performed to identify differences between children and adolescents on the percentage of the responses regarding all perceived PF components. Moreover, a multinomial logistic regression was conducted to determine the relationship between the perception of general PF and the minutes of PA per week. The results indicated that a “very good” perception in all PF components was noted for children when compared to adolescents (p ranging from < 0.001 to 0.03). The amount of weekly PA was associated with reduced odds of having a “very bad”, “bad”, “acceptable” or “good” perceived general PF with respect to a “very good” perception, regardless of the developmental stage. Finally, adolescents were more likely to perceive their PF as “very bad”, “bad”, “acceptable” or “good” compared to “very good”, relative to children, when controlling for the amount of PA per week. In summary, Spanish children perceive their PF as superior across all its components. Furthermore, PA levels influence the perception of PF in Spanish youth, irrespective of the developmental stage.

**Keywords:** health, childhood; exercise; self-perception; school; physical education

**Funding:** The PASOS study was funded by Fundación PROBITAS (2019) and the Gasol Foundation (2019–2020). Additional funds were received from the Barça Foundation (2019–2020), Banco Santander (2019), IFA (2019–2020), Vienna (2019), and the Fundación Deporte Joven (2019) (no references are applicable). J.A.T., M.G.-G. and C.B. are funding by Instituto de Salud Carlos III through the CIBEROBN CB12/03/30038, which are co-funded by the European Regional Development Fund.

**References**

1. Ortega, F.B.; Ruiz, J.R.; Castillo, M.J.; Sjöström, M. Physical fitness in childhood and adolescence: A powerful marker of health. *Int. J. Obes*. **2008**, 32, 1–11.
2. Menotti, A.; Puddu, P.E.; Geleijnse, J.M.; Kafatos, A.; Tolonen, H.J. Physical activity and physical fitness in prediction of all-cause mortality and age at death in European extinct cohorts of middle-aged men followed for 60 years. *Eur. J. Prev. Cardiol.* **2024**, *31*, 1441–1448.

**Disclaimer/Publisher’s Note:** The statements, opinions and data contained in all publications are solely those of the individual author(s) and contributor(s) and not of MDPI and/or the editor(s). MDPI and/or the editor(s) disclaim responsibility for any injury to people or property resulting from any ideas, methods, instructions or products referred to in the content.

### 2.38. Longitudinal Development of Anthropometrics and Physical Performance in Adolescent Football Players

Lars M. Tingelstad ^1,^*, Truls Raastad ^1^, Grethe Myklebust, Thor Einar Gjerstad Andersen, Bård Erlend Solstad, Jesper Barth Bugten and Live S. Luteberget

^1^  Department of physical performance, Norwegian School of Sport Science, Oslo, Norway
^2^  Oslo Sport Trauma Research Center, Department of sports medicine, Norwegian School of Sport Science, Oslo, Norway
^3^  Department of Sport Science and Physical Education, University of Agder, Kristiansand, Norway
*  Correspondence: larsmt@nih.no

**Abstract**

Adolescence is a critical period for athletic development, marked by rapid physiological changes, increases in height and body mass, and improved physical performance, alongside growing divergence between boys and girls (1). However, longitudinal studies including both sexes remain scarce. This study aimed to examine age- and sex-specific differences in physical performance development in adolescent team sport athletes and explore their relationship with maturation. Using a repeated measures design, 248 football and handball players (U14: 71 boys, 77 girls; U16: 52 boys, 48 girls) were tested across three timepoints over two years for height and weight, and a physical test battery including test for sprint (10 m and 30 m) Change of Direction (CoD), and strength (Hamstring, hip abduction, hip adduction and leg press). Years from Peak Height Velocity (YPHV) was calculated using the Mirwald equation. Height and body mass increased for all groups, with greater gains in boys and in the U14 age group for both sexes. Both boys and girls significantly improved physical performance, but boys demonstrated significantly greater gains than girls, particularly among the U14 age group. U14 boys improved significantly more than U16 boys in sprint (10 m: −0.14 s vs. −0.07 s; 30 m: −0.41 s vs. 0.21 s), CoD (−0.83 s vs. −0.49 s), and hamstring (96 N vs. 60 N) and hip adduction strength (48 N vs. 27 N). U14 girls improved significantly more in sprint compared to U16 girls (T1–2; 10 m: −0.08 s vs. −0.04 s, 30 m: −0.17 s vs. −0.06 s, T1–3: 30 m sprint −0.21 s vs. −0.03 s). Apart from U14 boys, no group significantly improved relative force, hamstring, or hip adduction strength. Baseline YPHV, growth, and sex was significantly associated with performance development, though no sex × YPHV interaction was found. Considerable individual variation was observed across all groups. Age, sex, and maturation significantly influenced physical development, with greater improvements in boys, younger athletes, and those with lower baseline YPHV. While trends were consistent, the magnitude of development varied by sex and performance measure. The considerable individual variation underscores the need for tailored, age- and sex-specific training strategies aligned with each athlete’s developmental trajectory. Given the high incidence of hamstring and groin injuries in football (2–3), the lack of significant improvements in relative strength suggests that current training protocols should be refined to better address athlete specific needs and injury prevention.

**Keywords**: Growth & maturation, physical performance, adolescent athletes, sex differences, longitudinal development

**Funding**: This research received no external funding.

**References**

1. Malina, R. M., Bouchard, C., & Bar-Or, O. (2004). Growth, Maturation, and Physical activity. Human Kinetics.
2. Light, N., Thorborg, K., Krommes, K., Nielsen, M. F., Thornton, K. B., Hölmich, P., Penalver, J. J. J., & Ishoi, L. (2022). Rapid spike in hip adduction strength in early adolescent footballers: A study of 125 elite male players from youth to senior. International Journal of Sports Physiology and Performance, 17(9), 1407–1414. https://doi.org/10.1123/ijspp.2022-0025.
3. Sancese, A., Taylor, L., Walsh, G., Byrd, E., & Delextrat, A. (2023). Effects of sprint versus strength training on risk factors for hamstring injury in football players. Journal of Sports Medicine and Physical Fitness, 63(4), 580–587. https://doi.org/10.23736/S0022-4707.22.14529-9.

**Disclaimer/Publisher’s Note:** The statements, opinions and data contained in all publications are solely those of the individual author(s) and contributor(s) and not of MDPI and/or the editor(s). MDPI and/or the editor(s) disclaim responsibility for any injury to people or property resulting from any ideas, methods, instructions or products referred to in the content.

### 2.39. Effects of Weight Cutting on Physical and Cognitive Function in Natural Bodybuilders

Kenneth H. Mertz ^1,^*, Bjørk W. Helge ^1^, Jakob Agergaard ^1^, Sara D. Pedersen ^1^ and Jesper L. Andersen ^1^

Institute of Sports Medicine Copenhagen, Bispebjerg and Frederiksberg Hospital, Denmark

*  Correspondence: Kenneth.hudlebusch.mertz@regionh.dk

**Abstract**

Manipulation of body weight and composition is common practice within many sporting disciplines in an effort to improve performance in weight dependent events^1^. However, few studies have investigated the effects of weight cutting (CUT) on physical and cognitive function in well-trained individuals. The aim of the present study was to investigate the effects of prolonged CUT on muscle strength and power, as well as cognitive function in natural bodybuilders. 23 natural bodybuilders (13 females/10 males, mean age 29.8 ± 8.8 years) competing in the 2024 season, were included in the study. Participants were investigated before initiating CUT (Baseline), after 1 month CUT (1 mDiet), 1–2 weeks prior to competition (COMP), and 1 (1 mPost) and 6 months (6 mPost) into the off-season. At all timepoints we performed assessments of body composition using DXA, isokinetic muscle strength of the knee extensors, counter movement jumping (CMJ) height and power using dual force plates, and reaction time using CANTAB digital assessments. Athletes decreased body weight throughout the CUT period compared to baseline (1 mDiet: −2.0 ± 0.4 kg, *p* = 0.0003; COMP: −9.5 ± 0.8 kg, *p* < 0.0001). After the competition, rapid regain of weight was observed at 1 mPost compared to COMP (+6.5 ± 1.3 kg, *p* = 0.002), and body weight was normalized compared to baseline at 6 mPost (+1.6 ± 1.0 kg, *p* = 0.49). During CUT, marked decreases in body fat percentage was observed at 1 mDiet (−2.3 ± 0.2%, *p* < 0.00001), and COMP (−9.1 ± 0.8%, *p* < 0.00001), and returned to baseline levels at 6 mPost (+1.9 ± 1.0%, *p* = 0.37). Lean body mass decreased at COMP (−1.6 ± 0.5 kg, *p* = 0.05), but did not differ from baseline at other time points. Decreases in absolute knee extensor strength was observed at COMP (−17.5 ± 4.3 Nm, *p* = 0.004) and 1 mPost (−18.7 ± 3.8 Nm, *p* = 0.001). CMJ height did not change significantly compared to baseline at any time point. CMJ peak power decreased at COMP (−623 ± 77 W, *p* < 0.0001) compared to baseline, and was normalized at 6 mPost (−78 ± 58 W, *p* = 0.68). Reaction time increased from at COMP (+10.9 ± 3.6 ms, *p* = 0.02), and normalized at 6 mPost (−6.0 ± 4.1 ms, *p* = 0.33). These data show that prolonged weight cutting is associated with marked impairments in both physical and cognitive function, which are normalized when baseline body composition is restored. 

**Keywords:** Bodybuilding, RED-S, Low energy availability, muscle strength, cognition

**Funding:** This research was funded by The Danish Ministry of Culture, grant number SUAKPKfor2W.2024-020”.

**Reference**

1. Melin, A.K.; Areta, J.L; Heikura, I. A;, Stellingwerff, T; Torstveit, M. K; Hackney, A. C. Direct and indirect impact of low energy availability on sports performance. *Scand J Med Sci Sports* **2024**, *34*.

**Disclaimer/Publisher’s Note:** The statements, opinions and data contained in all publications are solely those of the individual author(s) and contributor(s) and not of MDPI and/or the editor(s). MDPI and/or the editor(s) disclaim responsibility for any injury to people or property resulting from any ideas, methods, instructions or products referred to in the content.

### 2.40. Nutrition for Food, Sports and Training in Long-Distance Road Cycling, Including Competing and Recovery

Álvaro Miguel-Ortega ^1,2^, María Azucena Rodríguez-Rodrigo ^2^ and Julio Calleja-González ^3,4,^*

^1^  Faculty of Education, Alfonso X “El Sabio” University (UAX), 28691 Madrid, Spain; amiguort@uax.es
^2^  Regional Ministry of Castilla y León Board of Education, HS Conde Diego Porcelos, 09006 Burgos, Spain; mrodriguezrrod@educa.jcyl.es
^3^  Physical Education and Sports Department, Faculty of Education and Sport, University of the Basque Country (UPV/EHU), 01007 Vitoria, Spain; julio.calleja.gonzalez@gmail.com
^4^  Faculty of Kinesiology, University of Zagreb, 10110 Zagreb, Croatia

**Abstract**

A comprehensive search of academic databases (PubMed, Scopus and Web of Science) was conducted up to May 2025. Studies addressing aspects related to nutrition, supplementation and recovery in long-distance cyclists, including observational studies, clinical trials and reviews, were included. The methodological quality of the studies was assessed using standardised tools. Studies show that consuming an adequate amount of carbohydrates before and during exercise improves performance and maintains muscle glycogen levels [1]. Electrolyte supplements help to prevent imbalances and cramps. Consuming protein after running promotes muscle repair and reduces recovery time [2]. Additionally, strategies such as optimised hydration, increased antioxidant consumption and personalised nutritional planning have been shown to significantly enhance overall recovery [3]. Specific nutritional practices before, during and after long-distance cycling are essential for optimising performance and speeding up recovery. It is recommended that planning is individualised based on the cyclist’s energy needs, the environmental conditions, and the event’s duration. However, further research is needed to establish standardised protocols for optimising these factors in different contexts.

**Keywords:** road cycling; nutrition; recovery; injuries; ergogenic aids

**References**

1. Jeukendrup, A. A Step towards Personalized Sports Nutrition: Carbohydrate Intake during Exercise. Sport. Med. 2014, 44 (SUPPL.1), 25. https://doi.org/10.1007/s40279-014-0148-z.
2. D’Lugos, A. C.; Luden, N. D.; Faller, J. M.; Akers, J. D.; McKenzie, A. I.; Saunders, M. J. Supplemental Protein during Heavy Cycling Training and Recovery Impacts Skeletal Muscle and Heart Rate Responses but Not Performance. Nutrients 2016, 8 (9), 550. https://doi.org/10.3390/nu8090550.
3. Judge, L. W.; Bellar, D. M.; Popp, J. K.; Craig, B. W.; Schoeff, M. A.; Hoover, D. L.; Fox, B.; Kistler, B. M.; Al-Nawaiseh, A. M. Hydration to Maximize Performance and Recovery: Knowledge, Attitudes, and Behaviors among Collegiate Track and Field Throwers. J. Hum. Kinet. 2021, 79 (1), 111–122. https://doi.org/10.2478/hukin-2021-0065.

### 2.41. Biomechanical Analysis of the Power Clean and Its Impact on Shoulder Injuries

Álvaro Miguel-Ortega ^1,2^, María Azucena Rodríguez-Rodrigo ^2^ and Julio Calleja-González ^3,4,^*

^1^  Faculty of Education, Alfonso X “El Sabio” University (UAX), 28691 Madrid, Spain; amiguort@uax.es
^2^  Regional Ministry of Castilla y León Board of Education, HS Conde Diego Porcelos, 09006 Burgos, Spain; mrodriguezrrod@educa.jcyl.es
^3^  Physical Education and Sports Department, Faculty of Education and Sport, University of the Basque Country (UPV/EHU), 01007 Vitoria, Spain; julio.calleja.gonzalez@gmail.com
^4^  Faculty of Kinesiology, University of Zagreb, 10110 Zagreb, Croatia

**Abstract**

To describe the characteristics, physiological benefits, and injury risks associated with the power clean, emphasizing the importance of proper biomechanical execution. A descriptive analysis of the power clean movement was conducted, focusing on its technical components, muscular involvement, and its role in developing physical performance. Additionally, existing considerations regarding biomechanics and injury risk were reviewed. The power clean was identified as a complex, explosive, multi-joint movement requiring coordination, strength, and precise technique. It engages multiple muscle groups across the body, including the lower limbs, core, and upper body, while significantly stimulating the central nervous system. This results in improvements in strength, power, and overall athletic performance. However, improper technique and poor biomechanics were associated with increased stress on the shoulder joint and surrounding structures, potentially leading to injuries ranging from mild muscular discomfort to severe conditions such as ligament tears, nerve impingement, or fractures. The power clean is an effective exercise for enhancing strength and explosiveness, but its technical complexity necessitates careful biomechanical execution. Proper technique is essential to minimize injury risk and maximize performance benefits, highlighting the importance of structured training and movement analysis.

**Keywords**: power clean; shoulder; injuries; analysis

**References**

1. Suchomel, T. J.; Wright, G. A.; Kernozek, T. W.; Kline, D. E. Kinetic Comparison of the Power Development between Power Clean Variations. J. Strength Cond. Res. 2014, 28 (2), 350–360. https://doi.org/10.1519/JSC.0b013e31829a36a3.
2. Burgos-Jara, C.; Cerda-Kohler, H.; Aedo-Muñoz, E.; Miarka, B. Eccentric Resistance Training: A Methodological Proposal of Eccentric Muscle Exercise Classification Based on Exercise Complexity, Training Objectives, Methods, and Intensity. Appl. Sci. 2023, 13 (13), 7969. https://doi.org/10.3390/app13137969.
3. Suchomel, T. J.; Nimphius, S.; Stone, M. H. The Importance of Muscular Strength in Athletic Performance. Sports Med. 2016, 46 (10), 1419–1449. https://doi.org/10.1007/S40279-016-0486-0.
4. Hori, N.; Newton, R. U.; Andrews, W. A.; Kawamori, N.; Mcguigan, M. R.; Nosaka, K. Does Performance of Hang Power Clean Differentiate Performance of Jumping, Sprinting, and Changing of Direction? J. Strength Cond. Res. 2008, 22 (2), 412–418. https://doi.org/10.1519/JSC.0b013e318166052b.

### 2.42. Economic Impact of Injuries in Italian Professional Football Players: A Cross-Sectional Analysis of the 2021/22 and 2022/23 Seasons

**Laura Nieto ^1^ and Pedro E. Alcaraz ^2^**

^1^  UCAM 1; lnieto@ucam.edu
^2^  UCAM, CIARD; palcaraz@ucam.edu
*  Correspondence: lnieto@ucam.edu

**Abstract**

The availability of players is key in professional football clubs as it directly impacts the team’s performance. Additionally, not having a player available for selection in a match due to injury incurs an economic cost for the club. In this regard, Lu et al. (2021) estimated that the cost of injuries in the Australian league ranges between 187,990 AUD and 332,680 AUD. Eliakim et al. (2020) calculated the cost in the Premier League to be around 45 million pounds, and Nieto et al. (2024) estimated it to be 45.2 million euros in LaLiga (Spain). This data highlights the importance of quantifying and preventing injuries in professional soccer. However, there is a lack of this kind of economics calculations in other important European Leagues, i.e., Serie A (Italy). Therefore, the present cross-sectional study for all players from Italian professional football clubs during the 2021/2022 and 2022/2023 seasons aims to: (1) present the total number of injuries and injuries by type; (2) estimate the economic cost of total injuries and by type of injury; and (3) establish a ranking that compares the relative position of each club based on the economic cost of injuries, the total number of injuries, and player absences. The economic impact of injuries was estimated by considering both player absences and the salary cost of each injured player. The main findings were: (1) The economic burden of injuries per club was substantial, amounting to €135 million in the 2021/2022 season and €92 million in the 2022/2023 season; (2) Muscular injuries accounted for the highest associated costs in both seasons (€68.2 million in 2021/2022 and €51.2 million in 2022/2023); and (3) Injuries resulted in 3124 and 2360 match absences during the 2021/2022 and 2022/2023 seasons, respectively. Therefore, in line with Jukic et al., (2021), investing in injury prevention strategies is essential for professional clubs to maintain player availability, thereby reducing costs and enhancing club performance.

**Keywords:** injuries, professional soccer, prevention, player cost, player availability

**Funding:** “This research was funded by Fundación Séneca, grant number 22134/PI/22”.

**References**

1. Lu, D., McCall, A., Jones, M., Steinweg, J., Gelis, L., Fransen, J., & Duffield, R. (2021). The financial and performance cost of injuries to teams in Australian professional soccer. *J Sci Med Sport*, *24*(5), 463–467.
2. Eliakim, E., Morgulev, E., Lidor, R., & Meckel, Y. (2020). Estimation of injury costs: financial damage of English Premier League teams’ underachievement due to injuries. *BMJ Open Sport & Exerci Med; 6*(1), e000675.
3. Jukic, I.; Calleja-González, J.; Cuzzolin, F.; Sampaio, J.; Cos, F.; Milanovic, L.; Krakan, I.; Ostojic, S.; Olmo, J.; Requena, B.; et al. (2021). The 360◦ Performance System in Team Sports: Is It Time to Design a “Personalized Jacket” for Team Sports Players? *Sports 9*(3) 40.
4. Nieto Torrejón L, Martínez-Serrano A, Villalón JM, Alcaraz PE. Economic impact of muscle injury rate and hamstring strain injuries in professional football clubs. Evidence from LaLiga. *PLoS One*. 2024 Jun 13;19(6):e0301498.

**Disclaimer/Publisher’s Note:** The statements, opinions and data contained in all publications are solely those of the individual author(s) and contributor(s) and not of MDPI and/or the editor(s). MDPI and/or the editor(s) disclaim responsibility for any injury to people or property resulting from any ideas, methods, instructions or products referred to in the content.

### 2.43. Incidence and Economic Impact of Injuries in the Main European Leagues

**Laura Nieto ^1^ and Pedro E. Alcaraz ^2^**

^1^  UCAM 1; lnieto@ucam.edu
^2^  UCAM, CIARD; palcaraz@ucam.edu
*  Correspondence: lnieto@ucam.edu

**Abstract**

Player injury incidence and availability are critical factors influencing the performance of professional football clubs. The absence of players due to injury generates significant economic costs. For instance, Nieto et al. (2024) estimated the injury-related cost in LaLiga at €45.2 million during the 2018/2019 season. Additionally, studies have linked player availability to key performance indicators such as goals scored (Eirale et al., 2013) and final league standings (Eliakim et al., 2020), emphasizing the need to quantify injury costs and develop targeted prevention strategies (Pérez et al., 2022). This cross-sectional study analyzes all players from LaLiga during the 2023/2024 season, aiming to present total injuries and injury types by player position, and to assess the economic impact of injuries by position. Injury costs were calculated based on player absences and salaries. A total of 1016 injuries were recorded, with defenders accounting for 42% (436), midfielders 38% (384), and forwards and goalkeepers less than 15%. Muscular injuries were the most common across all positions, comprising 40–50% of injuries per position—highest in defenders (50%), followed by midfielders (45%), forwards (44%), and goalkeepers (40%). Joint injuries were the second most frequent, particularly among defenders (20%) and midfielders (18%). The total injury cost for the 2023/2024 season was €178.3 million. Defenders incurred the highest costs (€83.5 million), followed by midfielders (€53.4 million), forwards (€24.3 million), and goalkeepers (€17.1 million). Notably, absences due to muscular injuries accounted for 57% of defenders’ missed matches, 50% for forwards, and 47% for midfielders. Controlling muscular injuries alone could reduce injury-related costs and absences by approximately half. The variation in injury type significance by player position underscores the necessity for position-specific prevention strategies. These findings highlight the substantial economic burden of injuries in professional football and the critical importance of tailored injury prevention to maintain player availability, reduce costs, and enhance club performance.

**Keywords**: injuries, professional soccer, prevention, player cost, player availability

**Funding**: “This research was funded by Fundación Séneca, grant number 22134/PI/22”.

**References**

1. Eirale, C., Tol, J. L., Farooq, A., Smiley, F., & Chalabi, H. (2013). Low injury rate strongly correlates with team success in Qatari professional football. *British Journal of Sports Medicine*, 47(12), 807.
2. Eliakim, E., Morgulev, E., Lidor, R., & Meckel, Y. (2020). Estimation of injury costs: financial damage of English Premier League teams’ underachievement due to injuries. *BMJ Open Sport & Exerc Med*, 6(1), e000675.
3. Pérez-Gómez, J., Adsuar, J.C., Alcaraz, P.E., Carlos-Vivas, J. (2022). Physical exercises for preventing injuries among adult male football players: A systematic review. *Journal of Sport and Health Science*, 11(1), 115–122.
4. Nieto Torrejón L, Martínez-Serrano A, Villalón JM, Alcaraz PE. Economic impact of muscle injury rate and hamstring strain injuries in professional football clubs. Evidence from LaLiga. *PLoS One*. 2024 Jun 13;19(6):e0301498.

**Disclaimer/Publisher’s Note:** The statements, opinions and data contained in all publications are solely those of the individual author(s) and contributor(s) and not of MDPI and/or the editor(s). MDPI and/or the editor(s) disclaim responsibility for any injury to people or property resulting from any ideas, methods, instructions or products referred to in the content.

### 2.44. Menstrual Status and Bone Health in Athletes from Different Sports and Non-Athletes

Gina F Øistuen ^1^, John O Osborne ^2^., Klavs Madsen ^1^ and Kristin L Jonvik ^1,^*

^1^  Department of Physical Performance, Norwegian School of Sport Sciences, Norway
^2^  Faculty of Health Sciences, The Arctic University of Norway, Tromsø, Norway
*  Correspondence: kristinlj@nih.no

**Abstract**

Relative energy deficiency in sport (REDs) is a syndrome caused by low energy availability, where known symptoms are menstrual disturbances (MD) and low bone mineral density (BMD, Z-score < −1.0). The magnitude and relation between these issues in different sports needs further attention. This study aimed to (1) investigate the prevalence of MD and low BMD between athletes from different sports and non-athletes, (2) compare BMD Z-scores at whole body, lumbar spine and total hip between females with regularly menstruation (RM), MD and females using hormonal contraceptives (HC), and (3) investigate which factors influence BMD among endurance athletes such as menstrual status, athletic caliber and sport impact. Data from 524 women were collected from ten different projects with cross-sectional data on BMD (using Dual X-ray absorptiometry), and/or menstrual status (using a questionnaire or the three step method). Participants were grouped based on menstrual status (RM, MD, and HC), sport (endurance, ball sport, technical & power, and non-athletes), and their athletic caliber (Tier 0 (sedentary) to 5 (world-class)). The endurance athletes were also grouped based on high, medium and low impact loading of their sport. Statistical analyses included chi-square testing and mixed model analyses. Among women not using HC, the prevalence of MD was 37% in endurance, 34% in ball sport, 36% in power & technical athletes and 0% in non-athletes. The prevalence of low BMD at the lumbar spine was 17% in endurance, 0% in ball sport and 8% in technical & power athletes. There were no differences in BMD at any site between RM, MD and HC (*p* > 0.05). When comparing endurance athletes (END) to non-endurance athletes and non-athletes (NON-END), END had lower BMD Z-score at lumbar spine (END: −0.0± 1.0 (N = 180) vs. NON-END: 1.2 ± 1.3 (N = 41), *p* < 0.05) and hip (END: 0.6 ± 0.9 (N = 179) vs. NON-END: 1.4 ± 1.2 (N = 41), *p* < 0.05), but not for whole body. Among END, the sport impact did not influence BMD at any site (*p* > 0.05). However, END with MD had lower BMD at the hip compared to those with RM and HC (*p* < 0.05). The prevalence of MD was higher among athletes compared to non-athletes. Endurance athletes had the lowest BMD at the lumbar spine and hip, which was even lower in endurance athletes with MD. These findings point towards an increased risk of MD and low BMD in endurance athletes. As sport impact was not the main contributor, other factors related to REDs need further attention.

**Keywords:** Relative Energy Deficiency in Sports, Menstrual disturbances, Menstrual cycle, Hormonal contraceptives, Mechanical load

**Funding:** This research was funded by Foundation Dam, grant number SDAM_HEL525787.

**Disclaimer/Publisher’s Note:** The statements, opinions and data contained in all publications are solely those of the individual author(s) and contributor(s) and not of MDPI and/or the editor(s). MDPI and/or the editor(s) disclaim responsibility for any injury to people or property resulting from any ideas, methods, instructions or products referred to in the content.

### 2.45. Tracking Health Markers in Endurance Athletes: A Cross-Sectional Study of RED-S-Related Factors by Age and Sex

Ľudmila Oreská ^1,^*, Barbora Kundeková ^1^ and Milan Sedliak ^1^

^1^  Department of Biological and Medical Sciences, Faculty of Physical Education and Sports, Bratislava, Slovakia; ludmila.oreska@uniba.sk; barbora.kundekova@uniba.sk; milan.sedliak@uniba.sk
*  Correspondence: ludmila.oreska@uniba.sk

**Abstract**

Relative Energy Deficiency in Sport (RED-S) manifests differently across the lifespan and sexes, with younger female endurance runners showing the highest apparent risk, while master male athletes exhibit relative physiological preservation. This study aimed to compare RED-S-associated anthropometric, biochemical, and strength-related parameters in young and master endurance-trained runners, highlighting age- and sex-specific vulnerabilities. Thirty-two competitive athletes were stratified into four groups: (1) Young Male Endurance Runners (YMER), (2) Master Male Endurance Runners (MMER), (3) Young Female Endurance Runners (YFER), and (4) Master Female Endurance Runners (MFER). Young adults (20–35 years) and masters (65–80 years) trained >300 min/week and regularly competed in long-distance events (≥3 years for YER, ≥25 years for MER). Body composition was assessed via dual-energy X-ray absorptiometry (DXA), evaluating skeletal muscle mass, fat mass, and bone mineral density. Fasting venous blood samples were analysed for RED-S-relevant markers, including haemoglobin, ferritin, total iron, vitamin D, transferrin, estradiol, and bioavailable testosterone. Lower-limb muscle strength was evaluated with an isometric knee dynamometer. RED-S risk screening was performed using the LEAF-Q (females) and LEAM-Q (males). Results indicated that male runners, regardless of age, exhibited significantly greater muscle mass, body weight, and bone mineral density than their female counterparts, while females demonstrated higher relative fat mass. Notably, RED-S risk—indicated by hormonal profiles, iron status, and questionnaire outcomes—was most pronounced in YFER, characterised by lower haemoglobin, ferritin, and testosterone-equivalent profiles, and highest LEAF-Q scores. In contrast, MMER exhibited stable metabolic and musculoskeletal profiles, suggesting partial protection from RED-S despite ageing. No significant differences in vitamin D3 or total iron-binding capacity were observed across groups. These findings underscore the differential expression of RED-S based on age and sex. Young female runners are at the highest risk, whereas master male athletes may retain physiological resilience. A targeted, individualised approach—combining strength and conditioning with nutritional and endocrinological oversight—is essential to reduce RED-S risk and ensure long-term health and performance sustainability in endurance athletes.

**Keywords**: endurance running; lifelong training; relative energy deficiency

**Funding**: The study was funded by the INTERREG V-A Slovakia—Austria (acronym CAA, ITMS2014+305041X157), by the Slovak Research and Development Agency (Grant no. APVV-21-0164), VEGA 0554/24, and VEGA 1/0482/23.

**References**

1. Burke, L.M.; Castell, L.; Casa, D.J.; Mountjoy, M.; Sundgot-Borgen, J.; Lundy, B.; Jeukendrup, A.; Melin, A. Mapping the complexities of Relative Energy Deficiency in Sport (REDs): Development of a physiological model by a subgroup of the International Olympic Committee (IOC) Consensus on REDs. Br. J. Sports Med. 2023, 57, 1098–1110.
2. Lee, E.C.; Fragala, M.S.; Kavouras, S.A.; Queen, R.M.; Pryor, J.L.; Casa, D.J. Biomarkers in sports and exercise: Tracking health, performance, and recovery in athletes. J. Strength Cond. Res. 2017, 31, 2920–2937.
3. Vajda, M.; Oreská, Ľ.; Černáčková, A.; Čupka, M.; Tirpáková, V.; Cvečka, J.; Sedliak, M. Aging and possible benefits or negatives of lifelong endurance running: How master male athletes differ from young athletes and elderly sedentary? Int. J. Environ. Res. Public Health 2022, 19, 13184.

**Disclaimer/Publisher’s Note:** The statements, opinions and data contained in all publications are solely those of the individual author(s) and contributor(s) and not of MDPI and/or the editor(s). MDPI and/or the editor(s) disclaim responsibility for any injury to people or property resulting from any ideas, methods, instructions or products referred to in the content.

### 2.46. Leg and Vertical Stiffness During Running with Weighted Vests at Submaximal and Maximal Aerobic Speed

Nikolaos P. Belechris ^1,^*, Gregory C. Bogdanis ^1^, Gerasimos Terzis ^1^, Polyxeni Argeitaki ^1^ and Giorgos Paradisis ^1^

^1^  School of Physical Education and Sports Science, National and Kapodistrian University of Athens, 17237 Dafne, Greece; nikosbele@phed.uoa.gr; gbogdanis@phed.uoa.gr; gterzis@phed.uoa.gr; pargeit@phed.uoa.gr; gparadi@phed.uoa.gr
*  Correspondence: nikosbele@phed.uoa.gr

**Abstract**

Leg (K_leg_) and vertical stiffness (K_vert_) are biomechanical properties that influence running performance by modulating force transmission and elastic energy return (1, 2). The spring-mass model suggests that alterations in mechanical load can affect lower-limb stiffness (3). Research has shown that running with external loads increases K_leg_ (4). However, limited evidence exists regarding how these adaptations differ under various running intensities and external loads. The present study aimed to investigate the acute effects of running at different speeds with weighted vests on K_leg_ and K_vert_ in male runners. Participants (N = 14; age = 21.2 ± 2.7 years; body mass (BM) = 67.5 ± 6.2 kg; height = 1.76 ± 0.03 m; maximal oxygen uptake (VO_2_max) = 60.38 ± 5.82 mL/kg/min; maximal aerobic speed (MAS) = 5.02 ± 0.43 m·s^−1^) completed three 6-min trials under different loading conditions: 0%, 5% and 10% BM. Each 6-min trial consisted of three 2-min stages corresponding to 60%, 80%, and 100% of MAS. During each condition, spatiotemporal parameters including contact and flight time, were recorded using a high-speed camera (300 fps). K_vert_ and K_leg_ were calculated from the spring-mass model (2). Two-way repeated measures ANOVAs were used to assess the effects of intensity and load, followed by Bonferroni-corrected pairwise comparisons using estimated marginal means. Significant effects of load were observed for peak ground reaction force, leg spring compression, K_vert_ and K_leg_ (all *p* < 0.001), but not for center of mass vertical displacement during ground contact. The ANOVAs revealed significant effects of intensity on all variables (*p* < 0.001). Pairwise comparisons revealed that running with 5% and 10% BM loads elicited significant reductions in K_vert_ relative to 0% BM at both 80% (0% BM: 27.84 N·m^−1^; 5% BM: −3.24%; 10% BM: −4.17%) and 100% MAS (0% BM: 32.88 N·m^−1^; 5% BM: −2.59%; 10% BM: −3.68%). Similar reductions were found in K_leg_ at 60% (0% BM: 9.71 kN·m^−1^; 5% BM: −6.37%; 10% BM: −9.92%), at 80% (0% BM: 9.53 kN·m^−1^; 5% BM: −7.02%; 10% BM: −11.24%), and at 100% MAS (0% BM: 10.69 kN·m^−1^; 5% BM: −4.72%; 10% BM: −6.73%), all with statistical significance (*p* < 0.01). There were no differences between the 5% and 10% BM conditions. These results demonstrate that loading with a weighted vest appears to decreases both K_leg_ and K_vert_, indicating that runners adapt to added mass by modulating stiffness. These adjustments are observed primarily between the unloaded and loaded conditions.

**Keywords:** spring-mass model; running biomechanics; leg-spring compression; vertical stiffness

**Funding:** This research received no external funding.

**References**

1. Pappas, P., Paradisis, G., Tsolakis, C., Smirniotou, A., & Morin, J. B. (2014). Reliabilities of leg and vertical stiffness during treadmill running. *Sports biomechanics*, 13(4), 391–399.
2. Morin, J., Dalleau, G., Kyrolainen, H., Jeannin, T., & Belli, A. (2005). A simple method for measuring stiffness during running. *Journal of Applied Biomechanics*, 21, 167–180.
3. Blickhan, R. (1989). The spring-mass model for running and hopping. *Journal of Biomechanics*, 22, 1217–1227.
4. Silder, A., Besier, T., & Delp, S. L. (2015). Running with a load increases leg stiffness. *Journal of biomechanics*, 48(6), 1003–1008.

**Disclaimer/Publisher’s Note:** The statements, opinions and data contained in all publications are solely those of the individual author(s) and contributor(s) and not of MDPI and/or the editor(s). MDPI and/or the editor(s) disclaim responsibility for any injury to people or property resulting from any ideas, methods, instructions or products referred to in the content.

### 2.47. Spatiotemporal Adaptations to Graded Running Intensities with Weighted Vest Loading

Giorgos P. Paradisis ^1,^*, Nikolaos Panagiotis Belechris ^1^ and Panagiotis Pappas ^1^

^1^  School of Physical Education and Sports Science, National and Kapodistrian University of Athens, 17237 Dafne, Greece; gparadi@phed.uoa.gr; nikosbele@phed.uoa.gr; ppappa@phed.uoa.gr
*  Correspondence: gparadi@phed.uoa.gr

**Abstract**

Running with external loads is very popular in many training practices and has been investigated for its impact on spatiotemporal parameters such as contact time, flight time, step length and step rate. While light loads generally have minimal effects on these variables (1), higher loads are associated with specific adjustments in running mechanics. Understanding these effects is crucial for optimizing training protocols aim to improve performance while maintaining running economy and mechanics (2). The purpose of this study was to assess the acute effect of weighted vest resistance on spatiotemporal parameters of trained runners. Participants (N = 14; age = 21.2 ± 2.7 years; body mass (BM) = 67.5 ± 6.2 kg; height = 1.76 ± 0.03 m; maximal aerobic speed (MAS) = 5.02 ± 0.43 m·s^−1^) first completed a maximal oxygen uptake assessment. Subsequently, they completed three 6-min trials under three different loading conditions: 0% BM, 5% BM and 10% BM, using a weighted vest. Each 6-min trial consisted of three 2-min stages corresponding to 60%, 80%, and 100% of MAS. During each trial condition, spatiotemporal parameters including contact time and flight time were recorded using a high-speed video camera (300 fps) while step length and step rate were subsequently calculated (3). Two-way repeated measures ANOVAs were used to assess the effects of intensity and load, followed by Bonferroni-corrected pairwise comparisons using estimated marginal means. The ANOVA revealed that running at the three different speeds significantly modulates all spatiotemporal parameters (*p* < 0.001). However, running with the three different external loads altered only contact time and flight time (*p* < 0.001). Post hoc analyses revealed that running with the loads increased contact time at 60% MAS (0% BM: 0.268 ± 0.022 s; 5% BM: +3.36%; 10% BM: +5.22%) and 80% MAS (0% BM: 0.227 ± 0.017 s; 5% BM: 3.08%; 10% BM: +5.29%), but no differences were observed between 5% BM and 10% BM at 100% MAS. Conversely, flight time decreased significantly at 60% MAS with added load (0% BM: 0.097 ± 0.032 s; 5% BM: −9.28%; 10% BM: −14.43%) yet remained unchanged at higher intensities. These results suggest that weighted vest resistance primarily modulates spatiotemporal parameters at 60% MAS, with minimal additional effects beyond 5% BM loading. Also, the 5% and 10% BM had no difference indicating that incremental loading beyond 5% BM may offer limited additional benefits for acute spatiotemporal modulation in trained runners.

**Keywords:** Gait dynamics, Load carriage
**Funding:** This research received no external funding.
**References**

1. Cartón-Llorente, A., Rubio-Peirotén, A., Cardiel-Sánchez, S., Díez-Martínez, P., Roche-Seruendo, L. E., & Jaén-Carrillo, D. (2024). Acute Effects of Overload Running on Physiological and Biomechanical Variables in Trained Trail Runners. *Applied Sciences*, *14*(21), 9853.
2. Couture, G. A., Simperingham, K. D., Cronin, J. B., Lorimer, A. V., Kilding, A. E., & Macadam, P. (2020). Effects of upper and lower body wearable resistance on spatio-temporal and kinetic parameters during running. *Sports biomechanics*, *19*(5), 633–651.
3. Pappas, P., Paradisis, G., Tsolakis, C., Smirniotou, A., & Morin, J. B. (2014). Reliabilities of leg and vertical stiffness during treadmill running. *Sports biomechanics*, 13(4), 391–399.

**Disclaimer/Publisher’s Note:** The statements, opinions and data contained in all publications are solely those of the individual author(s) and contributor(s) and not of MDPI and/or the editor(s). MDPI and/or the editor(s) disclaim responsibility for any injury to people or property resulting from any ideas, methods, instructions or products referred to in the content.

### 2.48. Shooting and Goalkeepers Response Analysis in a Professional Football League

Markel Perez-Arroniz *, Julio Calleja-González, Jon Zabala-Lili, Arkaitz Crespo and Asier Zubillaga

^1^  Department of Physical Education and Sports, Faculty of Education and Sports, University of the Basque Country, UPV/EHU, 01007 Vitoria-Gasteiz, Spain
^2^  SD Eibar, Ipurua Kalea 2, 20600, Eibar, Spain
*  Correspondence: e-mail@e-mail.com; Tel.: markelperar@gmail.com/+34 686385264

**Abstract**

The main aim of this descriptive study was to analyze the situations in which goalkeepers find themselves when receiving shots, in order to design more specific training methods, due to such a role requires a specific type of training. A total of 2238 shots have been analyzed in 15 goalkeepers belonging to 10 different teams, from 179 matches during the 2019–2020 La Liga season (First Spanish Football Division). The study was divided by two parts. In the first one, all data was analyzed with no grouping, and the second was divided in 4 groups based on final team position when the competition was finished (Top, Middle High, Middle Low and Bottom). From all the shots obtained, information has been analyzed on: temporality, the most repeated actions by the strikers and goalkeepers, separating the actions by different situations (direction, number of contacts, game situation, cross…), and the areas of the field in which the actions occur.

These are some of the conclusions we have drawn:

1. First touch shots were the most dangerous given the difficulty for defenders and goalkeepers to block and stop respectively;
2. The high percentage of deflected shots (17.6%) shows the importance of training them as they are difficult situations to analyze;
3. The more time the shooter spends preparing the shot, the more accurate it will be, but the more effective the goalkeeper’s action will also be;
4. Top teams use less set pieces and crosses, and they use to make more touches than the other groups to finish the plays.

**Keywords**: Football; goalkeeper; shot; cross; save; touch; time; field zones

**Funding**: This research received no external funding.

**Reference**

1. Perez-Arroniz M, Calleja-González J, Zabala-Lili J, Zubillaga A. The soccer goalkeeper profile: bibliographic review. The Physician and Sportsmedicine. 2023 May 4;51(3):193–202.

**Disclaimer/Publisher’s Note:** The statements, opinions and data contained in all publications are solely those of the individual author(s) and contributor(s) and not of MDPI and/or the editor(s). MDPI and/or the editor(s) disclaim responsibility for any injury to people or property resulting from any ideas, methods, instructions or products referred to in the content.

### 2.49. How to Select and Train Professional Male Soccer Goalkeepers: Expert Opinion

Markel Perez-Arroniz ^1,^*, Julio Calleja-González ^1^, Javier Fernández-Navarro ^2^ and Asier Zubillaga ^1^

^1^  Department of Physical Education and Sports, Faculty of Education and Sports, University of the Basque Country, UPV/EHU, Vitoria-Gasteiz, Spain
^2^  Science Department, Faculty of Science, Liverpool Jhon Moores University, Liverpool, United Kingdom
*  Correspondence: markelperar@gmail.com/+34 686 385 264

**Abstract**

The main aim of the present study is to provide insight into the training methods and strategies that professional coaches apply in their day-to-day work to best prepare their athletes for the highest levels of competition. Only one prior study has conducted interviews with professional goalkeeper coaches, providing valuable insights into training session design and key goalkeeper attributes(1). The opinion of 15 professional goalkeeper coaches was gathered using a semi-structured interview. Following transcription, a thematic content analysis was conducted to identify and analyze patterns within the data. From all de data obtained authors structured the results in the following sections: the evolution of the goalkeeper role, goalkeeper recruitment, specific training, individualization, conditioning training, psychological training, periodisation and session design. There are multiple approaches to selecting and training professional soccer goalkeepers, however, several key considerations emerge that any coach should take into account. (1) When integrating a new goalkeeper into a club, it is essential to assess whether their profile aligns with the club’s style of play and philosophy, (2) Training should focus both on the goalkeeper’s individual development and on preparing them for the challenges posed by upcoming opponents, (3) Coaches must recognise that they are working with individuals rather than a homogeneous group, making it essential to understand and respond to the unique needs of each goalkeeper.

**Keywords:** Soccer; goalkeeper; training; performance

**Funding:** This research received no external funding.

**Reference**

1. Otte, F. W., Millar, S. K., & Klatt, S. (2020). How does the modern football goalkeeper train?–An exploration of expert goalkeeper coaches’ skill training approaches. Journal of sports sciences, 38(11–12), 1465–1473.

**Disclaimer/Publisher’s Note:** The statements, opinions and data contained in all publications are solely those of the individual author(s) and contributor(s) and not of MDPI and/or the editor(s). MDPI and/or the editor(s) disclaim responsibility for any injury to people or property resulting from any ideas, methods, instructions or products referred to in the content.

### 2.50. Physical Activity and Polypharmacy in Long-Lived Adults

Caroline Pietta-Dias ^1^, Débora G. Marques ^1^, Nadyne Rubin ^1^, Eduarda Blanco-Rambo ^1^, Marcelo Bandeira-Guimarães, MD ^1^, Tainara Steffens, MD ^1^, Fabricio Zambom-Ferraresi ^2^, Mikel L. Sáez de Asteasu ^2^, Mikel Izquierdo ^2^ and Eduardo Lusa Cadore ^1,^*

^1^  Universidade Federal do Rio Grande do Sul, carolpieta@yahoo.com.br; dedy.marques@hotmail.com; nadynerubin@yahoo.com.br; eduardarambo@gmail.com; bgbandeira@gmail.com; tainarast@hotmail.com; edcadore@yahoo.com.br
^2^  Universidad Pública de Navarra, CIBER of Frailty and Healthy Aging, fabriciogigante@hotmail.com; mikel.lopez.saezdeasteasu@gmail.com; mikel.izquierdo@gmail.com
*  Correspondence: edcadore@yahoo.com.br, +55 (51) 99119 3651

**Abstract**

The demographic shift has been accompanied by an epidemiological transition characterized by a rise in chronic non-communicable conditions. As longevity increases, so does the prevalence of chronic degenerative diseases such as cardiovascular disease, diabetes mellitus, and hypertension, particularly among the oldest old (aged ≥ 90 years), which increase the polypharmacy prevalence in these individuals (1).Polypharmacy, commonly defined as the concurrent use of five or more medications, is a significant concern in aging populations, particularly among the oldest old. Identifying protective factors is crucial for improving care strategies (2). The purpose of this study was to examine whether physical activity level predicts polypharmacy in older nonagenarian and centenarian adults. This cross-sectional study included 124 individuals aged ≥ 90 in southern Brazil. Physical activity levels were assessed using a modified Backe Questionnaire. For the “polypharmacy”, a cutoff point of five or more medications was used for individuals over 85 years of age. Multivariate analysis was conducted using binary logistic regression, considering a significance level of *p* < 0.05 and a 95% confidence interval (CI), with the calculation of the respective odds ratio (OR) to assess whether the independent variables explain the dependent variable polypharmacy. Among the participants, 44.4% experienced polypharmacy. Lower physical activity (OR = 0.416; 95% CI: 0.195–0.886) significantly predicted polypharmacy after age adjustment. Evidence from previous studies supports this association. Polypharmacy has been linked to decreased physical performance (3), and lower physical activity levels have been associated with higher medication use among older adults (4). This suggests that physical function may influence polypharmacy risk both indirectly—by modulating disease burden—and directly, as a clinical predictor of frailty and health status. Promoting physical activity may reduce the risk of polypharmacy among long-lived older adults. From a clinical perspective, physical activity should be actively promoted as part of geriatric care plans, even in advanced age. Tailored interventions—such as supervised multicomponent exercise programs—can help preserve functional status and potentially reduce medication needs, particularly for conditions like hypertension, diabetes, and depression. 

**Keywords:** Physical function; Exercise; Aging; Nonagenarians, Centenarians, Physical activity level

**Funding:** This research was funded by National Council for Scientific and Technological Development (CNPq) and by Coordination for the Improvement of Higher Education Personnel (CAPES).

**References**

1. He, W; Goodkind, D; Kowal, P. An aging world: 2015. U.S. Census Bureau. 2016.
2. Maher, RL; Hanlon, J; Hajjar, ER. Clinical consequences of polypharmacy in elderly. Expert Opin Drug Saf. 2014, 13(1), 57–68.
3. Katsimpris, A; Linseisen, J; Meisinger, C; Volaklis, K. The association between polypharmacy and physical function in older adults: a systematic review. J Gen Intern Med. 2019, 34(9), 1865–1873.
4. Thanoo, N; et al. Polypharmacy and physical activity: associations in older adults. J Am Geriatr Soc. 2021, 69(3), 553–562.

**Disclaimer/Publisher’s Note:** The statements, opinions and data contained in all publications are solely those of the individual author(s) and contributor(s) and not of MDPI and/or the editor(s). MDPI and/or the editor(s) disclaim responsibility for any injury to people or property resulting from any ideas, methods, instructions or products referred to in the content.

### 2.51. Twenty-Four Weeks of High Load Resistance Exercise Does Not Increase Bone Mineral Density in Wheelchair Users: Results of the Bonewheel Study

Linn C Risvang *, Hannah M Rice, Julia K Baumgart, Anja MF Liljegren, Jan-Willem van Dijk, Jorunn Sundgot Borgen, Vegard Strøm, Truls Raastad and Kristin L Jonvik

^1^  Norwegian School of Sport Sciences, Norway
^2^  Norwegian University of Technology and Science, Norway
^3^  Western Norway University of Applied Science, Norway
^4^  HAN University of Applied Sciences, the Netherlands
^5^  Sunnaas Rehabilitation Hospital, Norway
*  Correspondence: linncr@nih.no

**Abstract**

Wheelchair users (WCU) are at risk of low bone mineral density (BMD, Z-score < −1.0) due to lack of skeletal stimuli. Previous interventions like electrical stimulation, vibration and standing therapy have shown minimal to no efficacy in improving BMD in populations such as those with spinal cord injury (SCI). In contrast, resistance exercise (RE) improves regional BMD in ambulant populations, but its effects in non-ambulant individuals are unknown. Thus, we aimed to investigate the effects of high load RE on BMD in WCU with non-progressive impairments. In this multi-centre 24-wk RCT, volunteers (WC use ≥50%) were randomly allocated to either upper body RE 3x/wk combined with nutrition optimisation (EX, n = 24), or nutrition optimisation only (CON, n = 21). At pre-, mid- and post-intervention, we measured LS, femoral neck (FN) and total hip BMD with dual energy x-ray absorptiometry, maximal strength, serum bone turnover markers and vitamin D, and conducted dietary recalls. The training programme consisted of four isometric and two dynamic exercises aimed at loading the LS and hip. Nutrition was optimised through supplementing vitamin D (40–120 ug/d) and calcium (500–1500 mg/d). Linear mixed model analysis was employed, adjusted for potential moderating factors. Values are frequencies or estimates and 95% CI. Forty-five WCU (SCI: 49%, cerebral palsy: 27%, other: 24%; 40% female; age: 37 ± 9 y; mass: 67 ± 19 kg; BMD Z-score < −1.0 LS: 43%, FN: 79%, hip: 76%) participated, of which 33 completed the study. Maximal isometric (+3.1 [0.8–5.4] kg), 1 RM overhead press (+2.9 [1.1–4.7] kg) and 1 RM bench press (+4.2 [1.4–7.1] kg) increased more in EX than CON (interaction *p* < 0.009). Nutrition optimisation increased serum vitamin D over time (+14 [10–18] nmol/L) and increased calcium intake over time (+179 [67–290] mg/d). No effects were found on LS BMD (group: −0.002 [−0.150 −0.146]; time: +0.002 [−0.005 0.010] g/cm^2^), and similarly no effects on FN or total hip, nor on prevalence of Z-score < −1.0 (all *p* > 0.05). Lastly, the bone formation to resorption ratio P1NP/CTX-1 increased over time in both groups (+16 [4–27]). High load RE over 24 weeks did not improve BMD, despite significant increases in maximal upper body strength. Further analyses will explore if strength development and adherence influences BMD, with findings presented at the conference. The positive bone formation-to-resorption ratio shift suggests the positive role of nutrition in bone health. Furthermore, a longer intervention period and/or larger sample size may be necessary to observe changes in BMD in this population and warrants further investigation.

**Keywords**: strength training; spinal cord injury; paraplegia; bone health

**Funding**: This research was funded by Foundation Dam, grant number 2022/FO387192.

**Disclaimer/Publisher’s Note:** The statements, opinions and data contained in all publications are solely those of the individual author(s) and contributor(s) and not of MDPI and/or the editor(s). MDPI and/or the editor(s) disclaim responsibility for any injury to people or property resulting from any ideas, methods, instructions or products referred to in the content.

### 2.52. Return to Play Time for the 30 Most Common Injuries in the UEFA Women’s Elite Club Injury Study

Vanna Roos ^1,^*, Anna Hallén ^1,2^, Katrine Okholm Kryger ^3^, Håkan Bengtsson ^1^ and Jan Ekstrand ^1^

^1^  Unit of Community Medicine, Department of Health, Medicine and Caring Sciences, Linköping University, Linköping, Sweden
^2^  Unit of Physiotherapy, Unit of Community Medicine, Department of Health, Medicine and Caring Sciences, Linköping University, Linköping, Sweden
^3^  UEFA Medical & Anti-Doping Unit, Football Division, UEFA
*  Correspondence: Vanna Roos. vanna.roos@gmail.com; +46736885186

When a football player sustains an injury, the primary question for medical staff is often, “When can they return to play?” Prognostic information is essential for the medical staff to answer such inquiries from players, coaches, managers, media, and agents. Evidence-based epidemiological data for estimating recovery times can be beneficial for medical personnel when addressing RTP inquiries. The UEFA Women’s Elite Club Injury Study (WECIS), initiated July 2018 has generated an extensive dataset database from women’s elite football clubs across Europe. This extensive dataset provides valuable information regarding the incidence and recovery duration applied for various injuries. The purpose of the study was to describe the typical duration of absence following top 30 highest incidence rate related injury types in top European women’s club football. Members of the medical staff in football clubs participating in the UEFA Women’s Elite Club Injury Study (WECIS) reported injury occurrences from a total of 72 team-seasons. Injury absence was defined as the number of days between injury occurrence and when the injured player was allowed to return to full participation by the medical team. In total, 2390 injuries were registered and included in the study. Sixty-six percent of these injuries were included among the 30 most common injury diagnoses. Most of the injuries included among the 30 most common were mild (leading to a median absence of 7 days or less, 397 cases = 23%) or moderate (median absence: 7–28 days, 69% = 1177 cases). Severe injuries (median absence ≥ 28 days) were less common (8% = 140 cases) and included only four diagnoses, all related to knee injuries. These four diagnoses, however, caused 35% of all absence during the study period. Most of the time lost due to injuries in top European women’s football teams result from moderate and severe injuries. This article provides guidelines for expected time-loss for the most common injury types in women’s professional football and will aid clinicians to make more accurate estimations of when players can be expected to return to participation following injuries.

**Keywords:** Female, severity, time loss

**Funding:** The study was supported by grants from UEFA. No competing interests declared.

**References**

1. Ekstrand J.; Krutsch W.; Spreco A. et al. Time before return to play for the most common injuries in professional football: a 16-year follow-up of the UEFA Elite Club Injury Study. *Br J Sports Med* **2020**;*54*:421–426.
2. Hallén A.; Tomás R.; Ekstrand J et al. The UEFA Women’s Elite Club Injury Study: a prospective study on 1527 injuries over four consecutive seasons 2018/19 to 2021/22 reveals thigh muscle injuries to be most common and ACL injuries most burdensome. *Br J Sports Med*. **2024**;**0**:1–9. doi:10.1136/bjsports-2023-107133.
3. Ekstrand J.; Van Zoest W.; Gauffin H. Changes in head staff members in male elite- level football teams are associated with increased hamstring injury burden for that season: the UEFA Elite Club Injury Study. BMJ Open Sport Exerc Med **2023**;*9*:e001640.
4. Ekstrand J.; Hägglund M.; Waldén M, et al. Higher level of communication between the medical staff and the performance staff is associated with a lower hamstring injury burden: a substudy on 14 teams from the UEFA Elite Club Injury Study. BMJ Open Sport & Exercise Medicine **2025**;*11*:e002182. doi:10.1136/bmjsem-2024-002182.

### 2.53. Effects of Different Types of Training on the Functional and Cognitive Capacity of Healthy Older People: Results of a Randomized Clinical Trials

Nadyne Rubin ^1,^*, Eduarda Blanco-Rambo ^1^, Caroline Rosa Muraro ^1^, Marcelo Bandeira-Guimarães ^1^, Eduardo Lusa Cadore ^1^ and Caroline Pietta-Dias ^1^

Universidade Federal do Rio Grande do Sul, nadynerubin@yhoo.com.br; eduardarambo@gmail.com; caroline.rmuraro@gmail.com; bgbandeira@gmail.com; edcadore@yahoo.com.br; carolpieta@yahoo.com.br

*  Correspondence: nadynerubin@yhoo.com.br

**Abstract**

By 2050, one-fifth of the world’s population will be over sixty years old, which will lead to an increase in chronic and degenerative diseases, resulting in frailty and dependence among those affected (1). Physical exercise has been shown to produce beneficial effects on both functional and structural parameters of the human central nervous system (2). The aim of this randomized clinical trial was to compare the effects of different training programs on the functional and cognitive performance of older people. Participants were randomized into traditional concurrent training (TCT), concurrent training composed by dance as aerobic exercise (CTD), strength training (ST) and aerobic training (AT). The training sessions occurred twice weekly for 12 weeks, with progressive intensity and volume. All training interventions were designed to reach an equivalent exercise time throughout the training period. Dual task performance (i.e., functional and cognitive) was assessed using the Timed Up and Go Cognitive test (TUGcog). Cognitive function was also assessed by Mini Mental State Examination (MMSE) and Digit Span test of the Wechsler Memory Scale. Main effects of time, group and time x group interaction were analyzed using Generalized Estimating Equations, with LSD post-hoc tests for pairwise comparisons. Significance was accepted when *p* < 0.05, and results are presented as absolute delta values. The analyzes were carried out using SPSS software. The sample consisted of 41 participants (76% women; 68.8 ± 4.2 years old). After 12 weeks of intervention, there was significant time vs. group interaction in MEEM (*p* = 0.002), which the groups presented an average increase by: TCT = 2.27 points (*p* < 0.001), TCD = 0.91 points (*p* = 0.015), while remained groups did not present significant changes (*p* > 0.05). The TUGcog test showed significan main effect of time (*p* = 0.001), with a mean reduction of 0.6, 0.9, 0.5 and 1.4 s in the respective groups TCT, TCD, ST and AT, without significant time vs. group interaction. Considering the Digit Span test, there was a significant group effect (*p* = 0.009), with a significant difference between TCT and AT (*p* = 0.010), as well as between ST and AT (*p* = 0.005). Global cognitive capacity increased only in the concurrent groups, while all training groups induced improvements in the working memory. These findings suggest that different exercise interventions can potentially induce benefits in cognitive function in older adults.

**Keywords**: Aging, Cognition, Strength training, Endurance training, Combined training, Multicomponent training, Dance

**Funding**: This research was funded by National Council for Scientific and Technological Development (CNPq) and by Coordination for the Improvement of Higher Education Personnel (CAPES).

**References**

1. Albala, C.; Sánchez, H.; Lera, L.; Angel, B.; Cea, X. Efecto sobre la salud de las desigualdades socioeconómicas en el adulto mayor: Resultados basales del estudio expectativa de vida saludable y discapacidad relacionada con la obesidad (Alexandros). Rev. Médica De Chile 2011, 139, 1276–1285.
2. Hötting, BR; Holzschneider, K; Kauschke, K; Schmidt, T; Braumann, KM; Röder, B; Differential cognitive effects of cycling versus stretching/coordination training in Middle-age adults. Health Psychology, 2012, Vol.31, No. 2, 145–155.

**Disclaimer/Publisher’s Note:** The statements, opinions and data contained in all publications are solely those of the individual author(s) and contributor(s) and not of MDPI and/or the editor(s). MDPI and/or the editor(s) disclaim responsibility for any injury to people or property resulting from any ideas, methods, instructions or products referred to in the content.

### 2.54. Effects of Concurrent Training Including High-Speed Strength Training on Neuromuscular Adaptations in Older Adults: A Comparative Study

Nadyne Rubin ^1,^*, Eduarda Blanco-Rambo ^1^, Caroline Rosa Muraro ^1^, Marcelo Bandeira-Guimarães ^1^, Antenor Barbosa Calandrini ^1^, Eduardo Lusa Cadore ^1^ and Caroline Pietta-Dias ^1^

Universidade Federal do Rio Grande do Sul, nadynerubin@yhoo.com.br; eduardarambo@gmail.com; caroline.rmuraro@gmail.com; bgbandeira@gmail.com; antenorcalandrini@gmail.com; edcadore@yahoo.com.br; carolpieta@yahoo.com.br

*  Correspondence: nadynerubin@yhoo.com.br

**Abstract**

Combined strength and endurance training (i.e., concurrent training, CT) has been recommended to counteract neuromuscular and cardiovascular functions declines associated with aging (1; 2; 3). Although concurrent training has been investigated in older populations, the effects of such an exercise intervention that includes high-speed strength training (ST) remain scarce. The aim of this study was to investigate neuromuscular adaptations induced by CT composed by high-speed ST compared to high-speed ST in healthy older adults. Participants were randomly allocated into CT group (CTG) and ST group (STG). The training sessions occurred twice weekly during 12 weeks, with progressive intensity and volume. Maximal strength was assessed by one-repetition maximum test (1 RM), and maximal power output at 30% (MP30%) of 1 RM were determined in the bilateral knee extension exercise. Functional capacity and cardiorespiratory fitness were assessed using the sit-to-stand test (STS) and 6-min walking (6 MWT), respectively. Main effects of time, group and time x group interaction were analyzed using Generalized Estimating Equations, with LSD post-hoc tests for pairwise comparisons (significant at *p* < 0.05). Results are presented as absolute delta values. The analyzes were carried out using SPSS software. The sample consisted of 31 participants (68 ± 4.2 years old). After 12 weeks of intervention, there was a significant time effect (*p* < 0.001) without significant time vs. group interaction. Improvements in STS (overall D = 2.2 s) were in average 2.4 s for CTG, and 2.1 s for STG. For the 6 MWT, there was a significant time effect (*p* < 0.001), without time vs. group interaction (overall average increase was 17.5 m). CTG group increased on average 30.6 m, whereas STG increased by 4.5 m. Regarding 1 RM, there was a significant time effect (*p* < 0.001) with overall average increase of 9.8 kg. CTG showed an improvement of 13.0 kg, while ST showed an increase of on average 6.6 kg. For MP30%, there was a significant time effect (*p* < 0.001) with an average overall increase of 32.9 W. CTG increased on average 35.8 W, and STG increased by 30.0 W. The present findings showed that CT including high-speed ST does not impair neuromuscular and functional gains compared to ST alone, as both groups demonstrated significant improvements in maximal strength, MP30% and STS performance. The magnitude of 6 MWT improvement was greater in CTG compared to STG, although this difference was not statistically significant.

**Keywords:** Concurrent training; power training; aging; muscle strength

**Funding:** This research was funded by National Council for Scientific and Technological Development (CNPq) and by Coordination for the Improvement of Higher Education Personnel (CAPES).

**References**

1. Cadore, E. L., Izquierdo, M., Pinto, S. S., Alberton, C. L., Pinto, R. S., Baroni, B. M., Vaz, M. A., Lanferdini, F. J., Radaelli, R., González-Izal, M., Bottaro, M., & Kruel, L. F. M. (2012). Neuromuscular adaptations to concurrent training in the elderly: effects of intrasession exercise sequence. AGE, 35(3), 891–903. https://doi.org/10.1007/s11357-012-9405-y.
2. Müller, D. C., Boeno, F. P., Izquierdo, M., Aagaard, P., Teodoro, J. L., Grazioli, R., Cunha, G., Ferrari, R., Saez de Asteasu, M. L., Pinto, R. S., & Cadore, E. L. (2021). Effects of high-intensity interval training combined with traditional strength or power training on functionality and physical fitness in healthy older men: A randomized controlled trial. Experimental Gerontology, 149, 111321. https://doi.org/10.1016/j.exger.2021.111321.
3. Müller, D. C., Izquierdo, M., Boeno, F. P., Aagaard, P., Teodoro, J. L., Grazioli, R., Radaelli, R., Bayer, H., Neske, R., Pinto, R. S., & Cadore, E. L. (2020). Adaptations in mechanical muscle function, muscle morphology, and aerobic power to high-intensity endurance training combined with either traditional or power strength training in older adults: a randomized clinical trial. European Journal of Applied Physiology, 120(5). https://doi.org/10.1007/s00421-020-04355-z.

**Disclaimer/Publisher’s Note:** The statements, opinions and data contained in all publications are solely those of the individual author(s) and contributor(s) and not of MDPI and/or the editor(s). MDPI and/or the editor(s) disclaim responsibility for any injury to people or property resulting from any ideas, methods, instructions or products referred to in the content.

### 2.55. Workload Compensation Strategy in Non-Starters Soccer Players

Giovanni Sala Ramirez ^1,^*, Laura Alberti Zandavalli ^1^, Rafael Grazioli ^2^, Fábio Yuzo Nakamura ^3^ and Eduardo Lusa Cadore ^1^

^1^  Universidade Federal do Rio Grande do Sul; gbsrmz@gmail.com; laurazandavalli@outlook.com; edcadore@yahoo.com.br
^2^  Universidade São Francisco; rafael_grazioli@hotmail.com
^3^  Universidade da Maia; fnakamura@umaia.pt
*  Correspondence: gbsrmz@gmail.com

**Abstract**

The evolution of soccer in recent years has led Brazilian teams to compete 50 to 70 matches per season, resulting in a congested calendar and limited recovery [1]. So, starting players have a higher workload compared to non-starters [2]. Physical trainers and coaches have incorporated compensatory training for non-starters to mitigate these differences [3,4]. However, the literature lacks information on the real magnitude of compensation effects. Therefore, the aim of this study is to compare the effects of compensatory training on the relative accumulation of external load performed between non-starters (not participating in the match) and starters. The study included 43 professional soccer players who competed in nationally and internationally. GPS metrics were recorded during 68 matches played in the post-pandemic period. The athletes had a mean age of 25 ± 4 years, body mass of 77.1 ± 7.1 kg and height of 178 ± 7.06 cm. The participants were divided into two groups: starters players (who played for >60 min); and, non-starters players (who did not participate in the match). The metrics analyzed included distance per minute at high intensity (>19 km/h) and intense actions per minute (sum of accelerations and decelerations > 3 m/s^2^). Relative metrics based on minutes of activity were used. Non-starters players performed a post-match compensatory training, which consisted of high-intensity interval training with an average of 925 m covered. Generalized Estimating Equations (GEE) were used to compare the groups and the effects of compensation. In case of interaction effects, post hoc tests with LSD were applied. The significance level adopted was *p* < 0.05. The distance covered per minute at high intensity was lower for non-starters in the no-compensation compared to starters (non-starters: 1.32 [0.90, 1.75]; starters: 5.74 [4.95, 6.52]; *p* < 0.000). However, no significant differences were observed when non-starters performed compensation (non-starters: 7.4 [5.46, 9.50]; starters: 5.54 [4.63, 6.46]; *p* = 0.087). Regarding intense actions per minute, there was significant main effect for group, compensation or group x compensation interaction (*p* > 0.05) (non-starters: 0.64 [0.53, 0.75]; starters: 0.45 [0.41, 0.49]; *p* = 0.651). In summary, non-starters players who do not participate in matches have a lower external load compared to starters. Coaches should monitor the balance of workload among players. Compensatory strategies can be highly effective in equalizing these differences, minimizing fitness losses and the risk of injury, especially during congested periods with limited training time.

**Keywords**: performance; compensation strategy; soccer

**Funding**: This research received no external funding.

**References**

1. Mohr, M.; Draganidis, D.; Chatzinikolaou, A.; Barbero-Álvarez, J.C.; Castagna, C.; Douroudos, I.; Avloniti, A.; Margeli, A.; Papassotiriou, I.; Flouris, A.D.; Jamurtas, A.Z.; Krustrup, P.; Fatouros, I.G. Muscle damage, inflammatory, immune and performance responses to three football games in 1 week in competitive male players. Eur J Appl Physiol 2016, 116, 179–193.
2. Casamichana, D.; Martín-García, A.; Díaz, A.G.; Bradley, P.S.; Castellano J. Accumulative weekly load in a professional football team: with special reference to match playing time and game position. Biology of Sport 2022, 39, 115–124.
3. Hills, S.P.; Barwood, M.J.; Radcliffe, J.N.; Cooke, C.B.; Kilduff, L.P.; Cook, C.J.; Russell, M. Profiling the Responses of Soccer Substitutes: A Review of Current Literature. Sports Med 2018, 48, 2255–2269.
4. Dalen, T.; Lorås, H. Monitoring Training and Match Physical Load in Junior Soccer Players: Starters versus Substitutes. Sports (Basel) 2019, 7, 70.

**Disclaimer/Publisher’s Note:** The statements, opinions and data contained in all publications are solely those of the individual author(s) and contributor(s) and not of MDPI and/or the editor(s). MDPI and/or the editor(s) disclaim responsibility for any injury to people or property resulting from any ideas, methods, instructions or products referred to in the content.

### 2.56. Comparison of Neural Determinants of Force Steadiness Improvements Following 8 Weeks of Resistance Training in Young and Older Adults

Alessandro Sampieri ^1^, Gioi Spinello ^1^, Antonio Paoli ^1^, Tatiana Moro ^1^ and Andrea Casolo ^1,^*

^1^  Department of Biomedical Sciences, University of Padua, Italy
*  Correspondence: andrea.casolo@unipd.it

**Abstract**

Force steadiness (FS) refers to the ability to maintain a constant force output and is critical for balance and the execution of precise motor tasks. Fluctuations in force during steady isometric contractions are largely attributed to oscillations in common synaptic input to motor neurons, which modulate motor unit (MU) discharge variability. FS declines with aging but improves with resistance training (RT). However, the neural mechanisms underlying these adaptations and potential age-related differences remain unclear. Thus, we investigated the effects of RT on FS, measured as the coefficient of variation of force (CoV_F), and its neural determinants, including discharge rate (DR) variability assessed by the coefficient of variation of interspike intervals (CoV_ISI), in young (Y) and older (O) adults. Nine Y (23 ± 3 yrs) and 11 O (73 ± 6 yrs) were assessed before (T0) and after completing an 8-week dynamic and progressive RT program (T8). DR and CoV_ISI of active MU were measured in the vastus lateralis using high-density electromyography (HDsEMG) during maximal voluntary force (MVF) and 30 s steady contractions at 10% and 30% MVF. Group differences in MVF and CoV_F were assessed using a mixed two-way ANOVA. MU discharge properties were analyzed using generalized linear mixed models, and associations between Cov_F and CoV_ISI were examined using repeated measures correlation. A total of 839 MU were identified across contraction intensities and age groups. No significant interaction effects were found between time (T0 vs. T8) and group (Y vs. O) for any outcome. MVF increased in both groups (+19%; time effect: *p* < 0.001). Force steadiness improved after training, as evidenced by reductions in CoV_F of 7% at 10% MVF (time effect: *p* = 0.002) and 10% at 30% MVF (time effect: *p* = 0.002), independent of age. DR increased significantly only at 30% MVF (+5%; time effect: *p* < 0.01). CoV_ISI decreased by 7% at 10% MVF (time effect: *p* = 0.020) and by 6% at 30% MVF, although the latter did not reach statistical significance (time effect: *p* = 0.06). Notably, changes in CoV_F and CoV_ISI were positively correlated at both 10% MVF (r_rm_ = 0.68, *p* < 0.001) and 30% MVF (r_rm_ = 0.63, *p* = 0.01). Our findings indicate that both Y and O exhibit comparable improvements in FS and MU DR variability following 8 weeks of RT, suggesting that age does not impair the capacity for neural adaptation during isometric tasks. Furthermore, the observed association between CoV_ISI and CoV_F supports the notion that MU DR variability may be a key neural mechanism contributing to enhanced FS.

**Keywords:** force steadiness, motor unit; resistance training; aging

**References**

1. Enoka R.M; Farina D. Force Steadiness: From Motor Units to Voluntary Actions. *Physiology (Bethesda)*. 2021.
2. Vila-Cha C; Falla Deborah. Strength training, but not endurance training, reduces motor unit discharge rate variability. *J Electromyogr Kinesiol*. 2016.

**Disclaimer/Publisher’s Note:** The statements, opinions and data contained in all publications are solely those of the individual author(s) and contributor(s) and not of MDPI and/or the editor(s). MDPI and/or the editor(s) disclaim responsibility for any injury to people or property resulting from any ideas, methods, instructions or products referred to in the content.

### 2.57. Mechanical Characteristics of Plyometric Exercises Performed with and Without Footwear in Football Players: An Experimental Study

Gastón R. Sanchez-Ottado ^1^, Konstantinos Spyrou ^1,2,^* and Tomás T. Freitas ^1,2,3^

^1^  UCAM Research Center for High Performance Sport, Catholic University of Murcia, Murcia, Spain
^2^  Facultad de Deporte, UCAM Universidad Católica de Murcia, Murcia, Spain
^3^  NAR—Nucleus of High Performance in Sport, Saõ Paulo, Brazil
*  Correspondence: kspyrou@ucam.edu

**Abstract**

Football is characterized as a high-intensity intermittent sport involving brief and explosive actions (1). In this context, plyometric training is widely used to enhance neuromuscular performance (2, 3). However, there is limited evidence regarding how footwear conditions—sports shoes versus barefoot—affect the mechanical characteristics of these exercises (4). Since footwear can alter the behavior of the musculoskeletal system, it becomes relevant to understand its impact on jump tests used for training monitoring. To analyze the acute effects of wearing sports shoes versus barefoot on performance variables during the countermovement jump (CMJ) and drop jumps (DJ8 cm, DJ32 cm, DJ52) in young football players. A randomized crossover experimental design was used. Twenty-one male football players (academy level, professional club, Montevideo) performed CMJ and DJ tests under two conditions: barefoot and sports shoes. Each participant visited the lab on a single occasion. The protocol included a standardized warm-up and two consecutive attempts for each condition and type of jump. Variables were measured using Ivolution force plates and analyzed with Valkyria Trainer software (version 6, Sunchales, Argentina). A paired sample *t*-test was used (significance level *p* < 0.05), and effect sizes were calculated. In the CMJ, significant differences favoring the shoe condition were found in peak landing power (*p* = 0.008), jump height (*p* < 0.001), peak velocity (*p* < 0.001), and landing stiffness (*p* < 0.001). In DJs, significant differences were observed based on drop height: DJ8 and DJ32 showed better results than DJ52 cm in jump height, RSI, and contact time. No differences were found between DJ8 and DJ32. Regarding peak contact force, differences were found both by jump height and footwear condition: force increased with higher drop heights and was also slightly higher when wearing shoes. Results suggest that wearing sports shoes positively influences performance and force absorption variables during the CMJ. Additionally, in DJs, drop height has a substantial impact on performance, with lower heights (8 and 32 cm) being more efficient than 52 cm in terms of stretch-shortening cycle utilization. Footwear also showed a moderate effect on peak contact force. These findings may guide the design and progression of plyometric programs, especially for youth populations, by considering both surface condition and jump height.

**Keywords:** CMJ; drop jump; barefoot

**Funding:** This research received no external funding.

**References**

1. Stølen T, Chamari K, Castagna C, Wisløff U. Physiology of Soccer. Sports Med. **2005**;35(6):501–36.
2. Ramírez-delaCruz M, Bravo-Sánchez A, Esteban-García P, Jiménez F, Abián-Vicén J. Effects of Plyometric Training on Lower Body Muscle Architecture, Tendon Structure, Stiffness and Physical Performance: A Systematic Review and Meta-analysis. Sports Med Open. **2022**; 8(1):40.
3. Aloui G, Souhail H, Hayes LD, Bouhafs EG, Chelly MS, Schwesig R. Effects of Combined Plyometric and Short Sprints Training on Athletic Performance of Male U19 Soccer Players. Front Psychol. **2021**; 12:714016.
4. Sanchez-Ottado G, Spyrou K, Pereira L, et al. Effects of plyometric training performed on different surfaces and with different types of footwear on the neuromuscular performance of team-sport athletes: A systematic review. Biol Sport. **2025**; 42(4):107–123.

**Disclaimer/Publisher’s Note:** The statements, opinions and data contained in all publications are solely those of the individual author(s) and contributor(s) and not of MDPI and/or the editor(s). MDPI and/or the editor(s) disclaim responsibility for any injury to people or property resulting from any ideas, methods, instructions or products referred to in the content.

### 2.58. Agreeing on One Intensity Scale: The Norwegian Endurance Sport Experience

Siren Amelia Seiler-Viken ^1,^*, Fredrik Mentzoni ^2^, Stephen Seiler ^3^, Sondre Skarli ^2^ and Thomas Losnegard ^1,2^

^1^  Department of Physical Performance, Norwegian School of Sport Sciences, Sognsveien 220, 0863, Oslo, Norway; sirenas@nih.no; thomasl@nih.no
^2^  The Norwegian Olympic Sports Center, The Norwegian Olympic and Paralympic Committee and Confederation of Sports, Sognsveien 73, 0854, Oslo, Norway; Fredrik.Mentzoni@olympiatoppen.no; Sondre.Skarli@olympiatoppen.no
^3^  Faculty of Health and Sport Sciences, University of Agder, Universitetsveien 25, 4630, Kristiansand S, Norway; stephen.seiler@uia.no
*  Correspondence: sirenas@nih.no; Tel.: +4745068974

**Abstract**

Various intensity zone scales have been proposed and discussed in the literature [1,2,3,4], yet few investigations have explored how they are used in practice. Clarifying how intensity zones are defined and used seems an important step towards more effective communication between researchers and practitioners. This study investigated potential differences in the adoption and use of endurance intensity zone scales across geographic regions, sports disciplines, practitioner roles, and performance levels. A total of 778 endurance practitioners completed a survey, with 710 responses analyzed for the number of intensity zones used, and 298 responses analyzed for lower percentage of maximum heart rate (%HR_max_) demarcations within a 5-zone scale. A 5-zone scale (47%) was most frequently used. Norwegian respondents were 2.7 times more likely to use a 5-zone scale than all other regions combined. The lower limits of Zones 2–3 differed across regions, and Zones 1–3 across sports, particularly between running and cycling. Norwegian respondents reported the highest mean %HR_max_ lower limits of Zones 2 and 3. Cyclists self-selected the lowest mean %HR_max_ lower limits of Zones 1, 2, and 3. No differences were observed in zone demarcations across roles or performance levels. The results suggest that the standardized intensity scale developed for Norwegian elite endurance athletes has been broadly adopted across sports and performance levels. The widespread use of a single intensity scale is speculated to improve communication across sports. Cyclists may self-select lower zone demarcations in the low-intensity domain by virtue of longer session durations and lower mechanical output, contributing to the popularity of “Zone 2 training” in cycling.

**Keywords:** Endurance sports, Training intensity, Intensity zones, Heart rate, Zone 2 training

**Funding:** This research received no external funding.

**References**

1. Steinacker, J.M.; Lormes, W.; Lehmann, M.; Altenburg, D. Training of rowers before world championships. *Med. Sci. Sports Exerc.* **1998**, *30*, 1158–1163.
2. Meyer, T.; Lucía, A.; Earnest, C.P.; Kindermann, W. A conceptual framework for performance diagnosis and training prescription from submaximal gas exchange parameters–Theory and application. *Int. J. Sports Med*. **2005**, *26*(Suppl 1), S38–S48.
3. Billat, V.L.; Lepretre, P.M.; Heugas, A.M.; Laurence, M.H.; Salim, D.; Koralsztein, J.P. Training and bioenergetic characteristics in elite male and female Kenyan runners. *Med. Sci. Sports Exerc.* **2003**, *35*, 297–304.
4. Seiler, S. What is best practice for training intensity and duration distribution in endurance athletes? *Int. J. Sports Physiol. Perform.* **2010**, *5*, 276–291.

**Disclaimer/Publisher’s Note:** The statements, opinions and data contained in all publications are solely those of the individual author(s) and contributor(s) and not of MDPI and/or the editor(s). MDPI and/or the editor(s) disclaim responsibility for any injury to people or property resulting from any ideas, methods, instructions or products referred to in the content.

### 2.59. Exploring the Changes of Direction Profile of Elite Futsal Players During Official Matches

Konstantinos Spyrou ^1,2,3,^*, Maziar J. Hammad ^1,2^, João N. Ribeiro ^4,5,6^, Pedro E. Alcaraz ^1,2,3^, Bruno Travassos ^4,7,8^ and Tomás T. Freitas ^1,2,3,9^

^1^  UCAM Research Center for High Performance Sport, UCAM Universidad Católica de Murcia, 30107 Murcia, Spain
^2^  Facultad de Deporte, UCAM Universidad Católica de Murcia, 30107 Murcia, Spain
^3^  Strength & Conditioning Society, 30008 Murcia, Spain
^4^  Department of Sport Sciences, Universidade da Beira Interior, Covilhã, Portugal
^5^  School of Education, Communication and Sports, Polytechnic Institute of Guarda, Guarda, Portugal
^6^  SPRINT Sport Physical Activity and Health Research & Innovation Center, Rio Maior, Portugal
^7^  Research Center in Sports Sciences, Health Sciences and Human Development (CIDESD), Covilhã, Portugal
^8^  Portugal Football School, Portuguese Football Federation, Oeiras, Portugal
^9^  NAR-Nucleus of High Performance in Sport, São Paulo 04753-060, Brazil

**Abstract**

Futsal is considered as a high-intensity intermittent team-sport, with the high number of accelerations, decelerations and changes of direction (COD) (1, 2). The aim of this study is to explore the specific angles of the COD in professional futsal players during official matches. Thirteen elite male futsal players (age: 26.7 ± 3.1 years; body mass: 74.7 ± 5.9 kg; height: 1.78 ± 0.06 m) from the same team competing in the 1st Division of Spain were monitored for this study during 37 official games. The angle direction data collected from both a tri-axial accelerometer and gyroscope to graphically evaluate the magnitude of movement an athlete makes and this allows the user to track athlete performance without a GPS signal and at a higher sampling frequency (100 Hz). All the actions were considering without considering the intensity that performed them. A statistical package JASP Team (2024), JASP (Version 0.18.3) [Computer software] was used for the analysis. A pared-sample *T*-test was used to compare the differences among angles. Futsal players performed significantly more total action in 30°, 60°, 90°, 120°, 150°, and 180° when compared to 0° angle (meaning linear acceleration), (*p* = 0.001; ES: 0.4–2.6). Players performed significantly more action in 60°, 90°, and 120° (*p* = 0.001; ES: 1.07–1.36) but significant less actions on 150° and 180° when compared to 30° (*p* = 0.001, ES: 1.43–1.52). Non-significant differences were found among 60°, 90°, and 120° (*p* = 0.076–0.529; ES: 0.06–0.01), but they performed significant less actions 150° and 180° when compared to 60°, 90° and 120° (*p* = 0.001; ES: 1.98–2.43). Non-significant differences were found between 150° and 180° of COD (*p* = 0.165, ES: 0.08). In summary, from the current results seems these players performed higher number of COD on angles 60°, 90°, and 180° when compared to the others angles, such as 0°, 30°, 150° and 180°. From practical application, futsal coaches are advised to train player’s COD capacity on different angles, highlighting those that are perform mostly during the match.

**Keywords:** five-a-side soccer; performance; team-sports

**Funding:** This research received no external funding.

**References**

1. Spyrou, K., Freitas, T. T., Marín-Cascales, E., & Alcaraz, P. E. (2020). Physical and physiological match-play demands and player characteristics in futsal: a systematic review. Frontiers in psychology, 11, 569897.
2. Ribeiro, J. N., Gonçalves, B., Coutinho, D., Brito, J., Sampaio, J., & Travassos, B. (2020). Activity profile and physical performance of match play in elite futsal players. Frontiers in psychology, 11, 1709.

**Disclaimer/Publisher’s Note:** The statements, opinions and data contained in all publications are solely those of the individual author(s) and contributor(s) and not of MDPI and/or the editor(s). MDPI and/or the editor(s) disclaim responsibility for any injury to people or property resulting from any ideas, methods, instructions or products referred to in the content.

### 2.60. Effects of Short-Term Testosterone Administration and Resistance Training on Body Composition and Strength in Healthy Men Aged 55–70: A Double-Blind, Randomised, Placebo-Controlled Trial

Daniel Tømmerbakke ^1^, Ingrid Hansen ^1^, Håkon Biserød Vengnes ^1^, Jonas Maximilian Kanden ^1^, Øyvind Skattebo ^1^, Olivier R. Seynnes ^1^, Gøran Paulsen ^1^, Julie Berge Mæhlum ^2^, Per Medbøe Thorsby ^2^, Daniel C. Turner ^1^, Truls Raastad ^1^ and Adam P. Sharples ^1,^*

^1^  Institute for Physical Performance, Norwegian School of Sport Sciences, Oslo, Norway
^2^  Department of Biochemical endocrinology and metabolism, Oslo University Hospital, Oslo, Norway
*  Correspondence: adams@nih.no

**Abstract**

A primary hallmark of ageing is the reduction in muscle size and function, impairing physical function and quality of life in older adults, a disorder known as sarcopenia. While resistance training (RT) remains the cornerstone to offset sarcopenia, its efficacy is often limited by anabolic resistance, partly due to age-related declines in anabolic hormones. In this double-blind, randomised, placebo-controlled trial, we investigated whether short-term testosterone administration enhances RT-induced effects on body composition and muscle strength in healthy men aged 55–70 years. Forty-two participants will be randomised into four groups: RT plus testosterone (T + RT), RT plus placebo (P + RT), testosterone alone (T), or placebo alone (P). Here we report preliminary results on the first 18 participants. Testosterone undecanoate was administered intramuscularly (1000 mg/4 mL at baseline and 500 mg/2 mL at week 3); placebo groups received saline injections. RT groups completed supervised whole-body resistance training three times per week (3–4 sets of 6–12 repetitions per exercise). Assessments included dual-energy X-ray absorptiometry (DXA) for body composition, knee extensor strength tests (isometric at 60° and isokinetic at 180°/s), and serum testosterone levels. Serum testosterone increased above physiological levels by week 1 in T + RT (40.2 ± 7.1 nmol/L, *p* = 0.010) and T (35.7 ± 8.3 nmol/L, *p* = 0.014), remained elevated in both groups at week 4 (T + RT: 40.0 ± 9.7 nmol/L, *p* = 0.013), and returned to baseline by week 10. T + RT significantly increased lean body mass (+5.2%, *p* = 0.002) and decreased fat percentage (−2.0%, *p* = 0.004). P + RT showed no significant changes in lean body mass (+0.9%, *p* = 0.573) or fat percentage (−0.1%, *p* = 0.845). The T and P groups did not differ from baseline. Arm lean mass increased in T + RT (+603 g, *p* = 0.002) and P + RT (+304 g, *p* = 0.010), but only T + RT resulted in significant increases in leg (+916 g, *p* = 0.012) and trunk (+1776 g, *p* = 0.003) lean mass. Mean training volume and the heaviest loads lifted in training sets increased across RT groups. However, knee extensor strength did not improve significantly in any group. In conclusion, short-term supraphysiological testosterone administration combined with RT significantly enhances whole-body and regional lean mass gains in healthy men aged 55–70 years, beyond those achieved with RT or testosterone alone. These preliminary findings suggest that testosterone adjunct to RT could be a time-efficient strategy to counteract age-related anabolic resistance and improve body composition. Additional findings from the full sample, including muscle thickness and muscle performance outcomes, will be presented at the conference.

**Keywords**: testosterone supplementation; resistance training; lean body mass; anabolic resistance; sarcopenia; aging

**Funding**: This research was funded by The Norwegian School of Sport Sciences.

**References**

1. Cruz-Jentoft, A. J. et al. Sarcopenia: revised European consensus on definition and diagnosis. Age Ageing 48, 16–31 (2019).
2. Gharahdaghi, N. et al. Testosterone therapy induces molecular programming augmenting physiological adaptations to resistance exercise in older men. J. Cachexia Sarcopenia Muscle 10, 1276–1294 (2019).

**Disclaimer/Publisher’s Note:** The statements, opinions and data contained in all publications are solely those of the individual author(s) and contributor(s) and not of MDPI and/or the editor(s). MDPI and/or the editor(s) disclaim responsibility for any injury to people or property resulting from any ideas, methods, instructions or products referred to in the content.

### 2.61. Lower-Body Adaptations to Three Different Strength Training Methods

Glenn Trane *, Olav Melhus Gomo, Stine Pedersen, Jan Helgerud and Runar Jakobsen Unhjem

Faculty of Education and Arts, Nord University, Bodø, Norway

Faculty of Medicine and Health Sciences, Norwegian University of Science and Technology, Trondheim

Treningsklinikken, Medical Rehabilitation Clinic, Trondheim, Norway

*  Correspondence: glenn.trane@nord.no; Tel.: +47 95220052

**Abstract**

Within the same sample as the upper-body study of Trane et al. (2025), we examined squat and squat jump (SJ) adaptations to lower-body maximal strength training (MST), hypertrophy training (HT) and explosive strength training (EST). MST and EST were equated for workload, while HT was conducted at a higher volume. Methods: 58 moderately trained individuals (29 males and 29 females) were divided into four training groups: (1) MST—performing 4 sets of 4 repetitions of squats at ≥85% of one repetition maximum (1 RM), (2) HT—performing 3 sets of 8–12 repetitions of squats at 70–80% of 1 RM, (3) EST—performing 4 sets of 6–7 unloaded SJs, (4) a Control group (CON). MST, HT and EST groups trained three times per week for eight weeks, with two sessions including 6 × 25-m sprints. The CON groups performed two sprint sessions per week. We evaluated squat maximal strength, the rate of force development (RFD) during SJs at 50%, 30% and 0% of squat 1 RM (RFDSJ50%, RFDSJ30% and RFDSJ0%), as well as the height of SJs and counter movement jumps (CMJs) and leg lean mass. Results: MST and HT increased squat 1 RM by 18 kg each, significantly more than EST (9 kg) (all *p* ≤ 0.01) and CON (all *p* ≤ 0.001). MST and HT improved RFDSJ50% by 195 N ∙ s^−1^ and 177 N ∙ s^−1^ (all *p* ≤ 0.001), RFDSJ30% by 203 N ∙ s^−1^ and 224 N ∙ s^−1^ (*p* ≤ 0.001–0.01) and RFDSJ0% by 224 N ∙ s^−1^ and 392 N ∙ s^−1^ (*p* ≤ 0.01–0.05), whereas EST and CON showed no improvements. EST increased SJ height by 12% and CMJ height by 8% (all *p* ≤ 0.001), comparable to the improvements seen with MST (12% and 11%, all *p* ≤ 0.001) and HT (10% and 7%, all *p* ≤0.01). The improvements in squat 1 RM were correlated with changes in SJ and CMJ height (r = 0.4 and r = 0.6, all *p* ≤ 0.001). Conclusion: MST and HT resulted in improvements across all assessed squat and jumping tests, while EST primarily improved unloaded jump performance. Improved jumping performance following MST and HT appear to be largely dependent on increases in muscle strength, whereas for EST, coordinative adaptations may play a more significant role.

**Keywords**: Counter movement jump (CMJ), Dual x-ray absorptiometry (DXA), Explosive strength training (EST), Hypertrophy training (HT), Maximal strength training (MST), Rate of force development (RFD), squat, squat jump (SJ)

**Funding**: This research was funded by Nord university.

**Reference**

1. Trane, G.; Pedersen, S.; Mehus, H.A.; Helgerud, J.; Unhjem, R.J. Velocity specific adaptations to three widely used strength training methods. Med. Sci. Sports Exerc. 2025.

**Disclaimer/Publisher’s Note:** The statements, opinions and data contained in all publications are solely those of the individual author(s) and contributor(s) and not of MDPI and/or the editor(s). MDPI and/or the editor(s) disclaim responsibility for any injury to people or property resulting from any ideas, methods, instructions or products referred to in the content.

### 2.62. Resistance Exercise Training for 24 Weeks Increases Maximal Dynamic Upper-Body Strength in Male and Female Wheelchair Users: A Randomized Controlled Multi-Site Study

Michelle van den Berg ^1^, Linn C Risvang ^2^ and Kristin L Jonvik ^2,^*

^1^  Faculty of Sport Sciences, Catholic University of Murcia, Spain; mvandenberg@alu.ucam.edu
^2^  Department of Physical Performance, Norwegian School of Sport Sciences, Norway; linncr@nih.no; kristinlj@nih.no
*  Correspondence: kristinlj@nih.no

**Abstract**

Upper-body muscle strength is essential for mobility, functional independence, and quality of life in individuals who rely on manual wheelchairs. Although resistance training has been shown to improve muscular strength and performance, most existing studies have focused exclusively on individuals with spinal cord injuries, limiting the generalizability of results to the broader population of wheelchair users. This randomized controlled multi-site study aimed to investigate the effects of a 24-week upper-body resistance training program on maximal dynamic and isometric strength in male and female wheelchair users with diverse disabilities. Thirty-six male (*n* = 24; age: 41 ± 9 y) and female (*n* = 12; age: 36 ± 9 y) were randomly assigned to either an intervention group, which completed supervised and independent upper-body resistance exercises three times per week, or a control group with no structured training. Muscle strength was assessed using four maximal voluntary isometric contraction tests (overhead press, prone row, supine pull, and bench press) and two 1-repetition maximum (1 RM) dynamic strength tests (overhead press and incline bench press) at baseline, 12 weeks, and 24 weeks. Dynamic strength significantly improved in the intervention group compared to control after 24 weeks for both 1 RM overhead press (intervention group: 20.3 ± 17.2% vs. control group: 2.1 ± 12.2%, *p* = 0.010) and 1 RM incline bench press (intervention group: 17.1 ± 13.1% vs. control group: −1.0 ± 13.7%, *p* < 0.001). In contrast, isometric strength increased similarly in both groups over time, with no significant group differences (*p* > 0.05). No sex differences were observed in relative strength improvement (*p* > 0.05), and strength gains were comparable across disability types (*p* > 0.05). These findings highlight that dynamic resistance training is an effective strategy for improving upper-body strength in a heterogeneous population wheelchair users, regardless of sex or underlying condition. In summary, this study supports the use of dynamic upper-body resistance exercise as a generalizable intervention to enhance physical performance in wheelchair users and underscores the need for future research on optimizing training protocols for maximal isometric strength adaptations.

**Keywords:** Spinal Cord Injury, Weight Training, Disability, Sex Differences

**Funding:** This research was funded by Foundation Dam, grant number 2022/FO387192.

**Disclaimer/Publisher’s Note:** The statements, opinions and data contained in all publications are solely those of the individual author(s) and contributor(s) and not of MDPI and/or the editor(s). MDPI and/or the editor(s) disclaim responsibility for any injury to people or property resulting from any ideas, methods, instructions or products referred to in the content.

### 2.63. Change of Direction and Curvilinear Sprint Inter-Limb Asymmetries and Deficits in Floorball

Līga Vecbērza ^1,^*, Līga Plakane ^2^ and Ilvis Ābeļkalns ^3,4^

^1^  University of Latvia, Faculty of Education Sciences and Psychology, Department of Teacher Education; liga.vecberza@lu.lv
^2^  University of Latvia, Faculty of Medicine and Life Science, Department of Human and Animal Physiology; liga.plakane.lu.lv
^3^  University of Latvia, Faculty of Education Sciences and Psychology, Department of Teacher Education; ilvis.abelkalns@lu.lv
*  Correspondence: liga.vecberza@lu.lv; Tel.: +371 27825919

**Abstract**

Floorball is a team sport that requires athletes to perform multi-directional movements from both the dominant (D) and non-dominant (ND) limbs, which increases injury risks (Liukkonen et al., 2024). However, there is limited evidence that floorball is an asymmetrical team sport. Therefore, the aim of this cross-sectional study was to (a) determine the magnitude of asymmetries in 45° change of direction (COD) as speed-based COD, 180° COD as strength-based COD and curvilinear sprint (CS) tasks and (b) examine asymmetry effects on performance times and deficits in floorball athletes. Thirty-seven athletes performed a 20 m linear sprint (LS) and 3-point line CS (6.75 m radius), with split time at 10 m, COD at 45° (45 COD) and 180° (180 COD) tests from each limb. The faster performance time side was defined as the D limb, but the slower as the ND limb. Inter-limb asymmetry indices (D—ND/D) were calculated as a percentage difference between limbs, but deficits using the equation (D and ND limb 10 m CS or COD time—10 m LS time). Spearman’s (ρ) coefficient was utilised to determine the association between all variables, the Kappa coefficient for the direction of asymmetry, and paired-sample *t*-tests to compare differences between the D and ND limbs. Significant differences were found between D and ND limbs in 180 COD times and 180 COD deficits (*p* < 0.001), as well as 10 m CS times and CS deficits (*p* < 0.05), but no differences in 45 COD times and 45 COD deficits. No significant associations were observed between 10 m LS time and 10 m CS and COD asymmetries. However, 10 m LS time showed a moderate to strong association between all performance times (ρ = 0.46 to 0.73, *p* < 0.01). Greater asymmetry was significantly associated with ND limb 180 COD time (ρ = −0.45, *p* < 0.01) and both D and ND limb 45 COD times (ρ = −0.33 to −0.41, *p* < 0.05), but not with 10 m CS times. Kappa coefficients revealed poor to slight levels of agreement in limb dominance between multi-directional tasks (Kappa = −0.11 to 0.12), thus demonstrating that asymmetries rarely tend to favour the same limb across tasks. These findings suggest that greater inter-limb asymmetry negatively affects performance in strength-based COD tasks, but does not significantly influence LS and CS performance. Practitioners should consider the magnitude of asymmetry when designing the training interventions.

**Keywords**: floorball; change of direction; curvilinear sprint; asymmetries; deficits

**Funding**: The research is financed by the Recovery and Resilience Facility project “Internal and External Consolidation of the University of Latvia” (No.5.2.1.1.i.0/2/24/I/CFLA/007).

**Reference**

1. Liukkonen, R.; Vaajala, M.; Tarkiainen, J.; Kuitunen, I. The incidence of floorball injuries-A systematic review and meta-analysis. Phys. Ther. Sport 2024, 67, 110–117.

**Disclaimer/Publisher’s Note:** The statements, opinions and data contained in all publications are solely those of the individual author(s) and contributor(s) and not of MDPI and/or the editor(s). MDPI and/or the editor(s) disclaim responsibility for any injury to people or property resulting from any ideas, methods, instructions or products referred to in the content.

### 2.64. Differences in Body Composition and Performance Characteristics Between Uphill and Flat Terrain Specialists in Cycling

Olav Vikmoen ^1,^*, Hege Nymo Ødemark ^1^, Kristin Lundanes Jonvik ^1^ and Truls Raastad

^1^  Department of Physical Performance, Norwegian School of Sport Sciences, Oslo, Norway
*  Correspondence: olavv@nih.no

**Abstract**

Body composition plays a crucial role in cycling performance (1). Success on uphill versus flat terrain demands different body composition and performance characteristics (2). However, the differences in performance metrics between climbers and flat specialists at the elite level, when adjusted for body composition factors, have not been thoroughly investigated. This study aims to characterize cycling performance, body composition and performance metrics differences between climbers and flat specialists, both in absolute terms and relative to specific aspects of body composition. Fourteen male cyclists from a UCI ProTeam were recruited for this study. Body composition, VO_2max_, W_max_ and Wingate was measured in all cyclists. Cyclists were categorized as climbers (CLIMB, n = 6) or flat specialists (FLAT, n = 6) based on rankings and discussions with the riders. Two cyclists identified as allrounders were excluded from these groups. CLIMB had lower body mass (BM) (65.2 ± 5.5 kg vs. 81.6 ± 6.5 kg, *p* ≤ 0.001), lower lean body mass (LBM) (55.2 ± 4.4 kg vs. 68.8 ± 6.6 kg, *p* = 0.002) and lower fat mass (FM) (8.1 ± 1.5 kg vs. 10.5 ± 1.6 kg, *p* = 0.002) than FLAT riders. No differences were observed in percent body fat (%FM) (12.5 ± 1.9% vs. 13.3 ± 2.2%, *p* = 0.533). In absolute values, CLIMB had lower VO_2max_ (5547 ± 391 mL·kg^−1^ vs. 6291 ± 507 mL·kg^−1^, *p* = 0.017) and W_max_ (472 ± 26 W vs. 529 ± 55 W, *p* = 0.043) than FLAT. However CLIMB had higher VO2_max_ and W_max_ when normalized for BM (VO_2max_: 85.3 ± 4.5 mL·kg^−1^·min^−1^ vs. 77.1 ± 1.7 mL·kg^−1^·min^−1^, *p* = 0.002, W_max_: 7.3 ± 0.5 W·kg^−1^ vs. 6.5 ± 0.2 W·kg^−1^, *p* = 0.004) and LBM (VO2max: 100.6 ± 3.8 mL·kg^−1^·min^−1^ vs. 91.5 ± 2.1 mL·kg^−1^·min^−1^, *p* ≤ 0.001, W_max_: 8.6 ± 0.4 W·kg^−1^ vs. 7.7 ± 0.2 W·kg^−1^, *p* ≤ 0.001). In absolute values Wingate peak and mean values was lower in CLIMB than in FLAT (Peak: 932 ± 232 W vs. 1276 ± 265 W, *p* = 0.037, Mean: 633 ± 110 W vs. 834 ± 130 W, *p* = 0.016), but no group differences were observed when normalizing for BM (Peak: 14.3 ± 3.5 W·kg^−1^ vs. 15.5 ± 2.3 W·kg^−1^, *p* = 0.504, Mean: 9.7 ± 1.8 W·kg^−1^ vs. 10.2 ± 0.9 W·kg^−1^, *p* = 0.601) or LBM (Peak: 16.8 ± 3.5 W·kg^−1^ vs. 18.4 ± 2.5 W·kg^−1^, *p* = 0.431, Mean: 11.5 ± 1.8 W·kg^−1^ vs. 12.1 ± 0.9 W·kg^−1^, *p* = 0.492). There was a strong positive correlation between LBM and VO_2max_ (r = 0.959). between LBM and W_max_ (0.905), between LBM and Wingate peak power (r = 0.867) and between LBM and Wingate mean power (r = 894). CLIMB riders were lighter and had lower LBM and FM compared to FLAT cyclists. Given the strong correlations between absolute VO_2max_ and W_max_ with LBM, it is not surprising that FLAT riders exhibited higher absolute VO_2max_ and W_max_. However, when normalized for BM and LBM, CLIMB had higher values.

**Keywords:** cycling performance; maximal oxygen consumption; body composition

**Funding:** This research received no external funding.

**References**

1. Carvalho de Moura, R.; de Moura Costa, C.; et al., Body composition and physical performance of mountain bike athletes. *Sci Rep* **2025**, *5*, 3329.
2. Lucia, A.; Joyos, H.; Chicharro, J.L. Physiological response to professional road cycling: climbers vs. time trialists. *Int J Sports Med* **2000**, *21(7)*, 505–12.

**Disclaimer/Publisher’s Note:** The statements, opinions and data contained in all publications are solely those of the individual author(s) and contributor(s) and not of MDPI and/or the editor(s). MDPI and/or the editor(s) disclaim responsibility for any injury to people or property resulting from any ideas, methods, instructions or products referred to in the content.

### 2.65. Associations Between Upper-Body Strength, Aerobic Capacity, and Performance in Cross-Country Skiers and Biathletes

**Carl-Maximilian Wagner ^1^, Øyvind Sandbakk ^2^, Stephan Schiemann ^1^ and Michael Keiner ^3^**

^1^  Institute of Exercise, Sport and Health, Leuphana University Lüneburg, Lüneburg, Germany
^2^  School of Sport Science, UiT The Artic University of Norway, Tromsø, Norway
^3^  Department of Sport Science, German University of Health and Sport, Ismaning, Germany

**Abstract**

Modern cross-country skiing places a greater emphasis on upper-body strength and power, especially in high-speed techniques like double poling (DP) and G3 skating. These techniques rely heavily on upper-body propulsion, so performance depends not only on aerobic capacity, but also on maximal muscular force. In upper-body dominant modalities, limited active muscle mass may constrain VO_2_ max expression, potentially rendering muscular strength a primary limiting factor. While prior studies have reported associations between upper-body strength and skiing performance, the findings are inconsistent, likely due to small sample sizes, methodological heterogeneity, and varying normalization approaches. Eighty-two cross-country skiers and biathletes (44 female, 38 male; age: 17 ± 2 years; VO_2max_: 63.2 ± 7.5 mL·kg^−1^·min^−1^) participated in this study. Upper-body strength was assessed via one-repetition maximum (1 RM) testing for the bench press and bench pull. Aerobic capacity was measured using a treadmill-based VO_2max_ test (running with poles at incline). DP performance was evaluated using a SkiErg with a 15-s all-out sprint and an incremental ramp protocol to exhaustion, yielding sprint power (DPP_15s_), peak power (DPP_peak_), and peak oxygen uptake (VO_2peak_-DP). Pearson and partial correlations (controlling for age and sex) were calculated. Mean 1 RM values were 48.9 ± 17.6 kg and 55.6 ± 17.0 kg in the bench press and bench pull, respectively. DPP_15s_ and DPP_peak_ were 253 ± 102 W and 174 ± 57 W, respectively. VO_2peak_-DP averaged 53.8 ± 8.4 mL·kg^−1^·min^−1^. Maximal strength was significantly correlated with power outputs (r = 0.90–0.93) and VO_2peak_-DP (r = 0.84). VO_2max_ was significantly correlated with both power (r = 0.87–0.92) and VO_2peak_-DP (r = 0.80). VO_2max_ and VO_2peak_-DP were highly correlated (r = 0.93). After adjusting for age and sex, partial correlations remained significant (r_p_ = 0.61–0.80), indicating robust associations independent of maturational or biological factors. Maximal upper-body strength appears to be a key determinant of power production and aerobic capacity during upper-body dominant techniques such as DP and G3. While VO_2max_ remains foundational, its translation into effective sport-specific output depends on the athlete’s capacity to generate muscular force. These results support systematic strength training as an integral component of performance development in modern cross-country skiing.

### 2.66. High-Volume Running Results in Decreased Fat Mass and Muscular Performance

Bjørk Wulff Helge ^1,2,^*, Kenneth Hudlebusch Mertz ^1,2^, Sara Dietz Pedersen ^1,2^, Jesper Løvind Andersen ^1,2^ and Jakob Agergaard ^1,2,^*

^1^  Institute of Sports Medicine Copenhagen, Department of Orthopaedic Surgery, Copenhagen University Hospital—Bispebjerg and Frederiksberg, Copenhagen, Denmark; bjoerk.wulff.helge@regionh.dk & jakob.agergaard@regionh.dk
^2^  Center for Healthy Aging, Department of Clinical Medicine, University of Copenhagen, Copenhagen, Denmark; bjoerk.wulff.helge@regionh.dk & jakob.agergaard@regionh.dk
*  Correspondence: BWH: bjoerk.wulff.helge@regionh.dk 00 4540470096; JA: jakob.agergaard@regionh.dk 004523392909

**Abstract**

In recent years ultramarathon running has become increasingly popular with more and more athletes competing in races longer than the traditional marathon distance (1). Preparing for ultramarathon races requires performing high-volume running thereby placing substantial loads on the lower limb muscles and tendons (2), and potentially increasing the risk of non-functional overreaching which has been linked to high-volume endurance training (3). The risk of non-functional overreaching can be exacerbated by low energy availability which is highly prevalent in long-distance running (4). High-volume running therefore likely leads to undesirable changes to body composition, muscle strength, and muscle power. This prospective cohort study aims to assess changes in body composition, explosive power, muscular performance in high-volume ultramarathon runners preparing for their primary race of the season. Thirty ultramarathon runners with a planned weekly running volume of more than 100 km/week completed three trial days carried out at the start of their training cycle, 3 months into their training cycle and at their maximum running volume prior to their primary race. Trial days consisted of blood sampling, DXA scanning, ultrasound imaging of muscle and tendons, bi- and unilateral counter movement jumps (CMJ), isokinetic strength testing, and a test of running economy and VO_2_-max. Concomitantly with each trial day participants performed a 3-day food diary and answered the Low Energy Availability in Males/Females—Questionnaire, Eating Disorder Examination Questionnaire, and Pittsburg Sleep Quality Index. Data was analyzed with linear mixed models using unstructured covariance matrices with lean body mass as a covariate for performance measurements. Fat mass decreased from visit 1 to visit 2 and 3 with no change in lean body mass. CMJ height decreased from visit 1 to visit 2 and 3 but peak force during CMJ only decreased at visit 3. Peak single leg CMJ force decreased significantly at visit 3 in the left leg and tended to in the right leg (*p* = 0.06). Peak torque at 60° decreased for the mean of the quadriceps but not the hamstrings at visit 2 and 3 compared to visit 1, and contrarily at 240° only the peak hamstring torque decreased at visit 2 and 3. In conclusion, prolonged high-volume running induces reduced fat mass, muscle power, and muscle strength highlighting the need for simultaneous resistance training to maintain muscular function throughout multiple race seasons. Future analysis will be aimed at examining whether low energy availability status can be linked to the reductions in performance observed during high-volume running.

**Keywords**: endurance training; running; isokinetic strength

**Funding**: This research was funded by Kulturministeriet, grant number SUAKPKfor2W.2024-020.

**References**

1. Scheer, V. Participation Trends of Ultra Endurance Events. *Sports Med Arthrosc* 2019, 27 (1), 3–7.
2. Sandbakk, Ø.; Haugen, T.; Ettema, G. The Influence of Exercise Modality on Training Load Management. *Int J Sports Physiol Perform* 2021, 16 (4), 605–608.
3. Meeusen, R.; Duclos, M.; Foster, C.; Fry, A.; Gleeson, M.; Nieman, D.; Raglin, J.; Rietjens, G.; Steinacker, J.; Urhausen, A.; European College of Sport Science; American College of Sports Medicine. Prevention, Diagnosis, and Treatment of the Overtraining Syndrome: Joint Consensus Statement of the European College of Sport Science and the American College of Sports Medicine. *Med Sci Sports Exerc* 2013, 45 (1), 186–205.
4. Whitney, K. E.; DeJong Lempke, A. F.; Stellingwerff, T.; Burke, L. M.; Holtzman, B.; Baggish, A. L.; D’Hemecourt, P. A.; Dyer, S.; Troyanos, C.; Adelzadeh, K.; Saville, G. H.; Heikura, I. A.; Farnsworth, N.; Reece, L.; Hackney, A. C.; Ackerman, K. E. Boston Marathon Athlete Performance Outcomes and Intra-Event Medical Encounter Risk Associated with Low Energy Availability Indicators. *Br J Sports Med* 2024, 59, 222–230.

**Disclaimer/Publisher’s Note:** The statements, opinions and data contained in all publications are solely those of the individual author(s) and contributor(s) and not of MDPI and/or the editor(s). MDPI and/or the editor(s) disclaim responsibility for any injury to people or property resulting from any ideas, methods, instructions or products referred to in the content.

### 2.67. Parameters of Balance, Motor Skills, and Fear of Falling in Untrained and Strength-Endurance Trained Elderly Women

Ľubica Žiška Böhmerová ^1,^*, Bianka Šiková ^2^ and Peter Schickhofer ^3^

^1^  Faculty of Physical Education and Sport, Comenius University Bratislava, Slovakia; lubica.bohmerova@uniba.sk
^2^  Faculty of Physical Education and Sport, Comenius University Bratislava, Slovakia; siko.bianca@gmail.com
^3^  Faculty of Physical Education and Sport, Comenius University Bratislava, Slovakia; peter.schickhofer@uniba.sk
*  Correspondence: lubica.bohmerova@uniba.sk; Tel.: +421-903-305-022

**Abstract**

In elderly adults, falls not only present a common cause of injury but can also create a psychological barrier due to increased fear of recurrent falls. Both effects may substantially compromise the subject’s autonomy and quality of life. The present study aimed to compare physical and psychological fall-related factors in trained versus untrained senior women, specifically examining differences in balance, motor and sensorimotor performance, and fear of falling, in relation to participation in regular strength-endurance training (2 h/week). Twenty women aged 60+ were divided into two groups (n = 10 each): trained and untrained. The trained group had an average age of 68.3 ± 5.9 years, body weight of 68.3 ± 6.9 kg, and height of 167.2 ± 3.7 cm. The untrained participants averaged 71.9 ± 6.4 years, 78.9 ± 9.3 kg, and 166.6 ± 2.9 cm. The trained group engaged in structured strength or functional training at least twice a week for a minimum of six months; the untrained group reported no regular physical activity. Assessments included postural stability (FitroSway stabilometric platform), neuromuscular control (“Driving test”), lower limb strength (Sit-to-Stand test), locomotor competence (10-m walk, stair ascent/descent), and subjective fear of falling (Falls Efficacy Scale International—FES-I). Trained women significantly outperformed untrained peers across multiple measures: Sit-to-Stand (14.4 vs. 9.2 reps; *p* ≤ 0.001), 10-m walk (5.44 s vs. 8.74 s; *p* ≤ 0.001), stair ascent (3.76 s vs. 6.85 s; *p* ≤ 0.001), and descent (3.29 s vs. 14.4 s; *p* ≤ 0.001), as well as neuromuscular control (*p* ≤ 0.01). In postural sway with eyes open, trained individuals had a significantly (*p* ≤ 0.001) lower center of gravity velocity (9.91 mm/s) compared to untrained individuals (15.04 mm/s). The trained group also reported a significantly lower index of fear of falling (FES-I: 22.3 vs. 31.1; *p* ≤ 0.01). The strongest correlation with fall-related fear was observed in the 10-m walk test (r = 0.67). Findings obtained highlight significant functional and psychological differences between trained and untrained senior women, suggesting that regular participation in strength-endurance training is associated with better balance, motor performance, and lower fear of falling.

**Keywords**: fall prevention; aging; physical activity; elderly women; fear of falling

**Funding**: This research was supported by VEGA Grant No.: 1/0393/23.

**Disclaimer/Publisher’s Note:** The statements, opinions and data contained in all publications are solely those of the individual author(s) and contributor(s) and not of MDPI and/or the editor(s). MDPI and/or the editor(s) disclaim responsibility for any injury to people or property resulting from any ideas, methods, instructions or products referred to in the content.

